# KCTD proteins regulate morphine dependence via heterologous sensitization of adenylyl cyclase 1 in mice

**DOI:** 10.1371/journal.pbio.3002716

**Published:** 2024-07-15

**Authors:** Zhong Ding, Chunsheng Zhang, Huicui Yang, Jiaojiao Chen, Zhiruo Sun, Xuechu Zhen

**Affiliations:** Jiangsu Key Laboratory of Neuropsychiatric Diseases and Department of Pharmacology, College of Pharmaceutical Sciences, Soochow University, Suzhou, China; Rutgers Robert Wood Johnson Medical School, UNITED STATES OF AMERICA

## Abstract

Heterologous sensitization of adenylyl cyclase (AC) results in elevated cAMP signaling transduction that contributes to drug dependence. Inhibiting cullin3-RING ligases by blocking the neddylation of cullin3 abolishes heterologous sensitization, however, the modulating mechanism remains uncharted. Here, we report an essential role of the potassium channel tetramerization domain (KCTD) protein 2, 5, and 17, especially the dominant isoform KCTD5 in regulating heterologous sensitization of AC1 and morphine dependence via working with cullin3 and the cullin-associated and neddylation-dissociated 1 (CAND1) protein. In cellular models, we observed enhanced association of KCTD5 with Gβ and cullin3, along with elevated dissociation of Gβ from AC1 as well as of CAND1 from cullin3 in heterologous sensitization of AC1. Given binding of CAND1 inhibits the neddylation of cullin3, we further elucidated that the enhanced interaction of KCTD5 with both Gβ and cullin3 promoted the dissociation of CAND1 from cullin3, attenuated the inhibitory effect of CAND1 on cullin3 neddylation, ultimately resulted in heterologous sensitization of AC1. The paraventricular thalamic nucleus (PVT) plays an important role in mediating morphine dependence. Through pharmacological and biochemical approaches, we then demonstrated that KCTD5/cullin3 regulates morphine dependence via modulating heterologous sensitization of AC, likely AC1 in PVT in mice. In summary, the present study revealed the underlying mechanism of heterologous sensitization of AC1 mediated by cullin3 and discovered the role of KCTD proteins in regulating morphine dependence in mice.

## Abstract 2

Heterologous sensitization of adenylyl cyclase (AC) contributes to drug dependence. This study shows that KCTD proteins 2, 5 and 17 regulate AC heterologous sensitization by enabling Cullin3 neddylation, and identifies a crucial role of this pathway in the paraventricular thalamic nucleus in regulating morphine dependence.

## Introduction

There are 10 isoforms of adenylyl cyclase (AC) encoded in mammalian cells, AC1 to AC10. AC1 to AC9 are membrane bonded. AC1, AC3, and AC8 are classified as Ca^2+^/calmodulin stimulated group, while AC5 and AC6 are inhibited by physiological concentrations of Ca^2+^. All these isoforms are expressed in rodent brain with isoform specific patterns [1–3].

Heterologous sensitization of AC (aka AC super-activation or cAMP overshoot) refers to a compensatory increase in AC activity upon persistence activation of Gαi/o-coupled GPCRs [4–6]. This phenomenon has long been proposed as a cellular mechanism underlying opioids dependence [4,5,7]. Opioids dependence is characterized as unpleasant physical and emotional responses after drug withdrawal [8] and increased cAMP signaling including PKA, CREB, and NMDARs plays a critical role in this process [7]. Chronic morphine exposure significantly enhanced the AC responses and cAMP signaling transduction in a series of brain regions such as nucleus accumbens (NAc), prefrontal cortex (PFC), and the thalamus nucleus [7,9–12]. Moreover, genetic knockout of AC1, AC5, or AC8 significantly attenuated physical signs of the mice in morphine withdrawal [13,14]. These observations revealed the significance of increased AC/cAMP signaling in morphine dependence; however, the detailed molecular mechanism associated with morphine-induced heterologous sensitization of AC and its significance in the development of morphine dependence is largely uncharted.

Activation of a Gαi/o coupled GPCR promotes the dissociation of Gαi/o subunit from Gβγ and persistent Gβγ signaling is essential for the development of heterologous sensitization of AC, for instance, in the cases of AC5 and AC6 [15–18]. The K^+^ channel tetramerization domain (KCTD) family consists of 25 members, which share a structurally similar bric-à-brac, tramtrack, and broad complex (BTB) domain [19]. At least half of the KCTD family has the capacity for Gβγ interaction [20]. Through interaction with Gβγ, KCTDs 8, 12, and 16 are involved in regulating GABA_B_ receptor signaling [21,22], while KCTDs 2, 5, and 17 regulate the cAMP signaling [23]. Biochemical and structural studies revealed that the C-terminal domains of KCTDs are mainly involved in binding Gβγ [20,24,25]. Expression respective KCTDs 2, 5, 17, which acts as Gβγ scavenger, strongly blocked heterologous sensitization of AC5 [20]. Besides Gβγ sequestrating, the KCTD also could act as substrate receptor of cullin3-RING ligases (CRL3s) for ubiquitination and degradation of substrate proteins including Gβγ [26,27]. Gβγ is reported to inhibit ACs 1, 3, 8 [28–30] but conditionally stimulates AC5 and AC6 [31], while knockdown respective KCTDs 2, 5, 17 elevated the expression of Gβγ and enhanced the activation of AC5 [23], revealing that KCTD family members may modulate AC activity through targeting Gβγ.

The cullin3 is a member of the cullin protein family, which scaffolds BTB and RING-box proteins to form fully assembled CRL3s [32]. The fully assembled CRL3s is functionally inactive and requires neddylation to be activated [33]. It is interesting to note that genetic knockdown of CRL3s or blocking the neddylation of CRL3s by MLN4924 completely blocks heterologous sensitization of ACs 1, 5, 6 [34], revealing the importance of CRL3s system in regulating heterologous sensitization of AC. Binding of the cullin-associated and neddylation-dissociated 1 (CAND1) protein to cullin is known to prevent the neddylation of cullin and subsequent ubiquitination of substrate proteins [35,36]. Interestingly, increased availability of substrate proteins may promote a dissociation of the CAND1-cullin complex and diminish the inhibitory effect of CAND1 on the neddylation of cullin [37]. These reports imply that CAND1 may be involved in heterologous sensitization of AC via mediating the neddylation of CRL3s.

The paraventricular thalamic nucleus (PVT), one of the major inputs of NAc, selectively mediates behavioral aversion [38]. AC1 is the major isoform of ACs in PVT [1,2]. Thus, it would be interesting to investigate how AC1 sensitization is regulated in PVT and its role in the development of morphine dependence.

In this study, we first examined the role and underlying mechanisms of the KCTD in heterologous sensitization of AC, particularly Ca^2+^/calmodulin stimulated ACs, in cellular models. We further revealed that heterologous sensitization of AC1 in PVT may contribute to the development of morphine dependence.

## Results

### The KCTD protein regulates the activation of adenylyl cyclase 1/6 (AC1/AC6)

Three different cell lines including μ-opioid receptor stably expressing HEK-293 cells (HEK-μR cells), HEK-μR cells transiently transfected with flag-AC1 (HEK-μR/AC1 cells), and SH-SY5Y cells stably expressing μ-opioid receptor (SH-SY5Y-μR cells) were constructed. Since AC6 contributes approximately 85% ATP catalyzing activity in HEK-293 cells [39], we employed HEK-μR cells to exam AC6 sensitization. HEK-μR/AC1 cells and SH-SY5Y-μR cells were employed to study heterologous sensitization of AC1 and endogenous Ca^2+^/calmodulin-stimulated Acs [40], respectively. The cAMP responses of respective cells to either morphine or calcium ionophore A23187 stimulation was shown in (S1A–S1C Fig). As expected, persistent activation of the μ receptor with morphine (10 μm) followed by stimulation with forskolin (1 μm) or A23187 (1 μm) in respective cells resulted in a 2- to 2.5-fold elevation of cAMP accumulation (S1D–S1F Fig), indicating the success in inducing heterologous sensitization.

In agreement with previous report [20], transfection of HA-KCTD5 plasmids barely affected the AC6 response (S1G Fig), while the AC6 response was not altered by 1 μm forskolin post siRNA knockdown of KCTD5 as well (S1G Fig). In contrast, we found that although knockdown of KCTD5 in HEK-μR/AC1 cells or SH-SY5Y-μR cells did not produce any effect on AC1 activity, overexpression of HA-KCTD5 dramatically boosted the AC1 activity stimulated by A23187 in a dose-related pattern (S1H and S1I Fig). These observations suggested that the KCTD may regulate the activation of AC in isoform-specific pattern, especially, overexpressing of KCTD5 could enhance AC1 activation.

### The KCTD mediates heterologous sensitization of AC1/AC6 through targeting Gβγ

**Fig 1:**
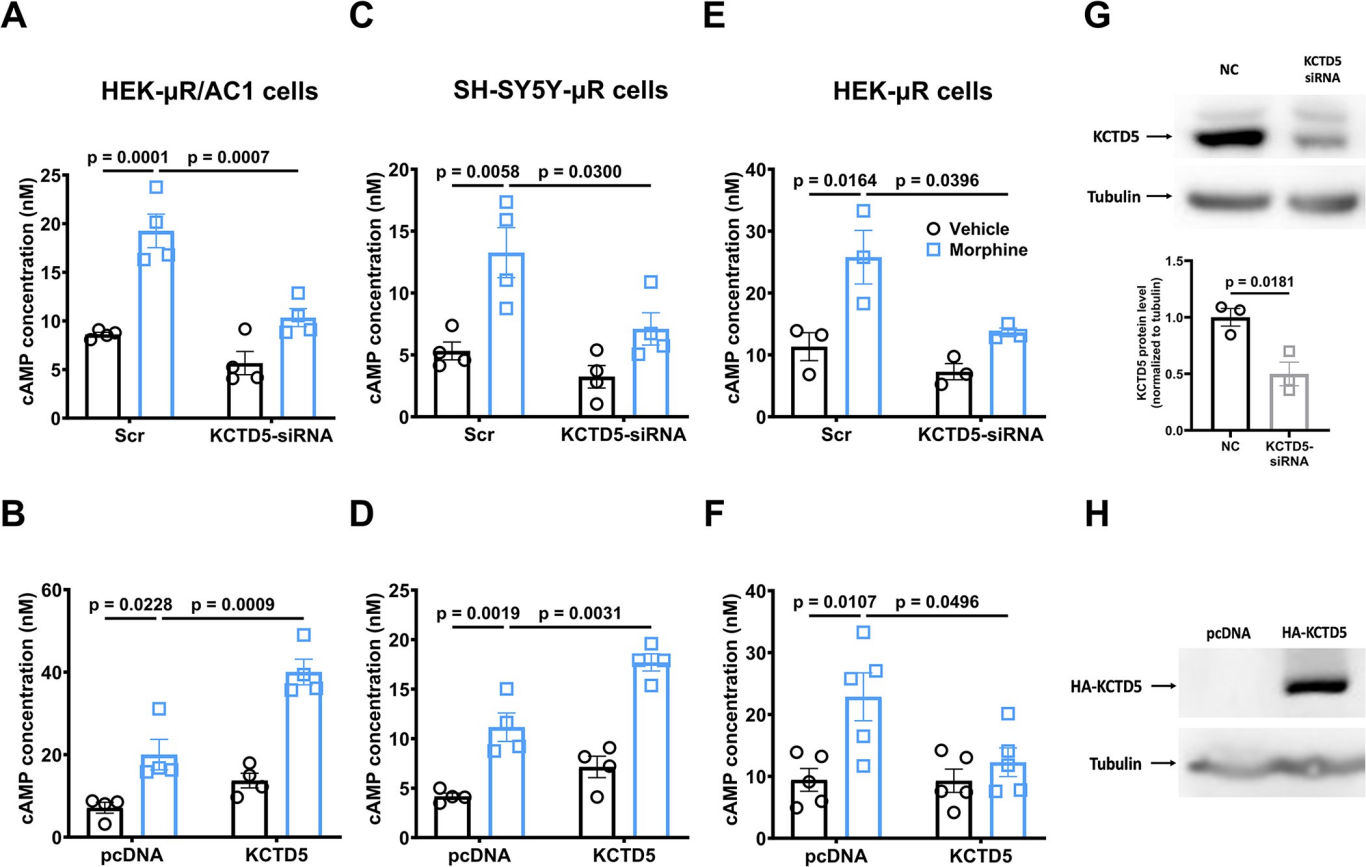
The KCTD5 mediates heterologous sensitization of AC. (**A, B**) HEK-μR/AC1, (**C, D**) SH-SY5Y-μR, or (**E, F**) HEK-μR cells were transfected with scrambled (Scr), siRNA targeting KCTD5 (KCTD5-siRNA), pcDNA vector (pcDNA), or HA-KCTD5 plasmid (KCTD5) as indicated. After 48 h transfection, cells were treated with either 10 μm morphine or vehicle for 2 h, followed by incubation with 1 μm A23187 (**A–D**) or 1 μm forskolin (**E, F**) in the presence of 10 μm naloxone and 500 μm IBMX for an additional 1 h. The cAMP concentration was determined by the cAMP detection kit. (**G, H**) Whole cell lysates were prepared from HEK-μR cells after transfection of Scr or KCTD5-siRNA, or pcDNA or HA-KCTD5 and probed with anti-KCTD2/5/17 or anti-HA antibodies. Data are representative of 3 independent experiments. The mean intensity of bands was quantified using Image J and normalized to their corresponding loading controls, *n* = 3. Comparisons between 2 groups were done using Student’s *t* test, and comparisons among multiple groups under 2 different conditions were performed using two-way ANOVA followed by Tukey’s test. Mean ± SEM. The data underlying the graphs shown in the figure can be found in S1 Data. AC, adenylyl cyclase; KCTD potassium channel tetramerization domain.

**Fig 2:**
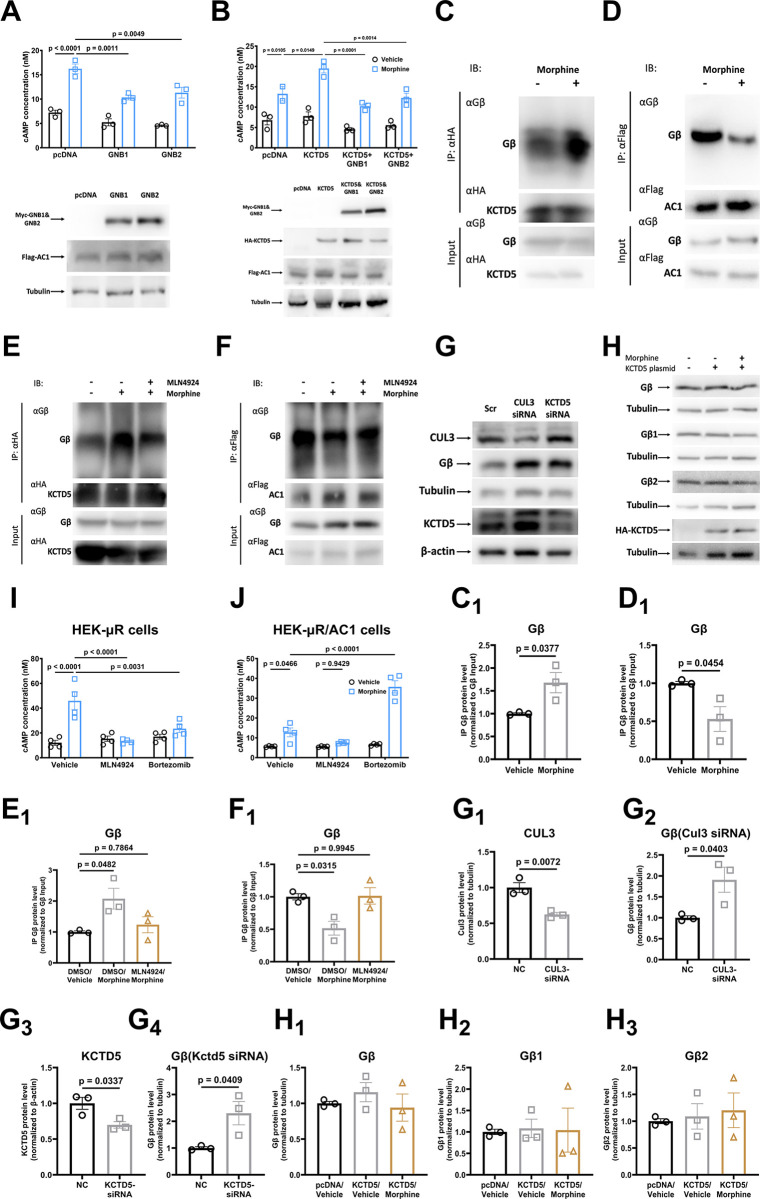
The KCTD5 mediates heterologous sensitization of AC1 through targeting Gβγ. (**A, B**) HEK-μR/AC1 cells were transfected with Myc-Gβ1, Myc-Gβ2, or HA-KCTD5 as indicated before AC sensitization protocol was applied to the cell. The expression of transfection was probed with anti-Myc and anti-HA antibody. HEK-μR cells were transfected with HA-KCTD5 (**C, E**) or Flag-AC1 (**D, F**) for 48 h. After transfection, the cells were pretreated with either 1 μm MLN4924 or vehicle for 30 min before being treated with 10 μm morphine or vehicle for another 2 h. Immunoprecipitation was performed to examine the interactions of Gβ with KCTD5 (**C, E**) and with AC1 (**D, F**) using anti-HA, anti-flag, or anti-Gβ antibodies. (**G**) HEK-μR cells were transfected with Scr, CUL3 siRNA, or KCTD5 siRNA. After 48 h transfection, whole cell lysate was probed with anti-CUL3, anti-KCTD2/5/17, or anti-Gβ antibodies. (**H**) HEK-μR cells were transfected with HA-KCTD5 or pcDNA before being treated for 2 h with vehicle or 10 μm morphine. Whole cell lysate was probed with anti-Gβ, anti-Gβ1, anti-Gβ2, or anti-HA antibodies. HEK-μR cells (**I**) or HEK-μR/AC1 (**J**) cells were pretreated with 1 μm MLN4924 or 1 μm bortezomib for 30 min before sensitization protocol was applied. Data are representative of 3 independent experiments. The mean intensity of bands was quantified using Image J and normalized to their corresponding input loadings (**C**_**1**_ **to F**_**1**_), or to their corresponding loading controls (**G**_**1**_ **to H**_**3**_), *n* = 3. Comparisons between 2 groups were done using Student’s *t* test. Comparisons among multiple groups under 1 condition were done using one-way ANOVA and comparisons among multiple groups under 2 different conditions were performed using two-way ANOVA followed by Tukey’s test. Mean ± SEM. The data underlying the graphs shown in the figure can be found in S1 Data. AC, adenylyl cyclase; KCTD potassium channel tetramerization domain.

To exam the role of KCTD in heterologous sensitization of AC1 in HEK-μR/AC1 cells, we included KCTDs 2, 5, 9, 17 since they all belong to a same group of the KCTD family [19]. The results revealed that respective KCTDs 2, 5, or 17 siRNA transfection all significantly blocked heterogeneous sensitization of AC1, while overexpression of respective KCTDs 2, 5, or 17, but not KCTD9, significantly enhanced heterologous sensitization of AC1 (Figs 1A, 1B, 1G, 1H, S2A, and S2E). Similarly, siRNA knockdown or overexpression of the dominant isoform KCTD5 blocked or boosted the heterologous sensitization of endogenous Ca^2+^/calmodulin-stimulated ACs in SH-SY5Y-μR cells, respectively (Fig 1C and 1D). As a control, we confirmed that KCTDs 2, 5, 17 siRNA all strongly inhibited heterogeneous sensitization of AC6 (Figs 1E and S2B). However, in consistent with previous report [20], overexpression of respective KCTDs 2, 5, 17, but not KCTD9, also significantly blocked AC6 sensitization (Figs 1F, S2 and 2F). Thus, these data indicated that KCTDs 2, 5, 17 may function as critical regulators of heterologous sensitization of AC, especially for AC1. Interestingly, we also found that overexpression of flag-GNB1 (gene for Gβ1) or flag-GNB2 (gene for Gβ2) strongly inhibited heterologous sensitization of AC1 (Fig 2A). Similarly, flag-GNB1 or flag-GNB2 transfection blocked the boosted AC1 activation in HA-KCTD5 overexpression cells (Fig 2B), indicating that the KCTD may exaggerate AC1 sensitization through regulating Gβγ. We also examined the effect of KCTD12 on AC1 sensitization, since KCTD12 could sequestrate Gβγ but lack E3 ligase activity. Our results showed that overexpression of KCTD12 increased AC1 sensitization while knockdown of KCTD12 had no effect (S3A–S3D Fig).

To investigate how KCTD5 regulates Gβγ, we examined the interactions of Gβ with AC1 as well as with KCTD5 in heterologous sensitization. The data showed that persistent activation of μ-opioid receptor in HEK-μR/AC1 cells induced a significant increase in the association between KCTD5 and Gβ (Fig 2C and 2C_1_). In contrast, we observed a significant dissociation of Gβ from AC1 with the same treatment (Fig 2D and 2D_1_). Application of AC sensitization inhibitor MLN4924 completely blocked heterologous sensitization, as shown in Fig 2E and 2E_1_, MLN4924 pretreatment abolished the morphine-induced increase on the association between Gβ and KCTD5. Similarly, MLN4924 pretreatment also attenuated the morphine-stimulated dissociation of Gβ from AC1 (Fig 2F and 2F_1_).

To confirm our observations on co-IP, we examined the co-localization of Gβ with KCTD5 or AC1. In good agreement with the co-IP data, chronic morphine exposure significantly increased and decreased the co-localization of Gβ with KCTD5 and AC1, respectively (S4A, S4A_1_, S4B and S4B_1_ Fig). Moreover, pretreatment with MLN4924 significantly blocked the effects of morphine on their co-localizations (S4A, S4A_1_, S4B and S4B_1_ Fig).

The turnover of Gβγ subunit is also an important factor in regulating heterologous sensitization. However, we detected neither changes in the degradation of the Gβ subunit following overexpression of HA-KCTD5 nor in the response to morphine treatment in HEK-μR cells (Fig 2G, 2G_1_–2G_4_, 2H and 2H_1_–2H_3_). Moreover, application of proteasome inhibitor bortezomib abolished heterologous sensitization of AC6 (Fig 2I), whereas bortezomib treatment strikingly boosted the AC1 sensitization (Fig 2J). Similarly, we found that chronic morphine exposure did not change Gβ level in the PVT tissues of the mice (S5 Fig). The data suggested that the change in turnover of Gβ appears unlikely to be the major mechanism underlying the KCTD5-mediated heterologous sensitization of AC1.

### CAND1 is involved in CUL3/KCTD5-mediated heterologous sensitization of AC1/AC6

**Fig 3:**
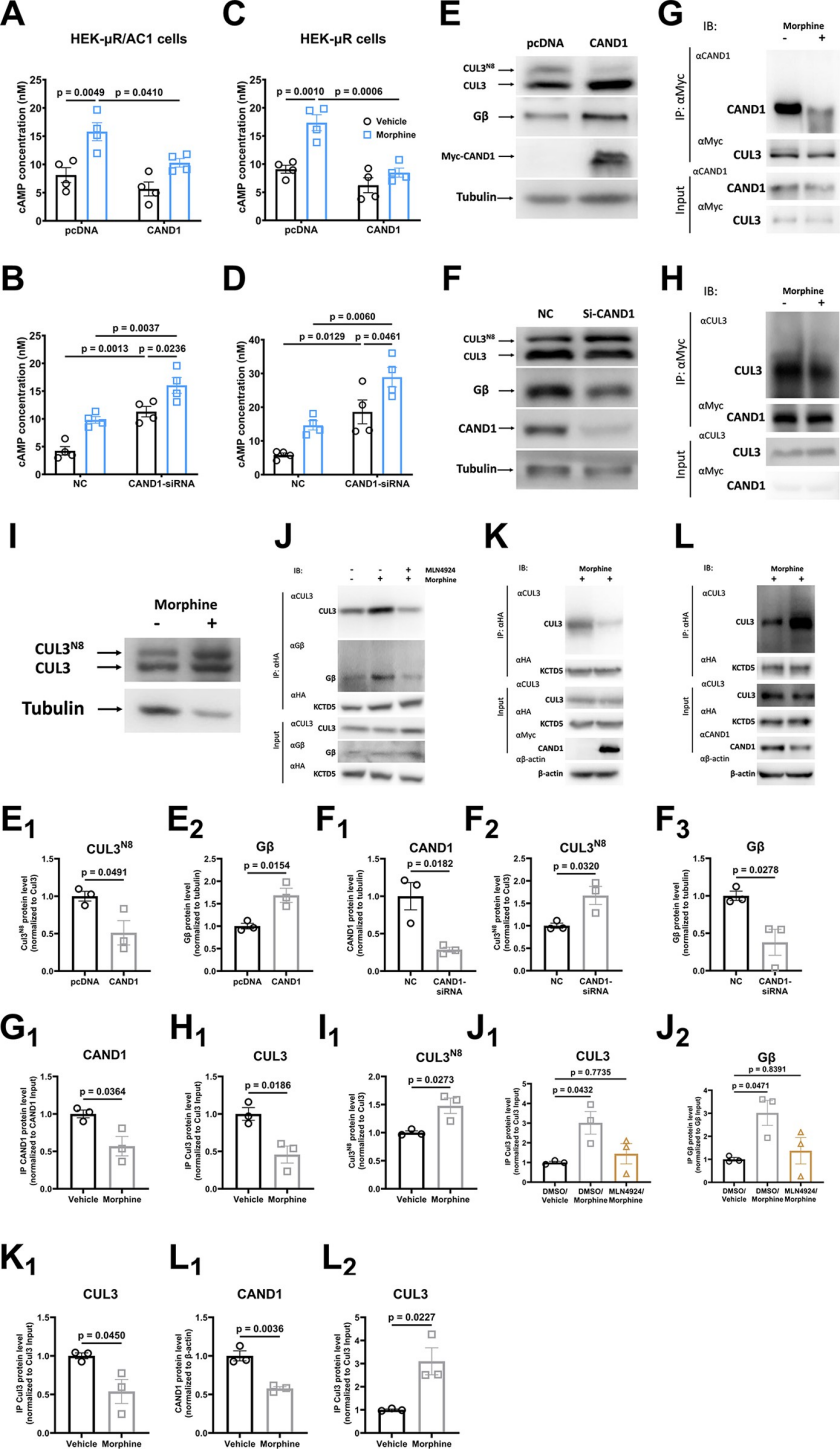
CAND1 is involved in CUL3/KCTD5-mediated heterologous sensitization of AC. HEK-μR/AC1 (**A, B**) or HEK-μR cells (**C, D**) were transfected with scrambled (Scr), siRNA targeting KCTD5 (KCTD5-siRNA), pcDNA vector (pcDNA), or HA-KCTD5 plasmid (KCTD5) as indicated before sensitization protocol was applied to the cell. (**E, F**) The overexpression or siRNA knockdown was probed with anti-myc or anti-CAND1 antibodies, and anti-CUL3 or anti-Gβ antibodies were used to probe CUL3/CUL3^N8^ or Gβ protein expression. HEK-μR cells were transfected with (**G**) myc-CUL3 or (**H**) myc-CAND1. After transfection, the cells were treated with 10 μm morphine or vehicle for 2 h before the cell lysis was prepared for co-IP to examine the interaction between CUL3 and CAND1. (**I**) HEK-μR cells were treated with vehicle or 10 μm morphine for 2 h. Whole cell lysis was used to probe CUL3/CUL3^N8^ protein with anti-CUL3 antibody. (**J**) HEK-μR cells were transfected with HA-KCTD5 before being pretreated with either 1 μm MLN4924 or vehicle for 30 min and treated with 10 μm morphine or vehicle for another 2 h. The cell lysis was prepared for co-IP to examine the interaction between KCTD5 with CUL3 or Gβ. HEK-μR cells were transfected with HA-KCTD5 in conjugation with pcDNA or myc-CAND1 (**K**), or with Scr or CAND1 siRNA (**L**). The cells were treated with 10 μm morphine for 2 h, and the interaction between KCTD5 and CUL3 was detected by co-IP. Data are representative of 3 independent experiments. The mean intensity of bands was quantified using Image J and normalized to their corresponding loading controls (**E**_**1**_ **to F**_**3**_**, I**_**1**_**, L**_**1**_), or to their corresponding input loadings (**G**_**1**_**, H**_**1**_**, J**_**1**_ **to K**_**1**_**, L**_**2**_), *n* = 3. Comparisons between 2 groups were done using Student’s *t* test. Comparisons among multiple groups under 1 condition were done using one-way ANOVA and comparisons among multiple groups under 2 different conditions were performed using two-way ANOVA followed by Tukey’s test. Mean ± SEM. The data underlying the graphs shown in the figure can be found in S1 Data. AC, adenylyl cyclase; CAND1, cullin-associated and neddylation-dissociated 1; KCTD potassium channel tetramerization domain.

We next explored the role of CAND1 in heterologous sensitization. In HEK-μR/AC1 and HEK-μR cells, overexpression of myc-CAND1 blocked heterologous sensitization of AC1 and AC6, while heterologous sensitization of AC1 and AC6 was enhanced with CAND1 knockdown (Fig 3A–3D). In consistent with the data, we observed that overexpression of myc-CAND1 inhibited cullin3 neddylation, whereas CAND1 knockdown resulted in the enhanced cullin3 neddylation (Fig 3E, 3E_1_, 3F, 3F_1_ and 3F_2_). It is worth noting that CAND1 knockdown also enhanced the activation of AC1 and AC6, suggesting that eliminating the expression of CAND1 could mimic the development of heterologous sensitization. Our data thus revealed that CAND1 is involved in heterologous sensitization AC1 and AC6.

To further elucidate the role of CAND1, we first examined the interaction between CUL3 with CAND1 or KCTD5 in heterologous sensitization. We found that chronic morphine exposure significantly promoted a dissociation of CUL3 from CAND1 and increased CUL3 neddylation in HEK-μR cells (Fig 3G–3I and 3G_1_–3I_1_). Meanwhile, we observed an increased interaction between KCTD5 and cullin3/Gβ which was almost completely blocked by MLN4924 pretreatment (Fig 3J, 3J_1_ and 3J_2_). These observations were consistent with our immunofluorescence double staining results in which chronic morphine treatment decreased the interaction of CUL3 with CAND1 but increased its interaction with KCTD5, and MLN4924 treatment inhibited the effects of morphine (S6A, S6A_1_, S6B and S6B_1_ Fig). We further demonstrated the role of CAND1 in regulating the interaction between KCTD5 and cullin3, as overexpression of myc-CAND1 induced the dissociation, whereas knockdown of CAND1 enhanced the association between KCTD5 and cullin3 (Fig 3K, 3K_1_, 3L, 3L_1_ and 3L_2_). Moreover, we found that knockdown of CAND1 decreased the expression of Gβ, while expression of myc-CAND1 elevated the Gβ expression (Fig 3E, 3E_2,_ 3F, 3F_1_ and 3F_3_), which is consistent with the data in Fig 2A in which the abundance of Gβ may be negatively correlated with AC1 sensitization. Taken together, our findings thus revealed that the enhanced interaction of the KCTD with both Gβ and cullin3 could promote the dissociation of CAND1 from cullin3, which removed the inhibition of CAND1 on CRL3s functions, and resulted in heterologous sensitization of AC1.

### Treatment of MLN4924 suppresses cAMP accumulation and morphine dependence in vivo

**Fig 4:**
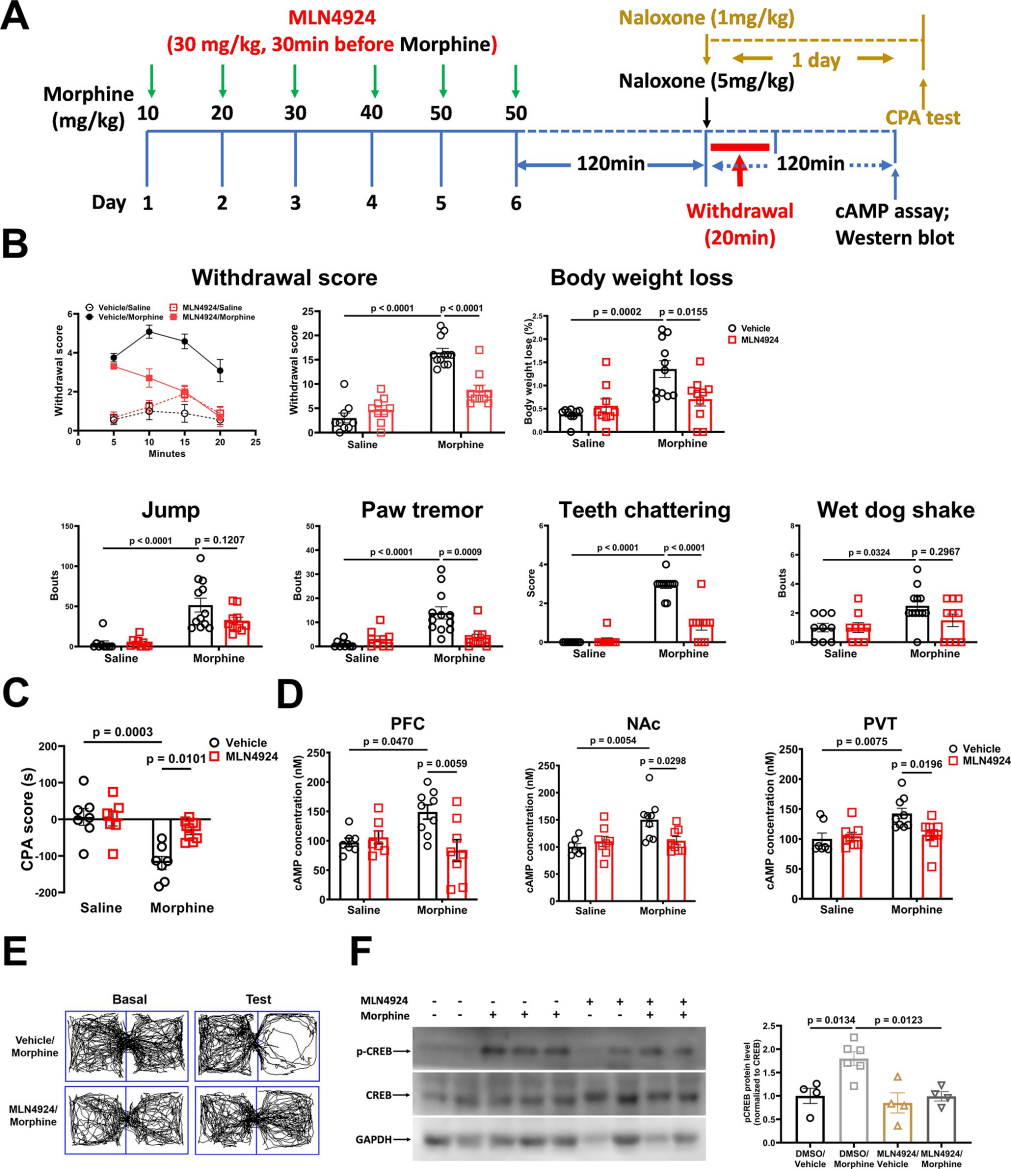
Treatment of MLN4924 suppresses cAMP accumulation and morphine dependence in vivo. (**A**) Experimental timeline for panels B to F. (**B, C**) Jumping, paw tremor, teeth-chattering, wet-dog shakes behaviors were videotaped and analyzed. The body weight loss, withdrawal score, and CPA score were calculated as described in the methods. (**D**) The PFC, NAc, and PVT tissue of the morphine-dependent mice and controls were dissected, and the cAMP levels were directly measured as described in the methods. (**E**) Representative place preference tracks for the mice with different treatment. (**F**) The PVT tissue of the morphine-dependent mice and controls were dissected for WB analysis probed by anti-CREB and anti-pCREB antibodies. The mean intensity of bands was quantified using Image J and normalized to their corresponding experimental controls, then one-way ANOVA followed by Tukey’s test was applied, *n* = 3. Two-way ANOVA followed by Bonferroni’s test was applied to behavioral results, *n* = 7–12. Mean ± SEM. The data underlying the graphs shown in the figure can be found in S1 Data. NAc, nucleus accumbens; PFC, prefrontal cortex; PVT, paraventricular thalamic nucleus.

We next examined the effects of MLN4924 on morphine dependence in mouse model of physical dependence and place aversion. MLN4924 could easily penetrate the brain–blood barrier [41] which was administered by intraperitoneal injection (i.p.). As shown in Fig 4B and 4C, MLN4924 administration robustly attenuated the morphine withdrawal responses and inhibited the CPA in morphine-dependent mice. The cAMP levels in different brain regions including NAc, PFC, and PVT were detected in morphine withdrawal mice 2 h after naloxone administration. As expected, naloxone injection evoked a strong increase of cAMP in tested brain regions of morphine-dependent mice as compared to that of drug-mice, whereas MLN4924 pretreatment dramatically attenuated the naloxone-induced increase of cAMP (Fig 4D). Moreover, MLN4924 also significantly suppressed the morphine withdrawal-enhanced expression of phosphorylated CREB (p-CREB) in PVT (Fig 4F). Our data provided the first evidence that suppression of AC sensitization by MLN4924 inhibited the development of morphine dependence in mice.

### KCTD5 mediates heterologous sensitization of AC in PVT and morphine dependence

**Fig 5:**
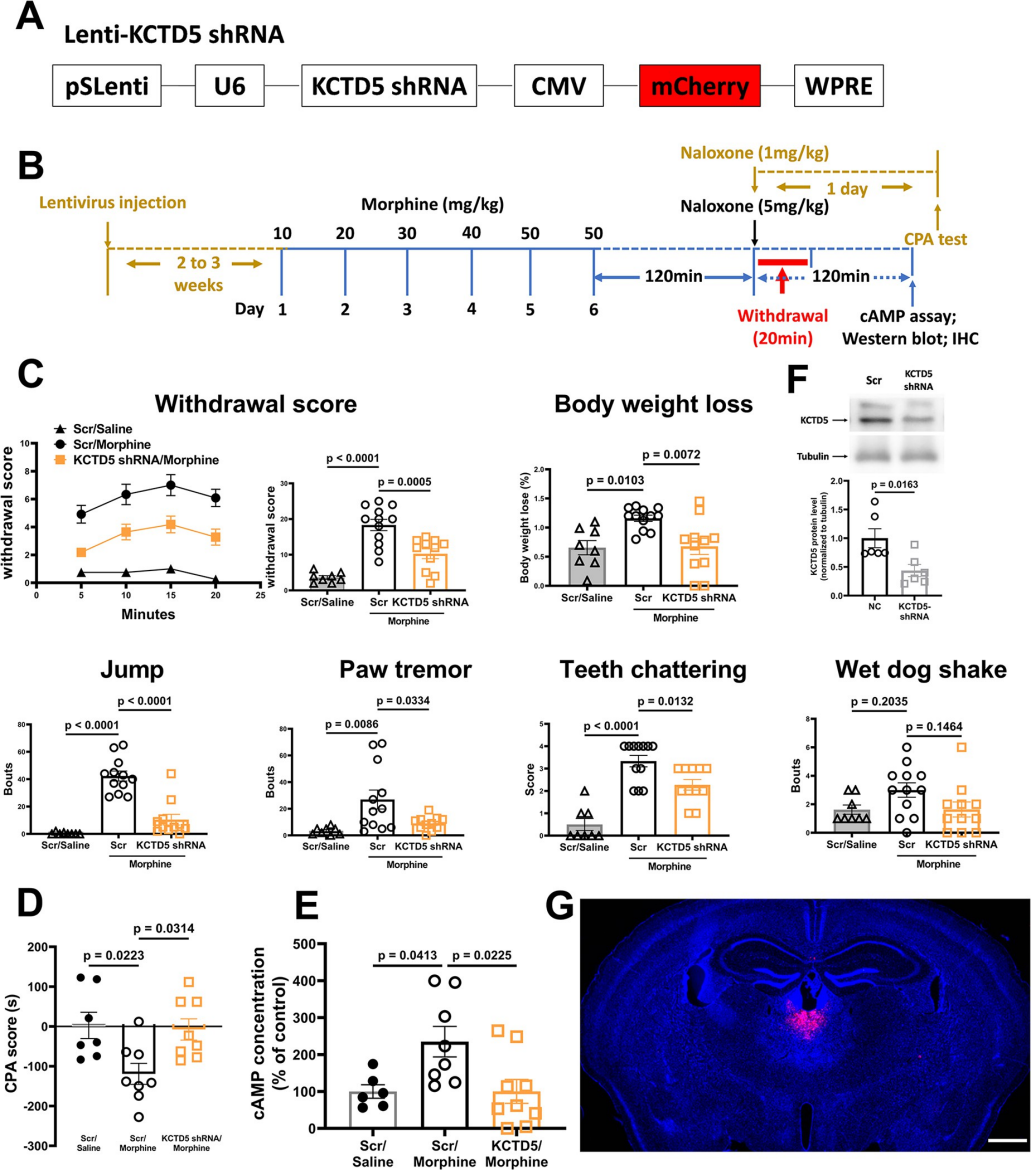
Knockdown of KCTD5 in PVT blocks local AC sensitization and morphine dependence in mice. (**A**) Schematics of the Lentivirus-KCTD5 shRNA. (**B**) Experimental timeline for panels C to G. (**C, D**) Mice withdrawal signs were videotaped and analyzed. The body weight loss, withdrawal score, and CPA score were calculated as described in the methods. (**E**) The PVT tissue of the morphine-dependent mice and controls were dissected, and the AC activity was measured as described in the methods. (**F**) The PVT tissue was dissected, and the expression of KCTD5 was probed with anti-KCTD2/5/17 antibody to check the efficiency of RNAi knockdown. The mean intensity of bands was quantified using Image J and normalized to corresponding loading controls, then one-way ANOVA followed by Tukey’s test was applied, *n* = 3. (**G**) Representative image for showing the site of injection. Two-way ANOVA followed by Bonferroni’s test was applied to behavioral results, *n* = 6–12. Mean ± SEM. Scale bar, 1 mm. The data underlying the graphs shown in the figure can be found in S1 Data. AC, adenylyl cyclase; KCTD potassium channel tetramerization domain; PVT, paraventricular thalamic nucleus.

PVT is known to be involve in drug dependence, especially aversive responses during drug withdrawal [38,42,43]. To reveal the role of AC sensitization of PVT in morphine dependence, mice were injected with specific KCTD5 shRNA-expressing lentivirus locally into the PVT 3 weeks before receiving escalating morphine treatment (Fig 5A and 5B). As shown in Fig 5C and 5D, elimination of KCTD5 in PVT markedly reduced the withdrawal scores and CPA in morphine-dependent mice. As expected, KCTD5 knockdown significantly suppressed the morphine withdrawal-induced AC sensitization (Fig 5E). The knockdown efficiency of KCTD5 protein in PVT and injection site was confirmed by immunoblots and fluorescence image (Fig 5F and 5G).

**Fig 6:**
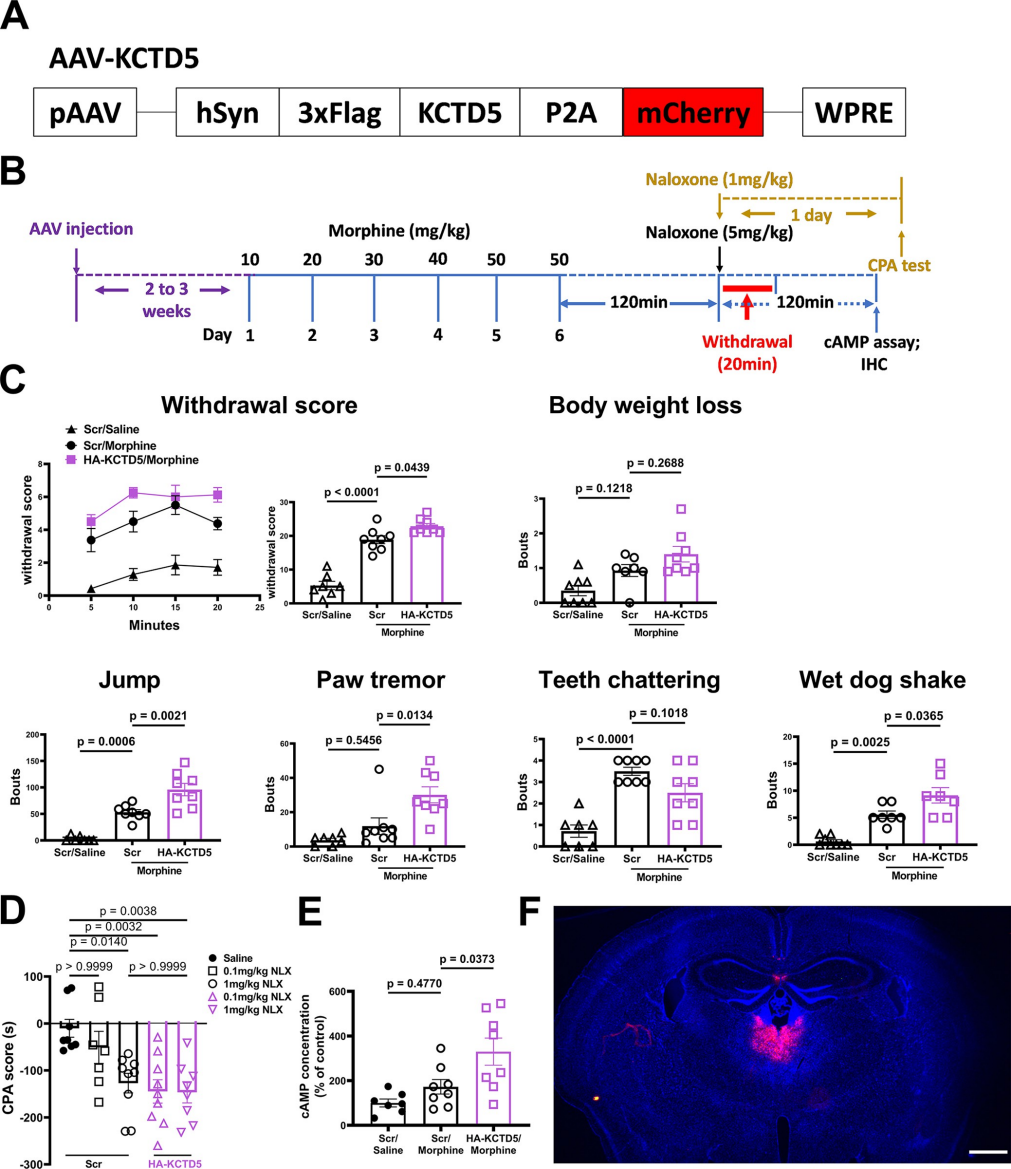
Overexpression of KCTD5 in PVT enhances local AC sensitization and sensitized the morphine dependence in mice. (**A**) Schematics of the AAV-KCTD5. (**B**) Experimental timeline for panels C to F. (**C, D**) Mice withdrawal signs were videotaped and analyzed. The body weight loss, withdrawal score, and CPA score were calculated as described in the methods. (**E**) The PVT tissue of the morphine-dependent mice and controls were dissected, and the AC activity was measured as described in the methods. (**F**) Representative image for showing the site of injection. Two-way ANOVA followed by Bonferroni’s test. Mean ± SEM. (*n* = 7–9). Scale bar, 1 mm. The data underlying the graphs shown in the figure can be found in S1 Data. AC, adenylyl cyclase; KCTD potassium channel tetramerization domain; PVT, paraventricular thalamic nucleus.

We next injected KCTD5-expressing AAV locally in PVT for 2 to 3 weeks before applying escalating morphine regimen to the mice (Fig 6A and 6B). Overexpression of KCTD5 strikingly exacerbated withdrawal signs and scores of the morphine-dependent mice (Fig 6C). In addition, we found that local KCTD5 overexpression significantly sensitized the aversive motivational valence of morphine-dependent mice, since a low dose of naloxone (0.1 mg/kg, i.p.) evoked the robust CPA in KCTD5-AAV injected mice as compared to control ones (Fig 6D). Moreover, comparing with controls, overexpression of KCTD5 in PVT also resulted in a significant increase in the AC response to morphine (Fig 6E). Lastly, fluorescence image confirmed the accuracy of the KCTD5-AAV injection in PVT (Fig 6F). These observations provided evidence that KCTD5 in PVT is critical in mediating morphine dependence, likely via modulation of AC sensitization.

### CUL3 is involved in mediating heterologous sensitization of AC in PVT in morphine dependence

**Fig 7:**
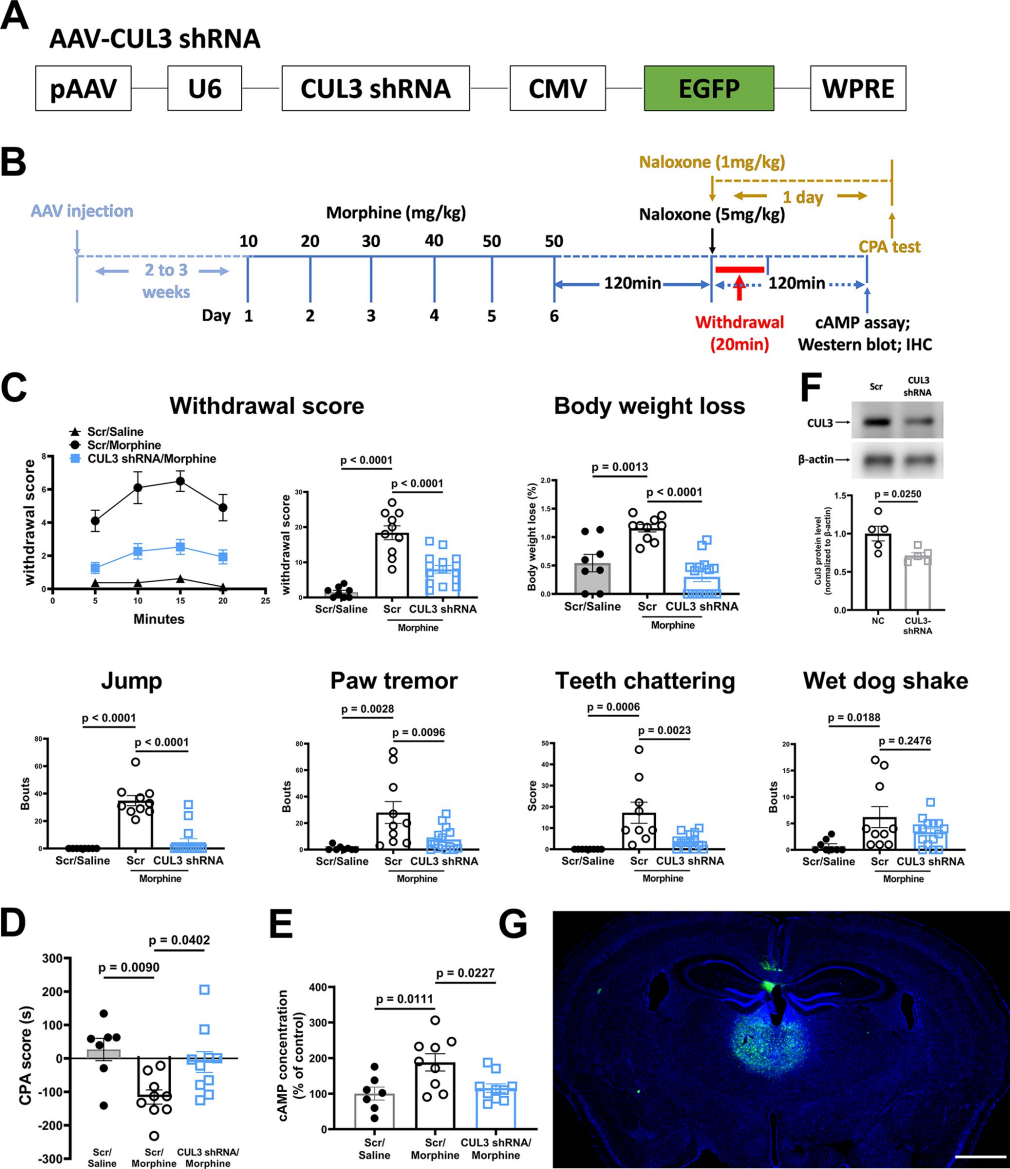
Knockdown of CUL3 in PVT blocks local AC sensitization and morphine dependence in mice. (**A**) Schematics of the AAV-CUL3 shRNA. (**B**) Experimental timeline for panels C to G. (**C, D**) Mice withdrawal signs were videotaped and analyzed. The body weight loss, withdrawal score, and CPA score were calculated as described in the methods. (**E**) The PVT tissue of the morphine-dependent mice and controls were dissected, and the AC activity was measured as described in the methods. (**F**) The expression of CUL3 in PVT was examined using anti-CUL3 antibody to check the efficiency of RNAi knockdown. The mean intensity of bands was quantified using Image J and normalized to corresponding loading controls, then one-way ANOVA followed by Tukey’s test was applied, *n* = 3. (**G**) Representative image for showing the site of injection. Two-way ANOVA followed by Bonferroni’s test was applied to behavioral results, *n* = 7–11. Mean ± SEM. Scale bar, 1 mm. The data underlying the graphs shown in the figure can be found in S1 Data. AC, adenylyl cyclase; PVT, paraventricular thalamic nucleus.

The KCTD5 functions as substrate receptor of CUL3 to mediate heterologous sensitization of AC as revealed by our in vitro data and other studies. To further validate the role of KCTD5/CUL3 in AC sensitization of PVT in morphine dependence, 2 to 3 weeks after local injection of CUL3 shRNA-expressing AAV, the mice were exposed to escalating dose of morphine (Fig 7A and 7B). CUL3 knockdown in PVT blocked the withdrawal responses as well as the CPA in morphine-dependent mice (Fig 7C and 7D). Accordingly, the AC sensitization in PVT of CUL3 knockdown mice was diminished compared with controls (Fig 7E). Immunoblot analysis and fluorescence image indicated the knockdown of CUL3 in PVT and injection site of the AAV, respectively (Fig 7F and 7G). Taken together, the results demonstrated that CUL3/KCTD5 regulates morphine dependence via modulating the sensitization of AC in PVT.

### AC1 in PVT is involved in morphine dependence

**Fig 8:**
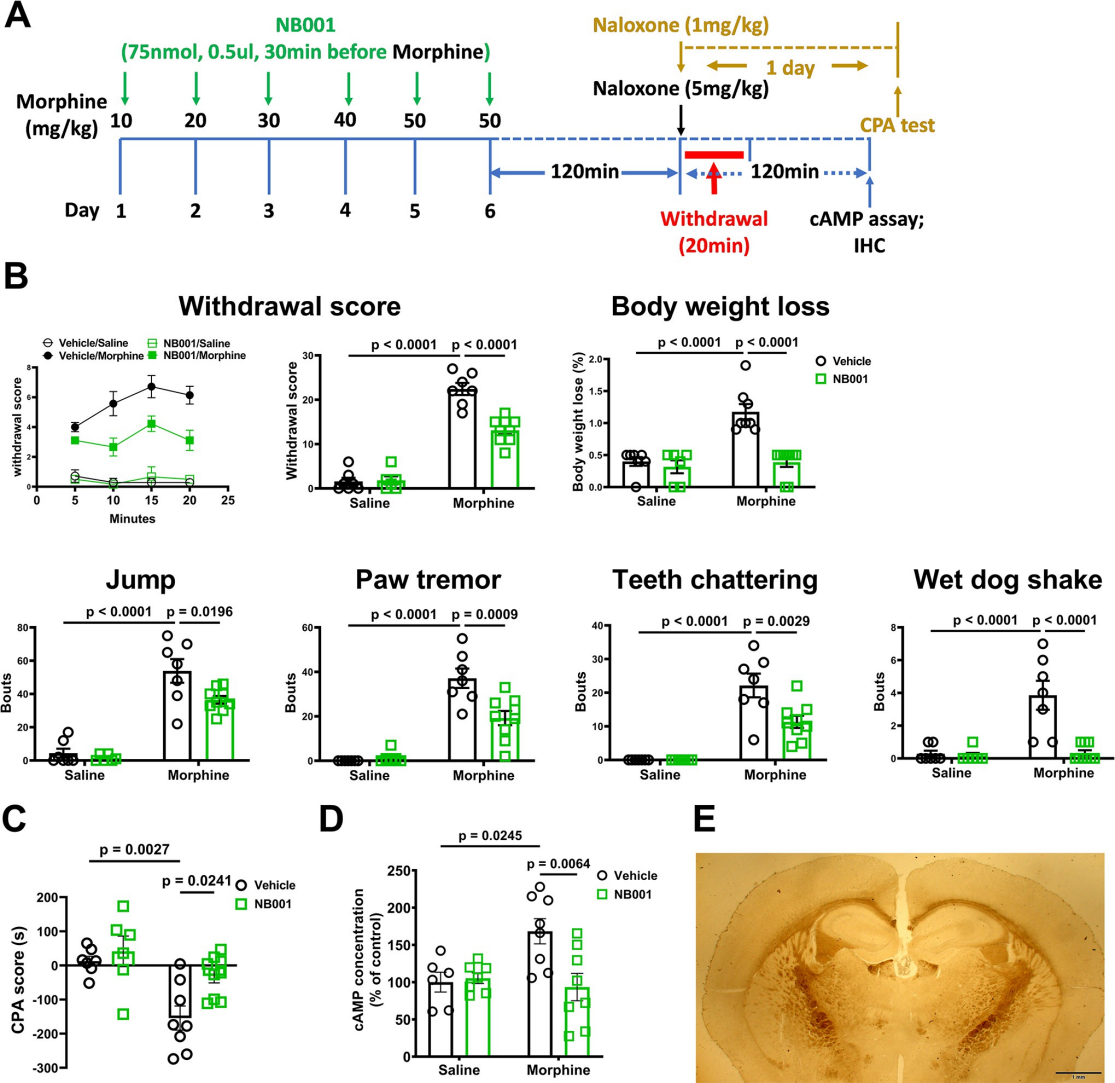
Inhibiting AC1 activity in PVT blocks local AC sensitization and morphine dependence in mice. (**A**) Experimental timeline for panels B to E. (**B, C**) Mice withdrawal signs were videotaped and analyzed. The body weight loss, withdrawal score, and CPA score were calculated as described in the methods. (**D**) The PVT tissue of the morphine-dependent mice and controls were dissected, and the cAMP levels were directly measured as described in the methods. (**E**) Representative image for showing the site of cannular implantation. Two-way ANOVA followed by Bonferroni’s test. Mean ± SEM. (*n* = 6–8). Scale bar, 1 mm. The data underlying the graphs shown in the figure can be found in S1 Data. AC, adenylyl cyclase; PVT, paraventricular thalamic nucleus.

Since AC1 is the dominant AC isoform expressed in PVT (S2 Table), we thus went further to examine if specific inhibition of AC1 activity will be sufficient to attenuate morphine dependence. Local infusion of AC1-specific inhibitor NB001 [44] into PVT significantly attenuated the withdrawal scores and CPA of morphine-dependent mice. This treatment also decreased the cAMP accumulation of PVT in response to chronic morphine treatment (Fig 8A–8D). Therefore, our data implicated that the sensitization of AC1 in PVT appears to mediate morphine dependence.

## Discussion

Heterologous sensitization of AC results in elevated cAMP signaling transduction that contributes to drug dependence. The cullin3 has recently been demonstrated as an essential regulator for AC sensitization, but the underlying mechanisms are undefined [34]. In the current study, we revealed the involvement of KCTD and CAND1, working in complex with cullin3, in mediating heterologous sensitization of AC, especially AC1. Moreover, we also provided the first evidence that KCTD5/cullin3 may regulate morphine dependence via modulating heterologous sensitization of AC1 in PVT.

One of the major findings of the current study is the elucidation of KCTD as critical regulator of heterologous sensitization of AC1. We found that overexpression of respective of KCTDs 2, 5, 17 significantly increased heterologous sensitization of AC1, while knockdown of the respective KCTDs largely blocked AC1 sensitization. Moreover, our findings suggested that the KCTDs may regulate AC1 sensitization through Gβγ sequestration. This conclusion is supported by the following observations: (1) neither overexpression of KCTD5 nor chronic morphine treatment resulted in accelerated turnover of Gβ in AC1 sensitization; (2) the enhanced association between KCTD5 and Gβ was companied by an increase in dissociation of Gβ from AC1 during heterologous sensitization; (3) the AC sensitization blocker MLN4924 significantly attenuated morphine-induced increase on the association between Gβ and KCTD5 as well as the dissociation of Gβ from AC1; (4) the boosted AC1 sensitization in KCTD5 overexpression cells was attenuated by complementary of the Gβ subunit; and (5) inhibiting proteasome-mediated protein degradation did not suppress AC1 sensitization. Except AC1, we observed that either overexpression or knockdown of respective of KCTDs 2, 5, 17 inhibited heterologous sensitization of endogenous ACs, mainly AC6, in HEK-293 cells. These observations are partially in agreement with a previous report that overexpression of KCTDs 2, 5, or 17, through Gβγ sequestration, strongly blocked heterologous sensitization of AC5 in HEK-293 cells [20]. Nevertheless, it should be noticed that although AC6 contributes approximately 85% ATP catalyzing activity, AC1 and AC3 are also expressed in HEK-293 cells [39], which could be inhibited by increased Gβγ after KCTDs knockdown. Therefore, we could not completely exclude the potential roles of other AC isoforms, since it was shown that knockdown of KCTDs 2, 5, or 17 elevated the activity of AC5 in mice primary neurons [23].

CAND1 was previously shown to regulate the neddylation of cullin [36,37,45]. We found that CAND1 expression was reversely associated with the neddylation of cullin3 as well as AC1/6 sensitization. Moreover, we observed that increased association of Gβ/KCTD5/cullin3 promoted the dissociation of CAND1 from cullin3 and enhanced cullin3 neddylation in heterologous sensitization of AC1/6. These findings are consistent with previous reports that binding of the CAND1 with cullin1/2 blocks their neddylation, while increased availability of substrates/substrate receptors promotes the dissociation of cullin/CAND1 complex and increases the neddylation of cullin1/2 [36,37,45]. In addition, in agreement with previous reports [28–30], our data also showed that the abundance of Gβ may be negatively correlated with AC1 activation and sensitization.

The Lys48 (K48)-linked polyubiquitination typically results in protein degradation, whereas monoubiquitylation or multi-ubiquitination may alter functions of the substrate without resulting a degradation [46]. For example, monoubiquitylated Gβ in yeast limited polarized growth of the cell but exhibited a normal stability [47]. In the present study, we cannot exclude the possibility that the CRL3s may regulate the development of heterologous sensitization through ubiquitinating the Gβ in a nondegradable way. However, the relation between the type of ubiquitination and degradation for Gβ is rather complex, since the degradation of monoubiquitinated Gβ has also been reported recently in HEK-293 cells [23]. Information on the functions of nondegradable ubiquitination of Gβ subunit are however limited, which requires more investigations to reveal their roles including that in heterologous sensitization of AC.

Another important finding of the present study is to uncover the roles of CUL3/KCTD5-mediated AC1 sensitization in morphine dependence. Knockdown or overexpression of the KCTD5 in PVT significantly attenuated or sensitized local AC responses in morphine-dependent mice, respectively. Similarly, knockdown of cullin3 in PVT robustly blocked local AC sensitization as well. These in vivo data is in line with our observations with regard on the role of KCTD5/cullin3 in regulating AC1 sensitization in cellular models. Importantly, our data showed that the alterations of AC responses in PVT were coordinated with the development of morphine dependence. Moreover, we observed that local infusion of selective AC1 inhibitor into PVT was sufficient to attenuate the development of morphine dependence, indicating AC1 could be the dominate isoform in PVT involved in heterologous sensitization of AC that was responsible for the development of morphine dependence. It is worth noting that heterologous sensitization of AC has been reported in different brain regions including PFC, basolateral amygdala (BLA), and ventral hippocampus (vHipp) [9–12]. In contrast to that of PVT, their projections to NAc are mainly related to rewarding [38,48]. Therefore, it will be interestingly to examine the role of the KCTD5/cullin3-mediated AC sensitization in these brain regions in drug rewarding in the future.

In summary, our findings may shed a light on our understanding of CRL3s-mediated heterologous sensitization of AC and provide the first direct evidence that heterologous sensitization of AC1 in PVT gates aversive states that associated with opiate dependence. Moreover, heterologous sensitization of AC is shared by most Gαi/o-coupled receptors including mu/kappa/delta opioid, D2/4 dopamine, alpha2 adrenergic, M2/4 muscarinic, and 5HT1A serotonin, which may render our findings of general physiological and therapeutic importance in different AC-linked GPCR systems.

## Methods and materials

### Cell lines and animals

HEK-293 cells stably expressing the μ-receptor (HEK-μR and HEK-μR/AC1) and SH-SY5Y cells stably expressing the μ-receptor (SH-SY5Y-μR) were constructed and maintained in DMEM (Gibco, Thermo Fisher Scientific) supplemented with 5% fetal clone I serum (Hyclone, Logan, Utah, United States of America), 1% Antibiotic-Antimycoctic (Life Technologies), and 1 mg/ml puromycin (Sigma). All cell lines were cultured at 37°C with 5% CO_2_.

Male adult C57BL/6 mice (10 to 14 weeks of age) were used for behavioral tests (purchased from Shanghai SLAC Laboratory Animal Co.). Mice were group-housed four/cage under a 12-h light-dark cycle (light on from 6 AM to 6 PM) in stable conditions with food and water ad libitum. All animal studies and experimental procedures were approved by the Animal Care and Use Committee of Soochow University and were following Guidelines for the Care and Use of Laboratory Animals (Chinese National Research Council, 2006, ethical approval number: 202208A0688) and the “ARRIVE” (Animals in Research: Reporting In Vivo Experiments) guidelines.

### Antibodies

The following antibodies were used: anti-CUL3 (SAB4200180, Sigma), anti-KCTD2/5/17 (15553-1-AP, Proteintech), anti-CAND1 (#8759, Cell Signaling Technology), anti-β-actin (#3700, Cell Signaling Technology), anti-tubulin (T5168, Sigma), anti-GAPDH (ab9485, Abcam), anti-Nedd8 (ab81264, Abcam), anti-CREB (#9197, Cell Signaling Technology), anti-pCREB (#9198, Cell Signaling Technology), anti-HA (51064-2-AP, Proteintech), anti-Flag (F7425, Sigma), anti-Gβ (sc-166123, Santa Cruz), anti-Gβ1 (10247-2-AP, Proteintech), and anti-Gβ2 (16090-1-AP, Proteintech).

### DNA plasmid and siRNA transfections

Briefly, DNA plasmids were first diluted in DMEM to the desired concentrations. Lipofectamine 2000 (Thermo Fisher Scientific) was diluted in Opti-MEM according to the manufacturer’s protocol and incubated for 5 min. DNA and Lipofectamine solutions were mixed, followed by incubation at room temperature for 30 min, and added to the cells dropwise. Cells were transfected for approximately 48 h prior to being used for the cAMP/immunoblot assays.

Similarly, siRNAs were diluted in DMEM to the desired concentrations (30 pmol final for 6-well plate). RNAiMAX (Thermo Fisher Scientific) was diluted in DMEM media, incubated for 5 min, and mixed with siRNA. Mixture of Lipofectamine and siRNAs was incubated at room temperature for 30 min before adding to the cells dropwise.

### Immunoprecipitation and western blot

For immunoprecipitation assays, cell extracts or brain tissue homogenates were prepared in lysis buffer (50 mM HEPES (pH 7.4), 150 mM NaCl, 5 mM EDTA, 0.1% Triton, X-100) containing 1 mM DTT and protease inhibitor cocktail (Sigma), and 150~200 μg of lysate were incubated with 1.5 μg of antibodies overnight at 4°C, followed by incubation with Protein A/G Magnetic Beads (Selleck) for 4 h at room temperature. The precipitates were washed 3 times with a washing buffer (50 mM Tris, 150 mM NaCl, 0.1% Triton, X-100, pH 7.5). The immunoprecipitated proteins were eluted in 1× loading buffer (NCM Biotech) and boiled for 5 min before applying for electrophoresis.

For western blot, 15~25 μg proteins were loaded and separated by SDS-PAGE gel (10%) electrophoresis before transferring onto a PVDF membrane. The primary and secondary antibodies were used under the instructions of manufacturers with a dilution rate from 1:1,000 to 1:10,000. ECL reagents were applied to visualize bands with ChemiScope 3300 Mini (CLINX, Shanghai, China).

### cAMP assays in cells and brain tissues

To induce heterologous sensitization, the cells were subjected to either vehicle or μ-receptor agonist morphine (10 μm final concentration) for 2 h at 37°C. The culture plate was equilibrated to room temperature for 30 min before addition of forskolin or A23187 with 500 μm IBMX and 10 μm μ-receptor antagonist naloxone for 1 h. The reaction was stopped by the addition of the homogenous time-resolved fluorescence cAMP detection reagents, D2-labeled cAMP, and Cryptate labeled anti-cAMP antibody (Cisbio, Bedford, Massachusetts, USA). Fluorescence (Ex 330/80, Em 615/10 and 665/7) was measured on the EnVision 2104 Multilabel Reader (PerkinElmer) under the instructions of manufacturers. To determine the relative cAMP accumulation per well, the ratio of 665 nm/615 nm fluorescence values was calculated.

For cAMP assays in mice brain tissues, the brains of the mice with morphine dependence or controls were quickly removed on ice. Then, the desired brain regions were dissected in cold PBS under the stereoscope and stored in −80°C before the assay. The tissue was thawed on ice and homogenized ultrasonically in membrane preparation buffer (10 mM Tris, 5 mM EDTA, pH 7.4) and the protein concentrations were determined using BCA assay kit. The cAMP level in respective brain regions was directly measured in 384-well plate using Cisbio cAMP assay kit for 20 μg tissue lysates. Alternatively, crude membranes were prepared after homogenizing the tissue in membrane preparation buffer by centrifugation at 16,000g for 15 min at 4°C. Then, the membranes were resuspended in assay buffer (75 mM Tris-HCl (pH 7.4), 15 mM MgCl_2_, 2 mM EDTA, 500 mM IBMX), and the protein concentration was determined using BCA assay kit. Membranes pellet (15 μg per well) was added to a 384-well plate and 3 μm calmodulin (final concentration) was added in stimulation buffer (75 mM Tris-HCl (pH 7.4), 15 mM MgCl_2_, 250 μm ATP, 1 μm GppNHp, 500 μm IBMX, and 500 μm CaCl_2_−10 μm free Ca^2+^) and incubated at room temperature for 1 h. The cAMP concentration was measured according to the manufacture instruction using Cisbio cAMP assay kit at last [11,49].

### Quantitative real time-PCR

Total RNA was extracted from mouse PVT tissues using RNAiso Plus (TaKaRa, Tokyo, Japan) according to the manufacturer’s instructions. The RNA (1 μg) was reverse transcribed into cDNA using the TaRaKa reverse transcription kit with Oligo (dT) primer and cDNA was amplified using the specific primers (Mouse) listed in S1 Table. A StepOnePlus Real-Time PCR System (Applied Biosystems, Carlsbad, California, USA) was used to quantify mRNA expression using SYBR Premix II (TaKaRa, Tokyo, Japan). The parameters for quantitative real-time PCR were 30 s at 95°C, 5 s at 95°C, and 30 s at 60°C for 40 cycles. GAPDH was used as a reference gene.

### Stereotactic injection and histology

The AAV-KCTD5-P2A-mCherry, Lenti-shRNA (KCTD5)-mCherry, and AAV-shRNA (CUL3)-EGFP viruses were purchased from Obio Technology. For AAV vector, serotype 9 was used in this study and all the plasmids were designed and constructed by standard methods. For virus injection, mice were anesthetized with ketamine (100 mg/kg of body weight) and xylazine (8 mg/kg) by i.p. injection and placed in a stereotactic frame. Mice were injected with 0.6 μl of AAV virus (approximately 10^12^ infection units per ml) or 1 μl of lentivirus (approximately 10^8^ infection units per ml) into the PVT (coordinates from bregma: −1.1 mm AP, 0 mm ML, −3.2 mm DV) [43] using glass microelectrodes at a slow rate (approximately 25 nl/min). The injection microelectrode was slowly withdrawn 5 min after the virus infusion. Behavior experiments were performed 2 to 3 weeks after surgery. The injection sites were examined at the end of the tests and only data obtained from animals with correct injections were included. Brain slices from mice were counterstained with Hoechst and directly examined under fluorescent microscope after mounting on the slides.

### Naloxone-induced morphine withdrawal and conditioned place aversion

Mice after AAV injection were allowed to freely explore both sides of a CPP training apparatus for 15 min to assess their baseline place preference. Mice with AAV injection received a single daily injection of morphine (i.p.) for 6 consecutive days with doses escalating at 10, 20, 30, 40, 50, and 50 mg/kg in their home cage to develop morphine dependence. For another group of animals, MLN4924 were administrated 30 min before each dose of the morphine injections. Two hours after the last morphine injection, mice received either a 5 mg/kg naloxone (i.p.) injection to induce withdrawal responses or 1 mg/kg naloxone injection to induce CPA. The mice were placed in a transparent cylinder and withdrawal responses were videotaped for 20 min. Jumping, paw tremor, teeth-chartering, wet-dog shakes were recorded and scored from 0 to 3 based on behavioral bouts (0 = absent; 1, 1–3 bouts; 2, 4–6 bouts; 3, ≥7) at 5-min intervals. The total bouts of each behavior and the global withdrawal scores were calculated [50]. The body weight loss was calculated in the following formula: (weight before withdrawal but immediately after naloxone injection–weight immediately after withdrawal)/weight before withdrawal but immediately after naloxone injection*100%. Alternatively, the mice were confined in the counterbalanced side of the CPP chamber for 45 min post 1 mg/kg naloxone injection. One day after naloxone injection, withdrawal mice were re-exposed to the CPP chamber and allowed to explore both sides of the CPP chamber for 15 min. The CPA score was calculated by subtracting the time spent in the aversive stimulus-paired side of the chamber during baseline from the time spent in the same side of the chamber during the test.

### Statistical analysis

All data are presented as mean ± SEM (*n* ≥ 3). Comparisons between 2 groups were done using Student’s *t* test. Comparisons among multiple groups under 1 condition were done using one-way ANOVA and comparisons among multiple groups under 2 different conditions were performed using two-way ANOVA. The Tukey’s and Bonferroni’s multiple-comparison test were used as indicated in the legends for post hoc analyses. For quantification of immunofluorescence double staining, Mander’s coefficient value was analyzed using Fiji software.

## Supporting information

S1 Fig Construction of HEK-μR, SH-SY5Y-μR, and HEK-μR/AC1 cells and examination the role of KCTD5 on AC activations. HEK293 and SH-SY5Y cells were transfected with μ-opioid receptor to establish stable expressing cells that are termed HEK-μR and SH-SY5Y-μR cells, respectively. Flag-AC1 was transiently transfected in HEK-μR cells, which is termed HEK-μR/AC1 cells. (**A**) HEK-μR/HEK-293 cells and (**B**) SH-SY5Y-μR/SH-SY5Y cells were incubated with 5 μm forskolin in the presence of morphine at indicated concentrations for 1 h and data is presented as % inhibition of forskolin stimulated AC activity. (**C**) HEK-μR-AC1/HEK-μR cells were incubated with A23187 at indicated concentrations for 1 h. Whole cell lysates were prepared from HEK-μR (pcDNA) and HEK-μR/AC1 (flag-AC1) cells and probed with anti-flag antibody. Sensitization protocol was applied to all cell lines with desired stimulator, (**D**) 1 μm forskolin, (**E, F**) 1 μm A23187. (**G**) HEK-μR, (**H**) HEK-μR/AC1, or (**I**) SH-SY5Y-μR cells were transfected with pcDNA, HA-KCTD5 plasmid, scrambled (Scr), or siRNA targeting KCTD5 as indicated. HEK-μR cells, HEK-μR/AC1 cells, or SH-SY5Y-μR cells were incubated with increasing concentrations of forskolin (**G**) or A23187 (**H, I**) in the presence of 500 μm IBMX for 1 h. Data are representative of 3 independent experiments. Two-way ANOVA followed by Tukey’s test. Mean ± SEM. The data underlying the graphs shown in the figure can be found in S1 Data.
(TIF)

S2 Fig Heterologous sensitization or protein expression examination after transfection of different siRNA or plasmids, or post drug pretreatment. HEK-μR/AC1 and HEK-μR cells were transfected with Scr, KCTD2 siRNA, or KCTD17 siRNA (**A, B**) or pcDNA or HA-KCTDs 2, 9, 17 (**E, F**). After transfection, sensitization protocol was applied to cells with desired stimulator. The anti-KCTD2/5/17 (**C, D**) or anti-HA (**G**) antibodies were used to probe the effect of siRNA knockdown or plasmids overexpression. Data are representative of 3 independent experiments. (**C**_**1**_**, D**_**1**_) The mean intensity of bands was quantified using Image J and normalized to their corresponding loading controls, then Student’s *t* test was applied, *n* = 3. Two-way ANOVA followed by Tukey’s test was applied to comparisons among multiple groups under 2 different conditions. Mean ± SEM. The data underlying the graphs shown in the figure can be found in S1 Data.
(TIF)

S3 Fig Effect of overexpression or knockdown of KCTD12 on heterologous sensitization of AC1. HEK-μR/AC1 cells were transfected with flag-KCTD12 (KCTD12) (**A**) or siRNA targeting KCTD12 (si-KCTD12) (**B**) as indicated. After 48 h transfection, cells were treated with either 10 μm morphine or vehicle for 2 h, followed by incubation with 1 μm A23187 in the presence of 10 μm naloxone and 500 μm IBMX for an additional 1 h. The cAMP concentration was determined by the cAMP detection kit. Whole cell lysates were prepared from the cells after transfection and probed with anti-flag antibody (**C**) or total mRNA were extracted and the KCTD12 mRNA were quantified by qPCR (**D**). Data are representative of 3 independent experiments. (**A and B**) Two-way ANOVA followed by Tukey’s test. (**D**) Unpaired *t* test. Mean ± SEM. The data underlying the graphs shown in the figure can be found in S1 Data.
(TIF)

S4 Fig Examining the co-localization of Gβ with KCTD5 and AC1 in heterologous sensitization. HEK-μR cells were transfected with HA-KCTD5 (**A**) or Flag-AC1 (**B**) for 48 h. After transfection, the cells were pretreated with either 1 μm MLN4924 or vehicle for 30 min before being treated with 10 μm morphine or vehicle for another 2 h. Immunofluorescence double staining was performed to examine the co-localization of Gβ with KCTD5 (**A**) and with AC1 (**B**) using anti-Gβ, anti-HA, or anti-flag antibodies. Images are representative of 3 independent experiments. The co-localization was quantified using Fiji software. Mander’s coefficient value was analyzed for HA-KCTD5 overlapping with Gβ (**A1**) and Flag-AC1 overlapping with Gβ (**B1**). One-way ANOVA followed by Tukey’s test. Mean ± SEM, *n* = 18 cells/3 independent experiments. Scale bar, 50 μm. The data underlying the graphs shown in the figure can be found in S1 Data.
(TIF)

S5 Fig Gβ expression in the PVT of morphine-dependent mice. The mice received a single daily injection of morphine (i.p.) for 6 consecutive days with doses escalating at 10, 20, 30, 40, 50, and 50 mg/kg in their home cage to develop morphine dependence. Two hours after the last morphine injection, the PVT tissue was quickly dissected on ice and the Gβ expression was examined by WB using specific anti-Gβ antibody. The mean intensity of bands was quantified using Image J and normalized to their corresponding loading controls, and Student’s *t* test was applied, *n* = 4.
(TIF)

S6 Fig Examining the co-localization of Cul3 with KCTD and CAND1 in heterologous sensitization. HEK-μR cells were transfected with Myc-Cul3 (**A**) or Myc-CAND1 (**B**) for 48 h. After transfection, the cells were pretreated with either 1 μm MLN4924 or vehicle for 30 min before being treated with 10 μm morphine or vehicle for another 2 h. Immunofluorescence double staining was performed to examine the co-localization of KCTD2/5/17 with Cul3 (**A**) and Cul3 with CAND1 (**B**) using anti-KCTD2/5/17, anti-Cul3, or anti-Myc antibodies. Images are representative of 3 independent experiments. The co-localization was quantified using Fiji software. Mander’s coefficient value was analyzed for Cul3 overlapping with KCTD2/5/17 (**A1**) and CAND1 overlapping with Cul3 (**B1**). One-way ANOVA followed by Tukey’s test. Mean ± SEM, *n* = 16 cells/3 independent experiments. Scale bar, 50 μm. The data underlying the graphs shown in the figure can be found in S1 Data.
(TIF)

S7 Fig The map of sites of cannular implantation. (TIF)

S1 Table The sequence of primers for RT-qPCR. (XLSX)

S2 Table AC isoform expression in mice PVT. The mRNA expression of AC1 to AC8 in PVT of C57 mice was examined by RT-qPCR (*n* = 6). The mRNA was considered as “undetectable” when the Ct value of certain AC isoform is larger than 32 for at least 3 out of 6 mice. The data underlying the graphs shown in the figure can be found in S1 Data.
(XLSX)

S1 Data Statistical details and numerical values. (XLSX)

S1 Raw Images Raw images of immunoblotting experiments. (PDF)

## Abbreviations

AC: adenylyl cyclase
BLA: basolateral amygdala
BTB: bric-à-brac, tramtrack, and broad complex
CAND1: cullin-associated and neddylation-dissociated 1
KCTD: potassium channel tetramerization domain
NAc: nucleus accumbens
PFC: prefrontal cortex
PVT: paraventricular thalamic nucleus
vHipp: ventral hippocampus
