# Supplementary figures and images for: KCTD proteins regulate morphine dependence via heterologous sensitization of adenylyl cyclase 1 in mice
Source: PLoS Biol. 2024 Jul 15;22(7):e3002716. doi: 10.1371/journal.pbio.3002716 (PMC11271871; doi:10.1371/journal.pbio.3002716)

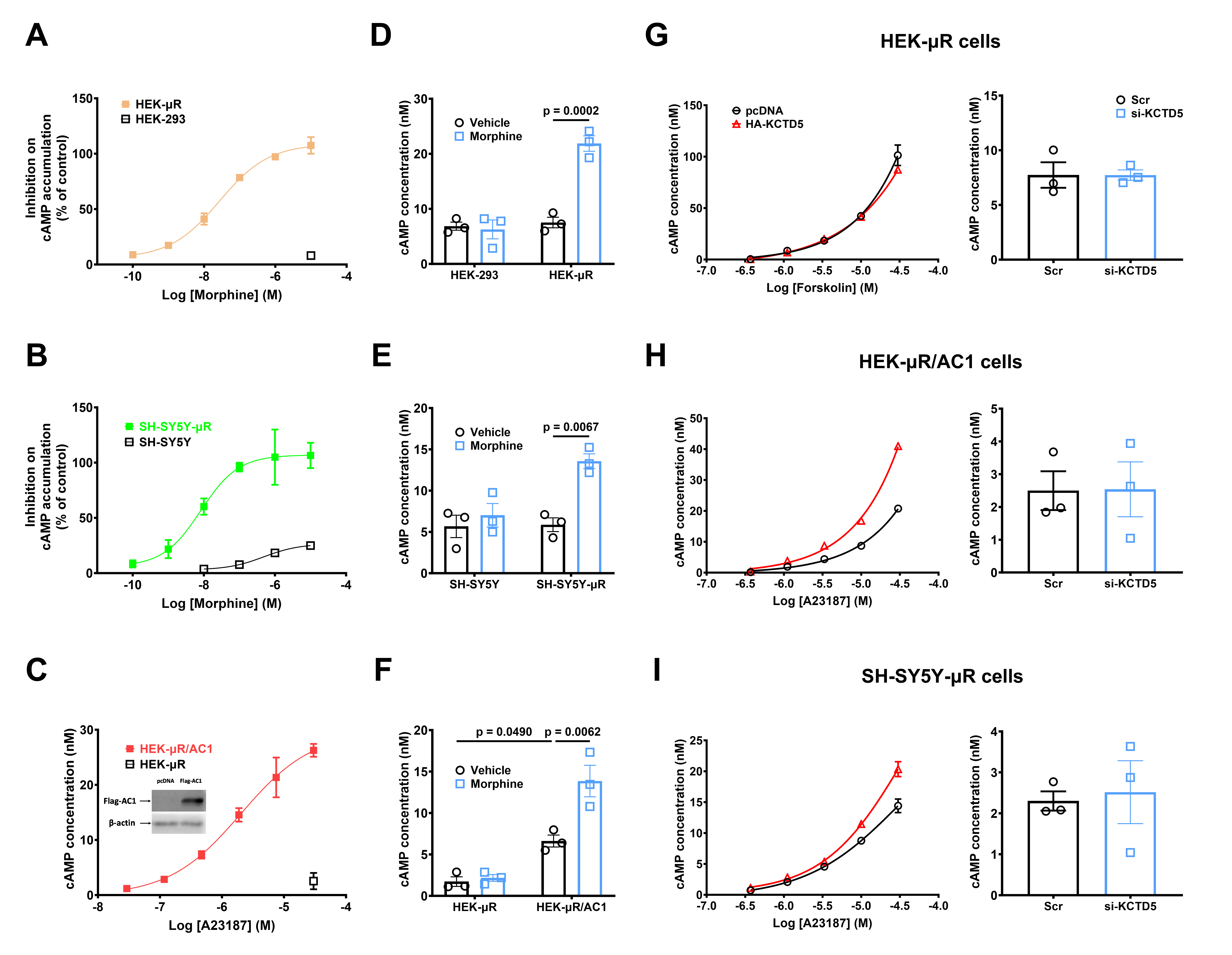

Supplement: S1 Fig — HEK293 and SH-SY5Y cells were transfected with μ-opioid receptor to establish stable expressing cells that are termed HEK-μR and SH-SY5Y-μR cells, respectively. Flag-AC1 was transiently transfected in HEK-μR cells, which is termed HEK-μR/AC1 cells. (A) HEK-μR/HEK-293 cells and (B) SH-SY5Y-μR/SH-SY5Y cells were incubated with 5 μm forskolin in the presence of morphine at indicated concentrations for 1 h and data is presented as % inhibition of forskolin stimulated AC activity. (C) HEK-μR-AC1/HEK-μR cells were incubated with A23187 at indicated concentrations for 1 h. Whole cell lysates were prepared from HEK-μR (pcDNA) and HEK-μR/AC1 (flag-AC1) cells and probed with anti-flag antibody. Sensitization protocol was applied to all cell lines with desired stimulator, (D) 1 μm forskolin, (E, F) 1 μm A23187. (G) HEK-μR, (H) HEK-μR/AC1, or (I) SH-SY5Y-μR cells were transfected with pcDNA, HA-KCTD5 plasmid, scrambled (Scr), or siRNA targeting KCTD5 as indicated. HEK-μR cells, HEK-μR/AC1 cells, or SH-SY5Y-μR cells were incubated with increasing concentrations of forskolin (G) or A23187 (H, I) in the presence of 500 μm IBMX for 1 h. Data are representative of 3 independent experiments. Two-way ANOVA followed by Tukey’s test. Mean ± SEM. The data underlying the graphs shown in the figure can be found in S1 Data. (TIF) [file pbio.3002716.s001.tif]

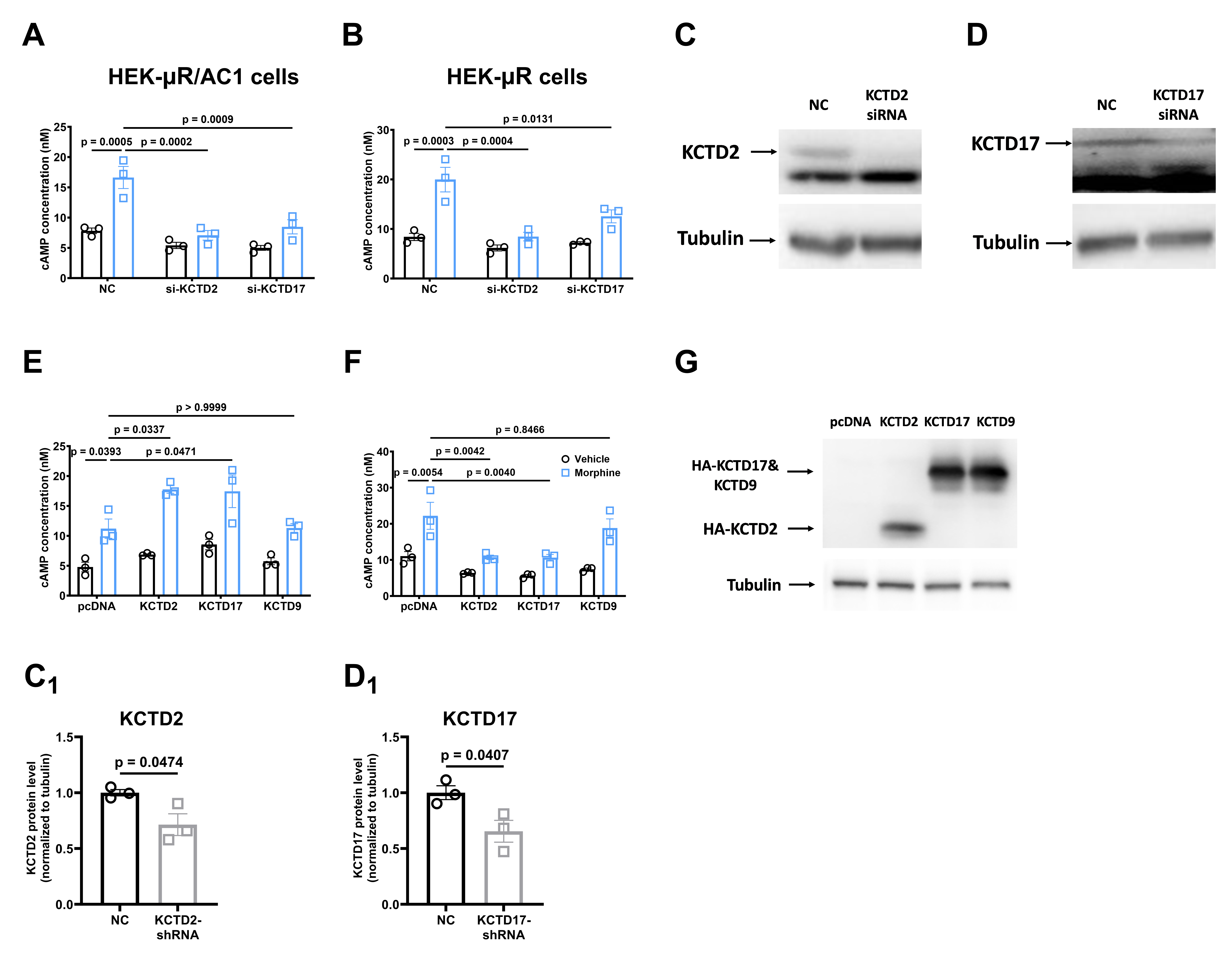

Supplement: S2 Fig — HEK-μR/AC1 and HEK-μR cells were transfected with Scr, KCTD2 siRNA, or KCTD17 siRNA (A, B) or pcDNA or HA-KCTDs 2, 9, 17 (E, F). After transfection, sensitization protocol was applied to cells with desired stimulator. The anti-KCTD2/5/17 (C, D) or anti-HA (G) antibodies were used to probe the effect of siRNA knockdown or plasmids overexpression. Data are representative of 3 independent experiments. (C1, D1) The mean intensity of bands was quantified using Image J and normalized to their corresponding loading controls, then Student’s t test was applied, n = 3. Two-way ANOVA followed by Tukey’s test was applied to comparisons among multiple groups under 2 different conditions. Mean ± SEM. The data underlying the graphs shown in the figure can be found in S1 Data. (TIF) [file pbio.3002716.s002.tif]

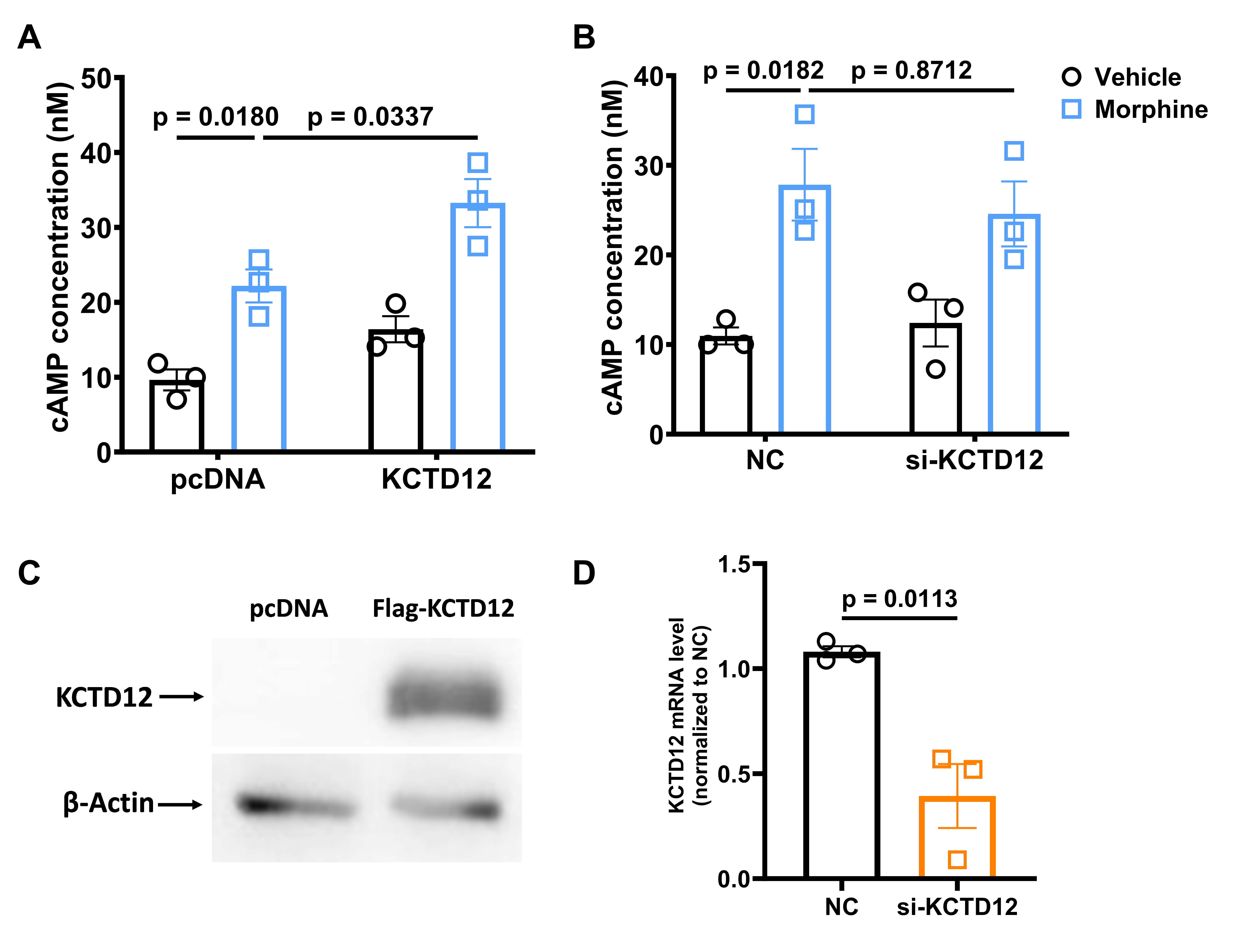

Supplement: S3 Fig — HEK-μR/AC1 cells were transfected with flag-KCTD12 (KCTD12) (A) or siRNA targeting KCTD12 (si-KCTD12) (B) as indicated. After 48 h transfection, cells were treated with either 10 μm morphine or vehicle for 2 h, followed by incubation with 1 μm A23187 in the presence of 10 μm naloxone and 500 μm IBMX for an additional 1 h. The cAMP concentration was determined by the cAMP detection kit. Whole cell lysates were prepared from the cells after transfection and probed with anti-flag antibody (C) or total mRNA were extracted and the KCTD12 mRNA were quantified by qPCR (D). Data are representative of 3 independent experiments. (A and B) Two-way ANOVA followed by Tukey’s test. (D) Unpaired t test. Mean ± SEM. The data underlying the graphs shown in the figure can be found in S1 Data. (TIF) [file pbio.3002716.s003.tif]

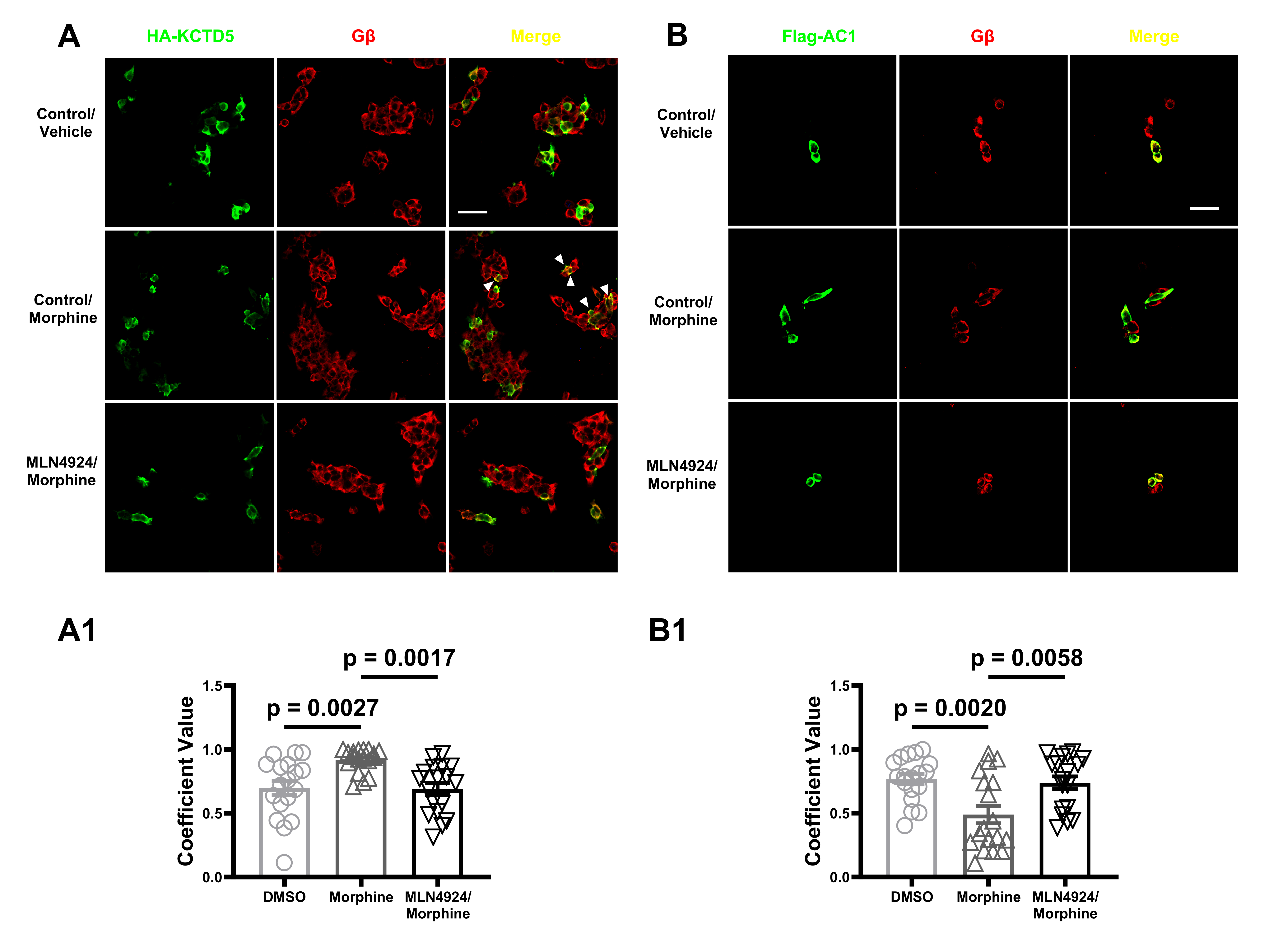

Supplement: S4 Fig — HEK-μR cells were transfected with HA-KCTD5 (A) or Flag-AC1 (B) for 48 h. After transfection, the cells were pretreated with either 1 μm MLN4924 or vehicle for 30 min before being treated with 10 μm morphine or vehicle for another 2 h. Immunofluorescence double staining was performed to examine the co-localization of Gβ with KCTD5 (A) and with AC1 (B) using anti-Gβ, anti-HA, or anti-flag antibodies. Images are representative of 3 independent experiments. The co-localization was quantified using Fiji software. Mander’s coefficient value was analyzed for HA-KCTD5 overlapping with Gβ (A1) and Flag-AC1 overlapping with Gβ (B1). One-way ANOVA followed by Tukey’s test. Mean ± SEM, n = 18 cells/3 independent experiments. Scale bar, 50 μm. The data underlying the graphs shown in the figure can be found in S1 Data. (TIF) [file pbio.3002716.s004.tif]

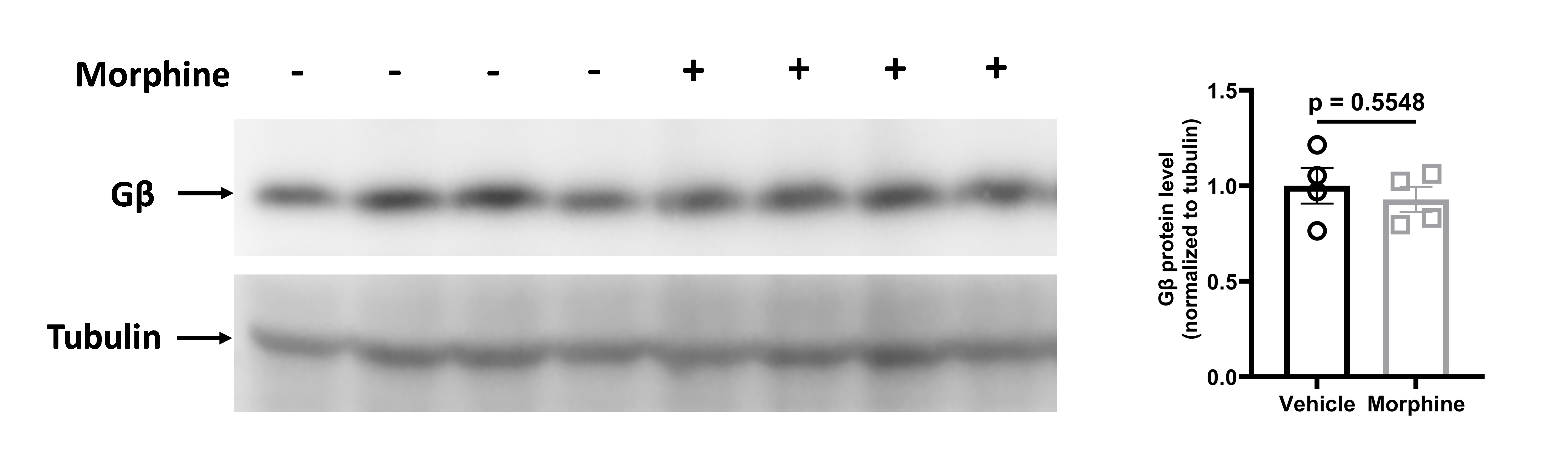

Supplement: S5 Fig — The mice received a single daily injection of morphine (i.p.) for 6 consecutive days with doses escalating at 10, 20, 30, 40, 50, and 50 mg/kg in their home cage to develop morphine dependence. Two hours after the last morphine injection, the PVT tissue was quickly dissected on ice and the Gβ expression was examined by WB using specific anti-Gβ antibody. The mean intensity of bands was quantified using Image J and normalized to their corresponding loading controls, and Student’s t test was applied, n = 4. (TIF) [file pbio.3002716.s005.tif]

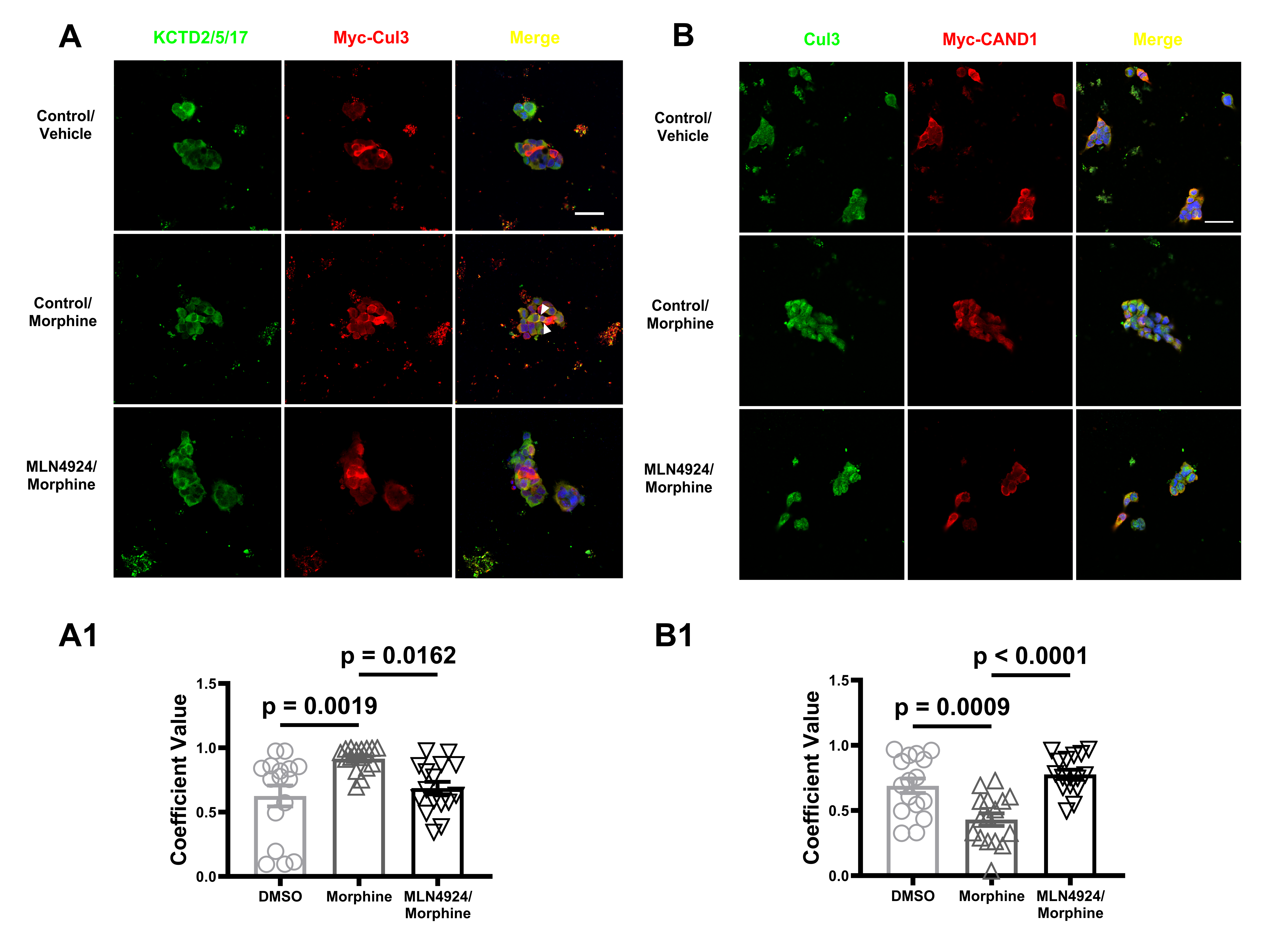

Supplement: S6 Fig — HEK-μR cells were transfected with Myc-Cul3 (A) or Myc-CAND1 (B) for 48 h. After transfection, the cells were pretreated with either 1 μm MLN4924 or vehicle for 30 min before being treated with 10 μm morphine or vehicle for another 2 h. Immunofluorescence double staining was performed to examine the co-localization of KCTD2/5/17 with Cul3 (A) and Cul3 with CAND1 (B) using anti-KCTD2/5/17, anti-Cul3, or anti-Myc antibodies. Images are representative of 3 independent experiments. The co-localization was quantified using Fiji software. Mander’s coefficient value was analyzed for Cul3 overlapping with KCTD2/5/17 (A1) and CAND1 overlapping with Cul3 (B1). One-way ANOVA followed by Tukey’s test. Mean ± SEM, n = 16 cells/3 independent experiments. Scale bar, 50 μm. The data underlying the graphs shown in the figure can be found in S1 Data. (TIF) [file pbio.3002716.s006.tif]

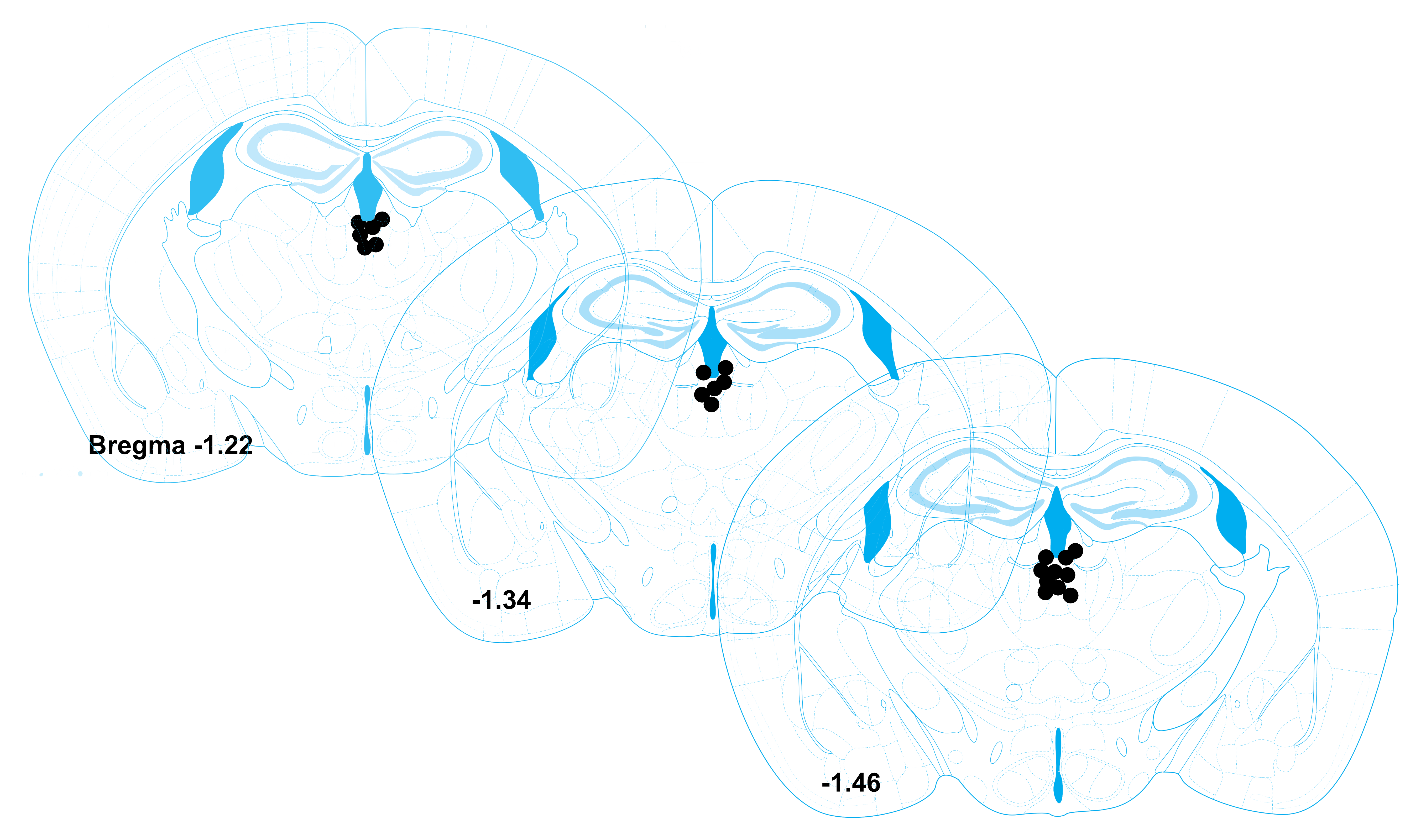

Supplement: S7 Fig — (TIF) [file pbio.3002716.s007.tif]
