# Supplementary material for: KCTD proteins regulate morphine dependence via heterologous sensitization of adenylyl cyclase 1 in mice
Source: PLoS Biol. 2024 Jul 15;22(7):e3002716. doi: 10.1371/journal.pbio.3002716 (PMC11271871; doi:10.1371/journal.pbio.3002716)
Supplement: S1 Raw Images — (PDF) [file pbio.3002716.s011.pdf]

Figure 1G

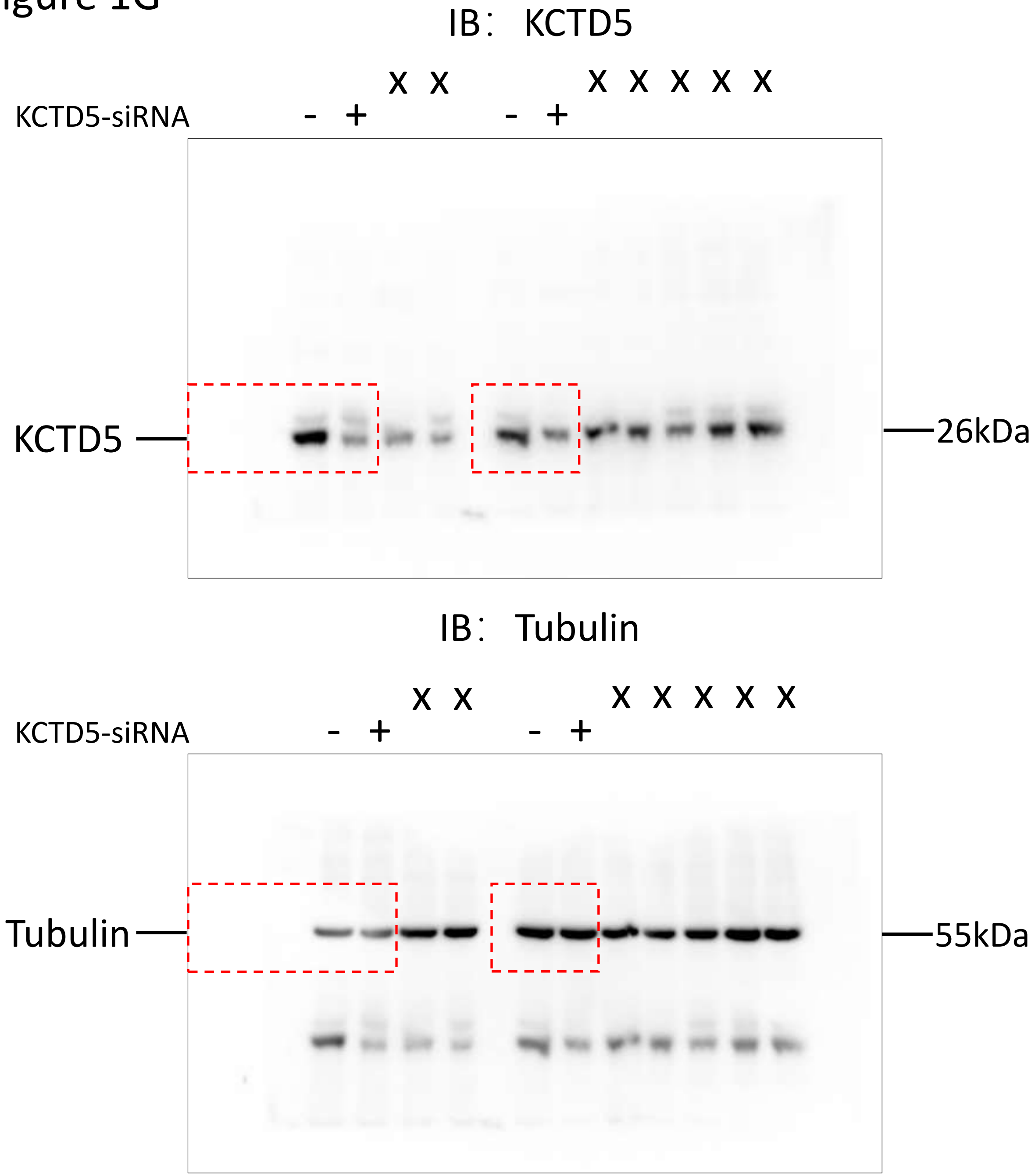

IB： KCTD5
x x
x x x x x
KCTD5-siRNA
- +
- +
KCTD5
26kDa
IB： Tubulin
x x
x x x x x
KCTD5-siRNA
- +
- +
Tubulin
55kDa

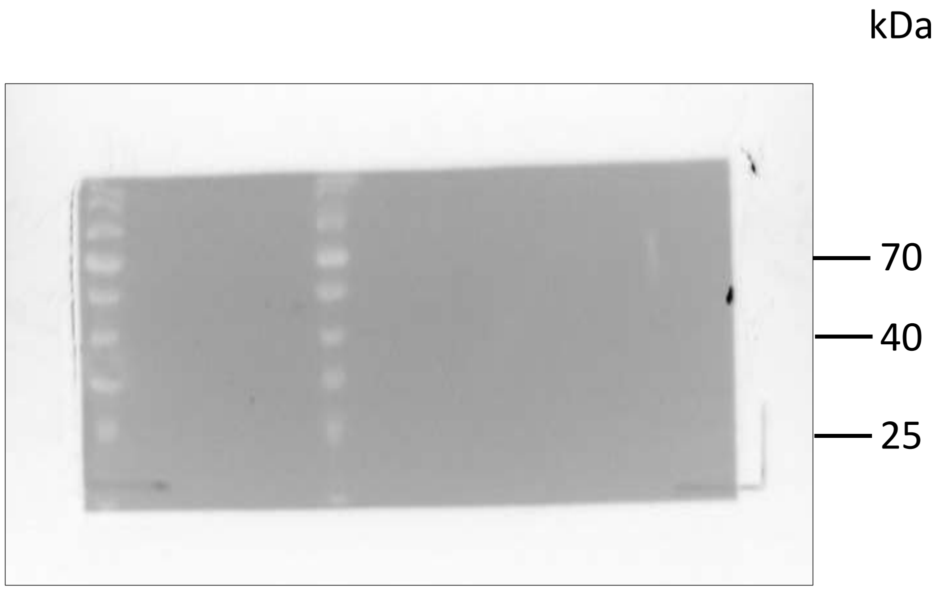

kDa
70
40
25

Figure 1G

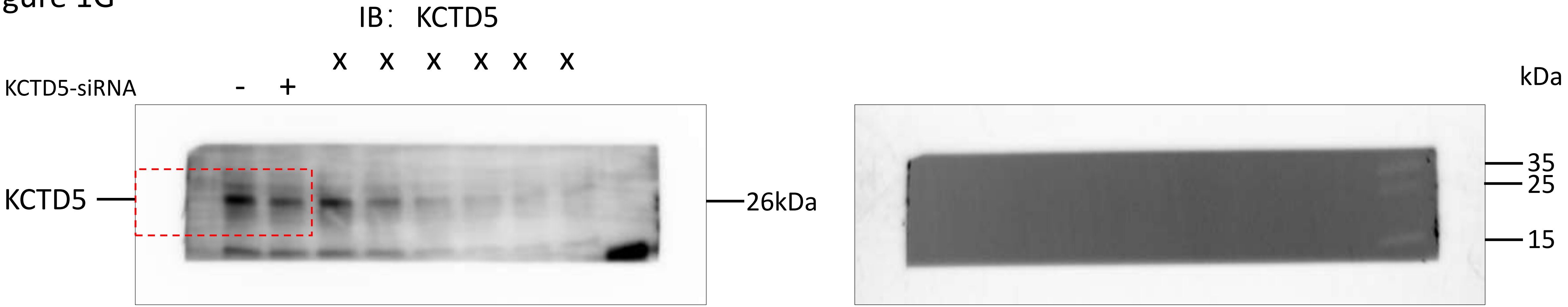
IB： KCTD5
X X X X X X
KCTD5-siRNA - +
kDa
KCTD5
26kDa
35
25
15

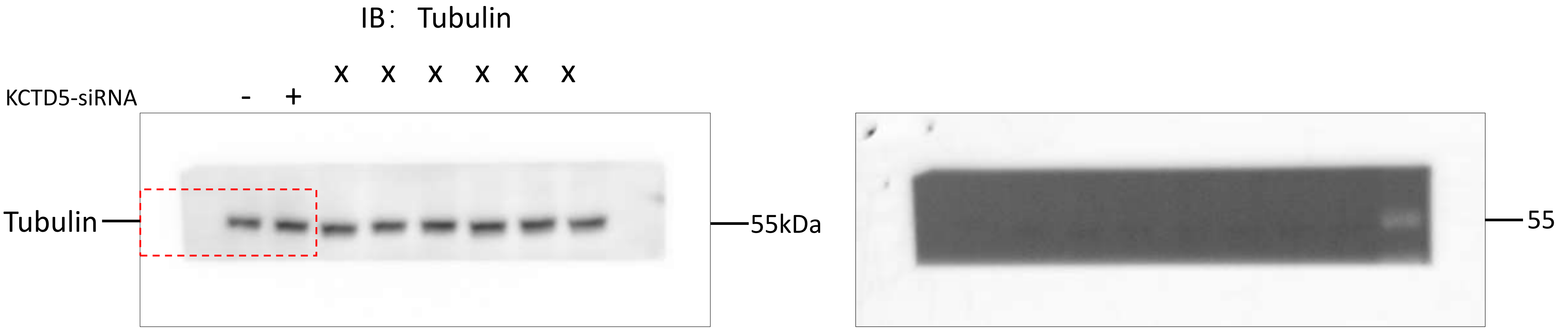
IB： Tubulin
X X X X X X
KCTD5-siRNA - +
Tubulin
55kDa
55

Figure 1H

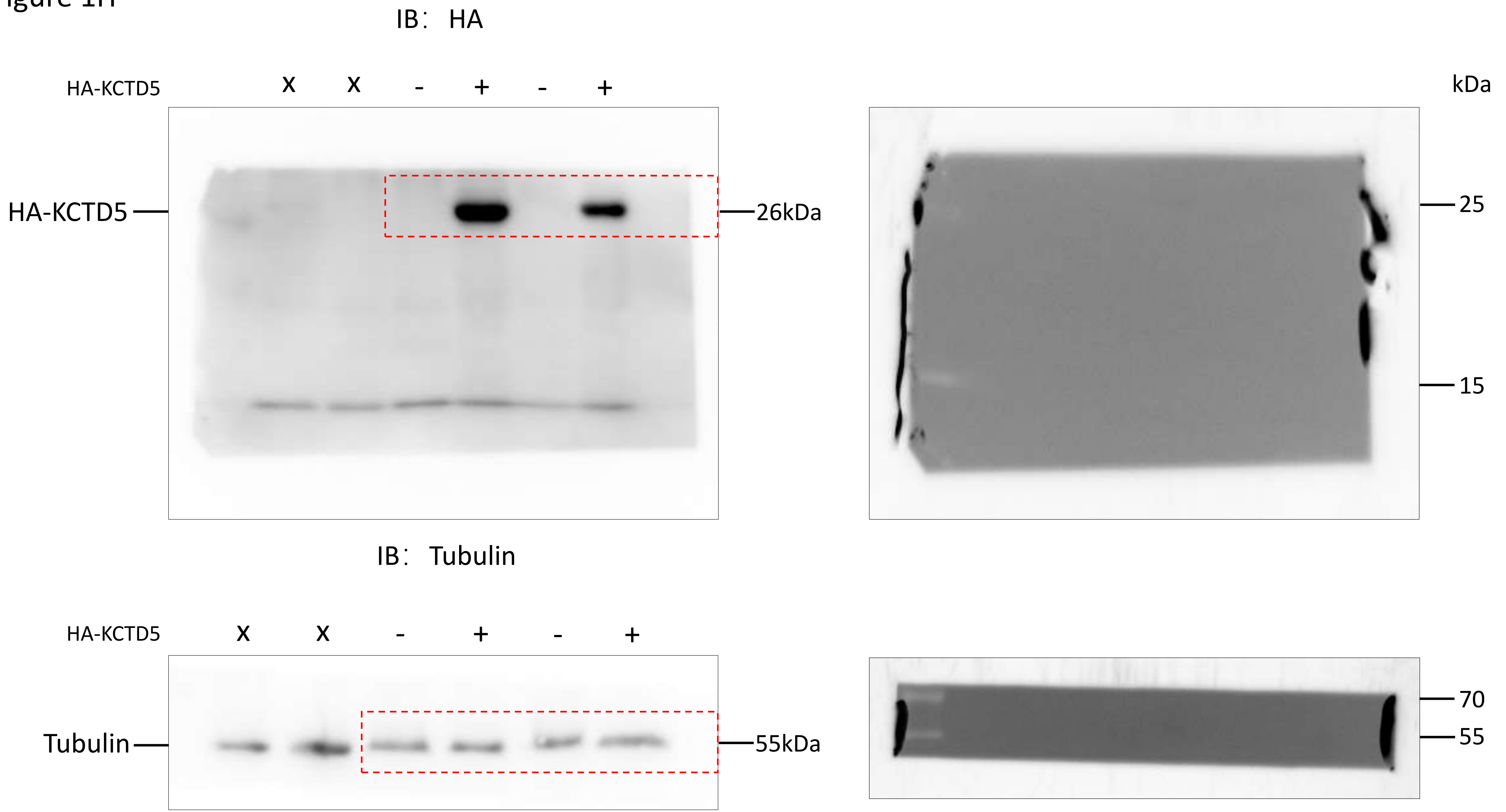
IB: HA
HA-KCTD5
x x - + - +
kDa
HA-KCTD5
26kDa
25
15
IB: Tubulin
HA-KCTD5
x x - + - +
Tubulin
55kDa
70
55

Figure 1H

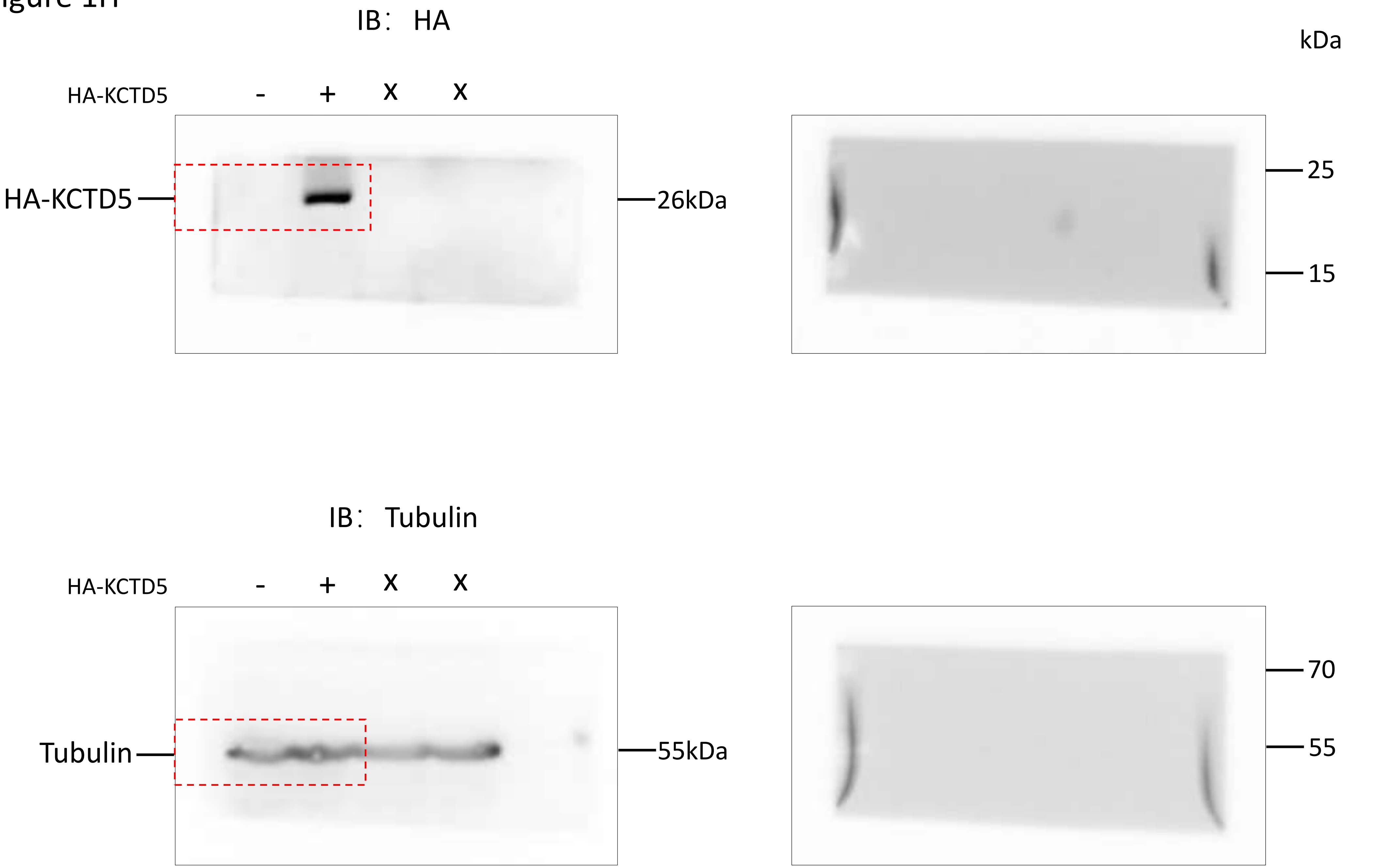
IB: HA
kDa
HA-KCTD5 - + X X
HA-KCTD5
26kDa
25
15
IB: Tubulin
HA-KCTD5 - + X X
Tubulin
55kDa
70
55

Figure 2A

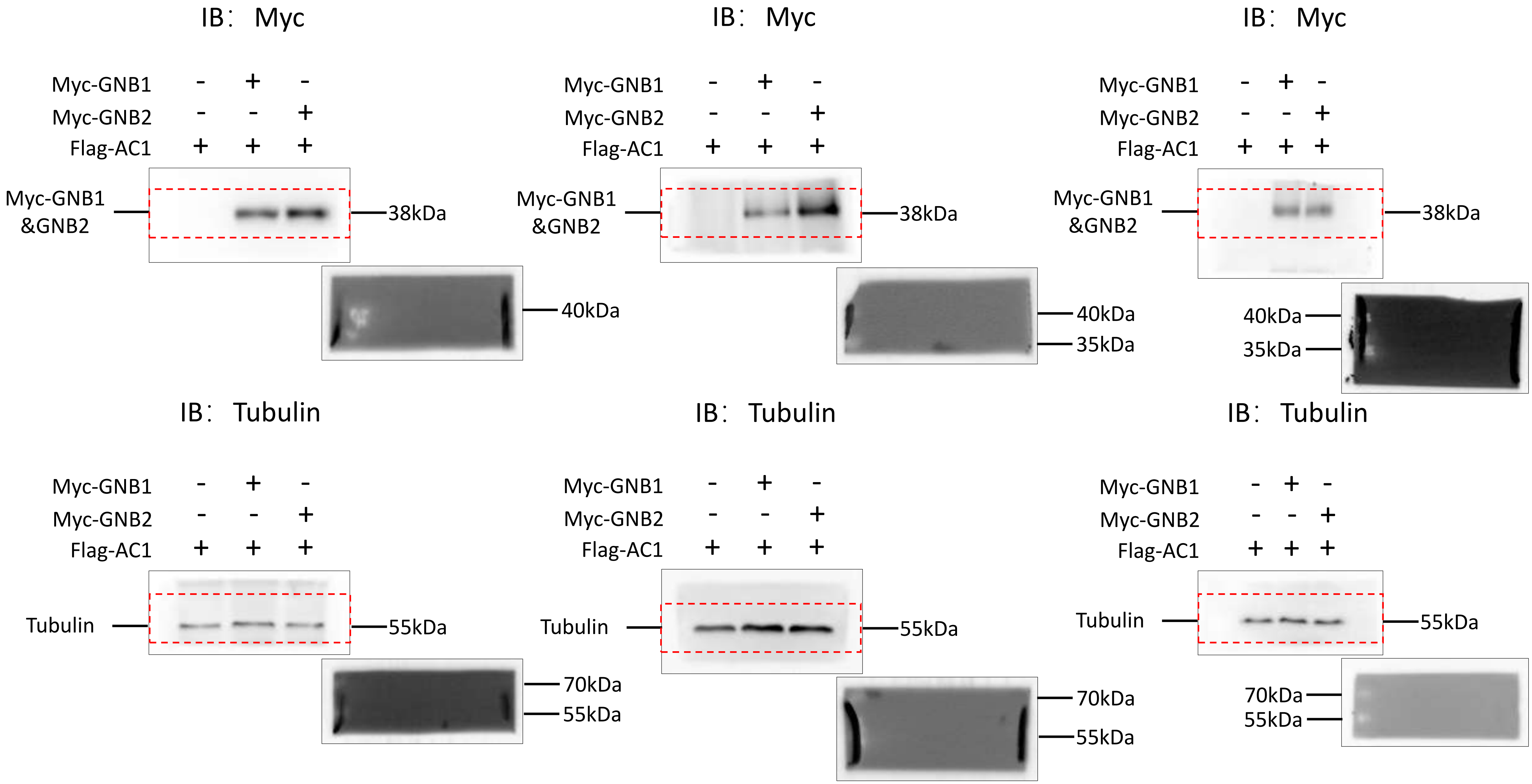
IB：Myc
Myc-GNB1 - + -
Myc-GNB2 - - +
Flag-AC1 + + +
Myc-GNB1 &GNB2
38kDa
40kDa
IB：Myc
Myc-GNB1 - + -
Myc-GNB2 - - +
Flag-AC1 + + +
Myc-GNB1 &GNB2
38kDa
40kDa
35kDa
IB：Myc
Myc-GNB1 - + -
Myc-GNB2 - - +
Flag-AC1 + + +
Myc-GNB1 &GNB2
38kDa
40kDa
35kDa
IB：Tubulin
Myc-GNB1 - + -
Myc-GNB2 - - +
Flag-AC1 + + +
Tubulin
55kDa
70kDa
55kDa
IB：Tubulin
Myc-GNB1 - + -
Myc-GNB2 - - +
Flag-AC1 + + +
Tubulin
55kDa
70kDa
55kDa
IB：Tubulin
Myc-GNB1 - + -
Myc-GNB2 - - +
Flag-AC1 + + +
Tubulin
55kDa
70kDa
55kDa

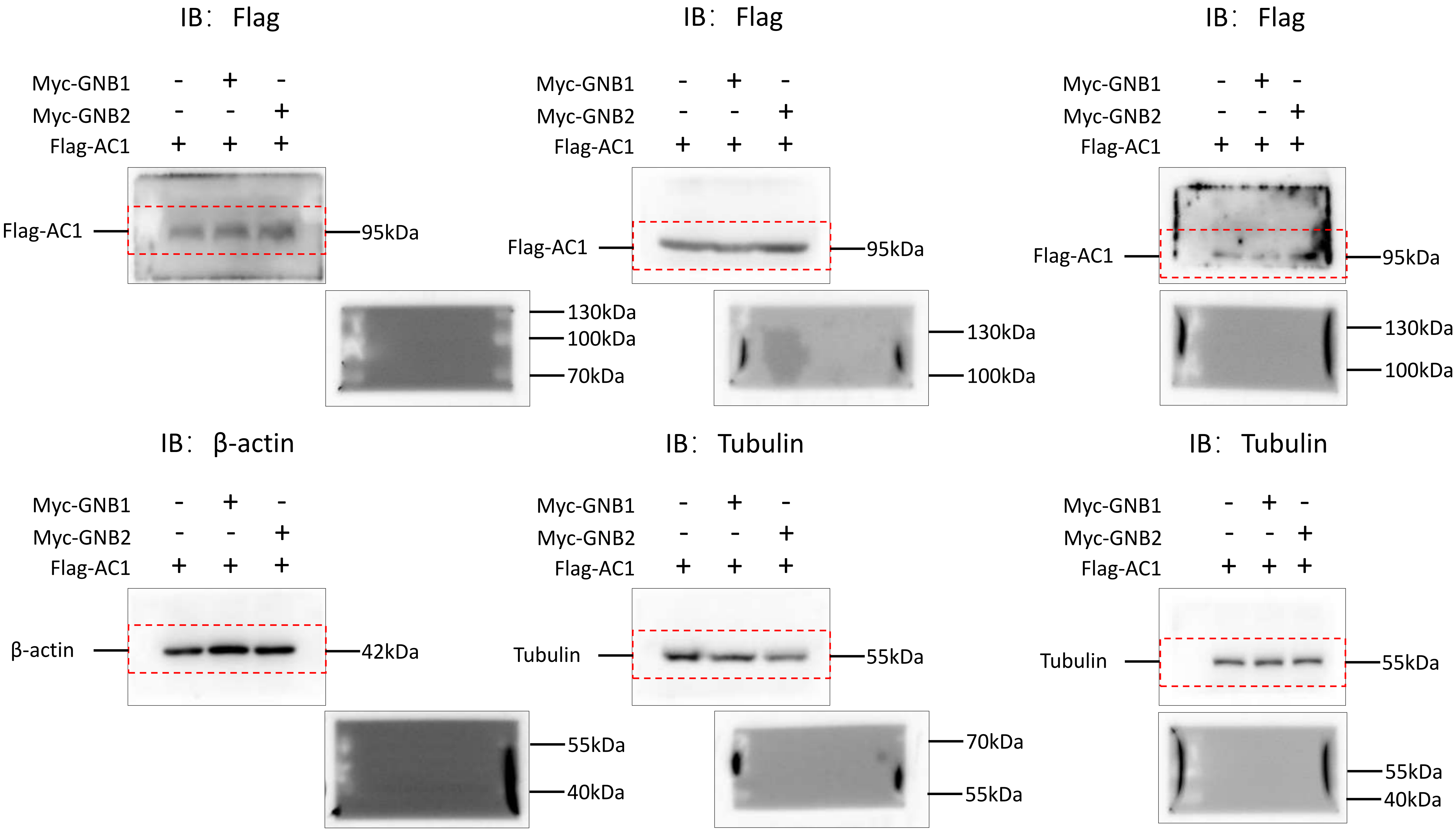
IB： Flag
Myc-GNB1 - + -
Myc-GNB2 - - +
Flag-AC1 + + +
Flag-AC1
95kDa
130kDa
100kDa
70kDa
IB： Flag
Myc-GNB1 - + -
Myc-GNB2 - - +
Flag-AC1 + + +
Flag-AC1
95kDa
130kDa
100kDa
IB： Flag
Myc-GNB1 - + -
Myc-GNB2 - - +
Flag-AC1 + + +
Flag-AC1
95kDa
130kDa
100kDa
IB： β-actin
Myc-GNB1 - + -
Myc-GNB2 - - +
Flag-AC1 + + +
β-actin
42kDa
55kDa
40kDa
IB： Tubulin
Myc-GNB1 - + -
Myc-GNB2 - - +
Flag-AC1 + + +
Tubulin
55kDa
70kDa
55kDa
IB： Tubulin
Myc-GNB1 - + -
Myc-GNB2 - - +
Flag-AC1 + + +
Tubulin
55kDa
55kDa
40kDa

Figure 2B

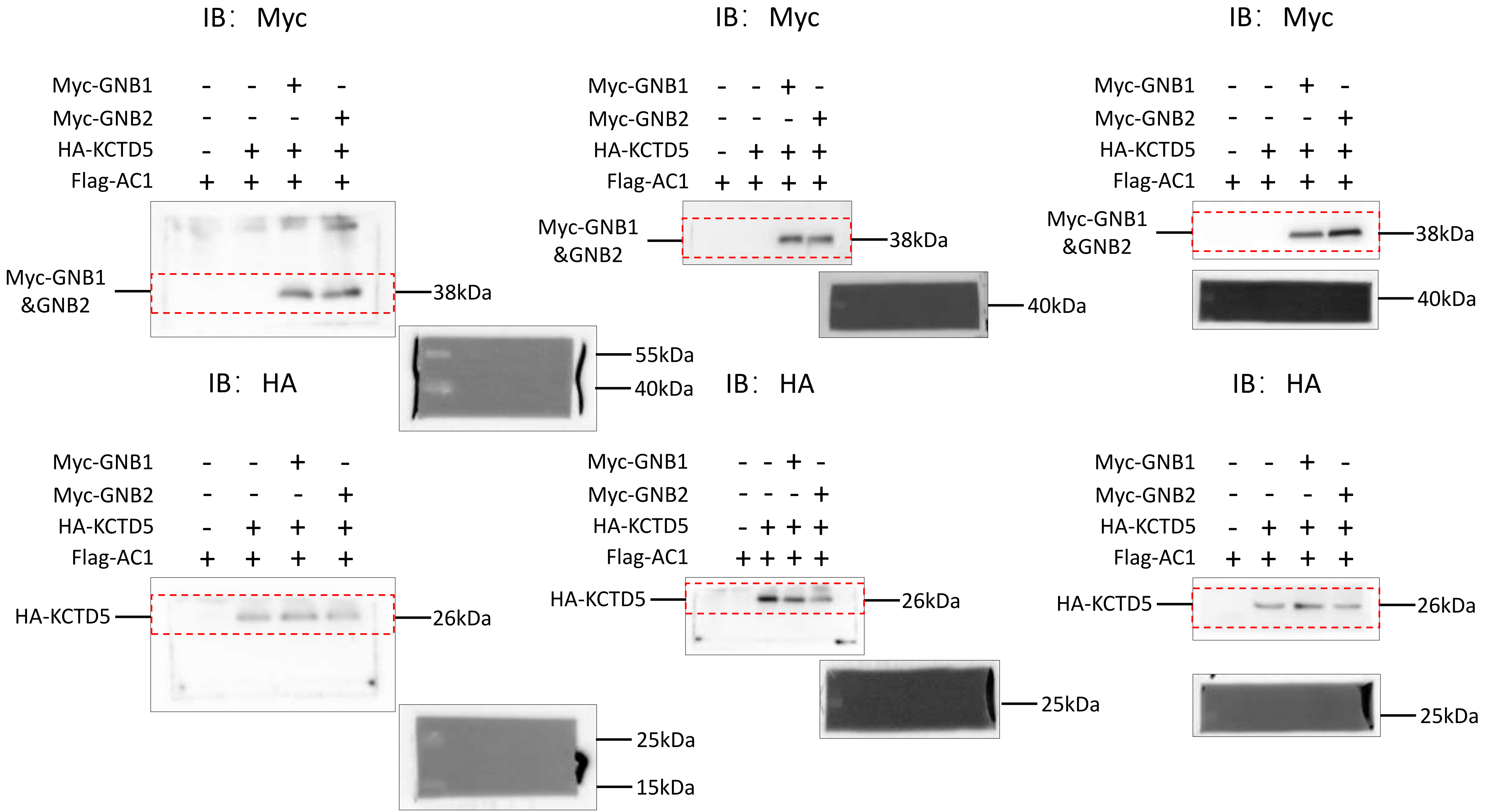

IB: Myc
Myc-GNB1 - - + -
Myc-GNB2 - - - +
HA-KCTD5 - + + +
Flag-AC1 + + + +
Myc-GNB1 &GNB2
38kDa
55kDa
40kDa
IB: HA
Myc-GNB1 - - + -
Myc-GNB2 - - - +
HA-KCTD5 - + + +
Flag-AC1 + + + +
HA-KCTD5
26kDa
25kDa
15kDa
IB: Myc
Myc-GNB1 &GNB2
38kDa
40kDa
IB: HA
HA-KCTD5
26kDa
25kDa
IB: Myc
Myc-GNB1 &GNB2
38kDa
40kDa
IB: HA
HA-KCTD5
26kDa
25kDa

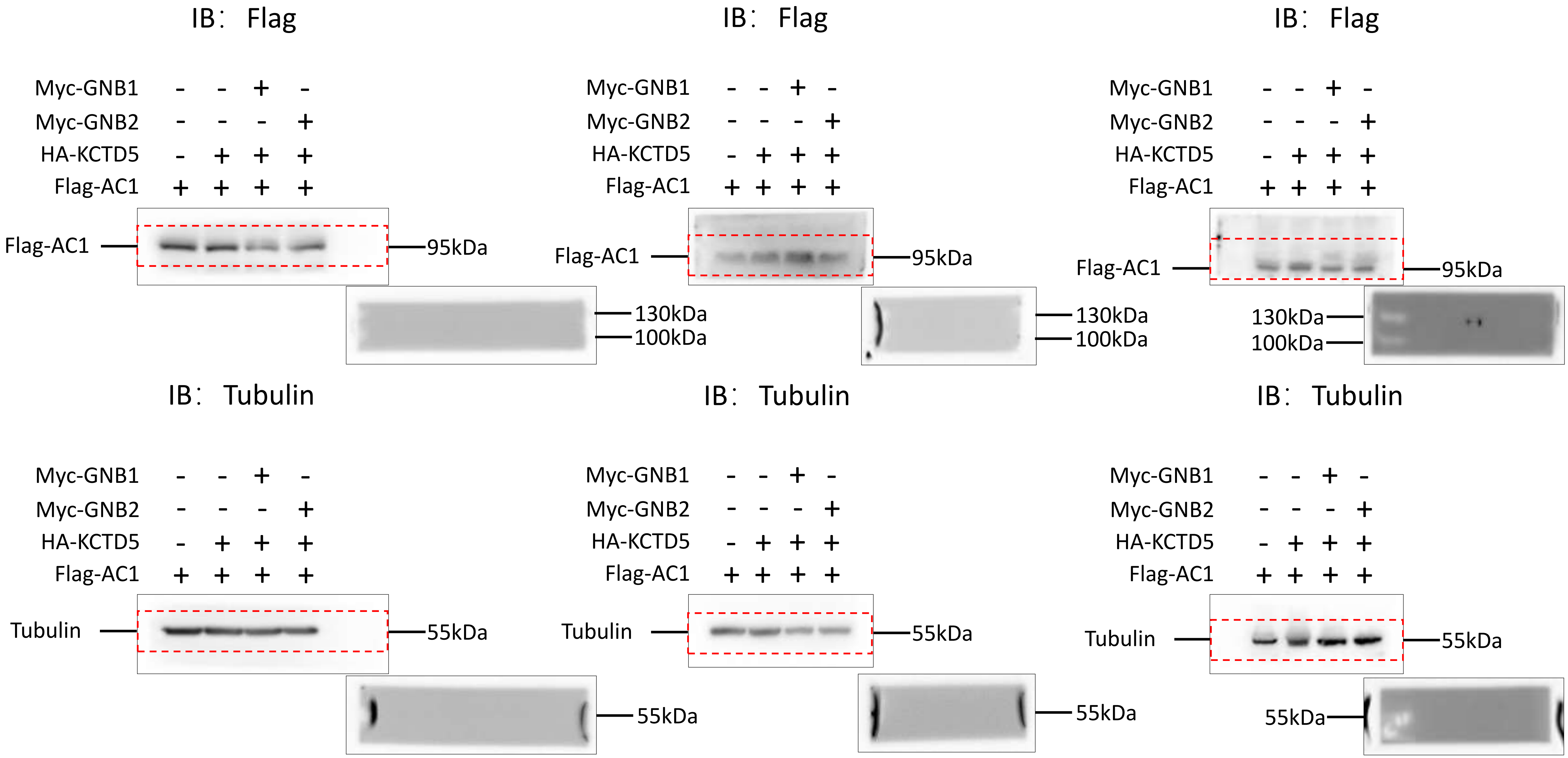
IB： Flag
Myc-GNB1 - - + -
Myc-GNB2 - - - +
HA-KCTD5 - + + +
Flag-AC1 + + + +
Flag-AC1
95kDa
130kDa
100kDa
IB： Flag
Myc-GNB1 - - + -
Myc-GNB2 - - - +
HA-KCTD5 - + + +
Flag-AC1 + + + +
Flag-AC1
95kDa
130kDa
100kDa
IB： Flag
Myc-GNB1 - - + -
Myc-GNB2 - - - +
HA-KCTD5 - + + +
Flag-AC1 + + + +
Flag-AC1
95kDa
130kDa
100kDa
IB： Tubulin
Myc-GNB1 - - + -
Myc-GNB2 - - - +
HA-KCTD5 - + + +
Flag-AC1 + + + +
Tubulin
55kDa
55kDa
IB： Tubulin
Myc-GNB1 - - + -
Myc-GNB2 - - - +
HA-KCTD5 - + + +
Flag-AC1 + + + +
Tubulin
55kDa
55kDa
IB： Tubulin
Myc-GNB1 - - + -
Myc-GNB2 - - - +
HA-KCTD5 - + + +
Flag-AC1 + + + +
Tubulin
55kDa
55kDa

Figure 2C

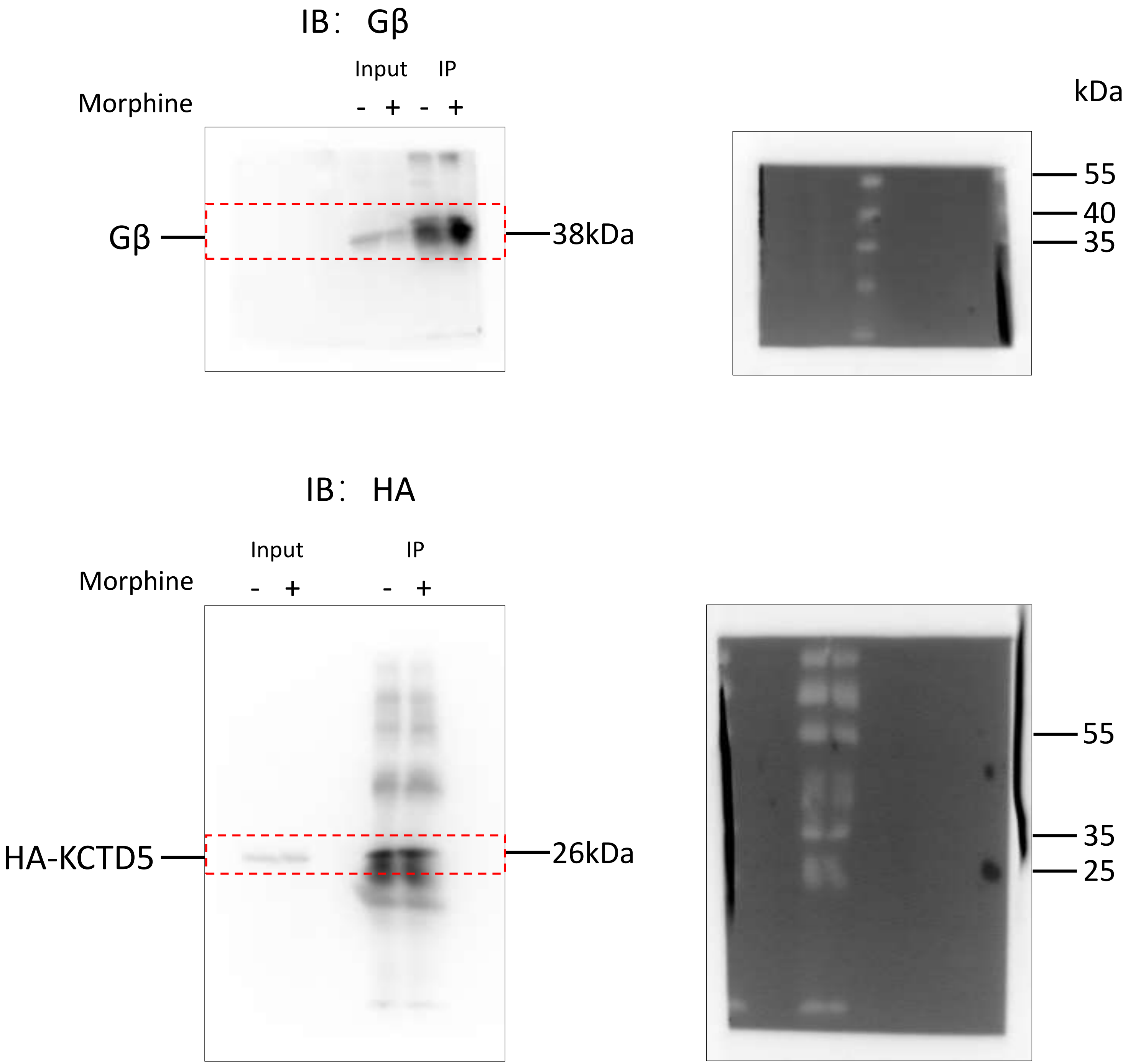
IB: Gβ
Input IP
Morphine - + - +
Gβ
38kDa
kDa
55
40
35
IB: HA
Input IP
Morphine - + - +
HA-KCTD5
26kDa
55
35
25

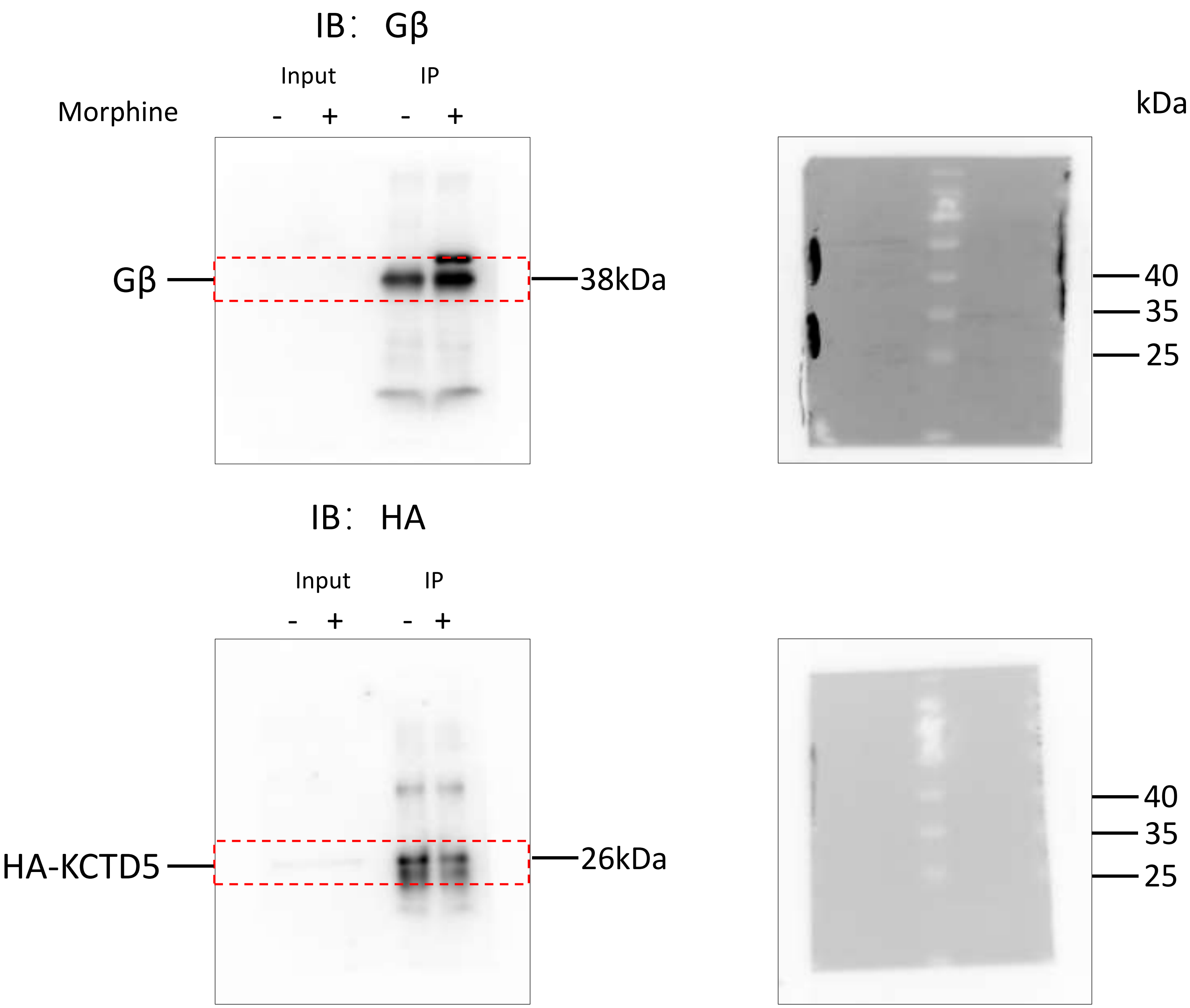

IB： Gβ
Input
IP
Morphine
-
+
-
+
Gβ
38kDa
kDa
40
35
25
IB： HA
Input
IP
-
+
-
+
HA-KCTD5
26kDa
40
35
25

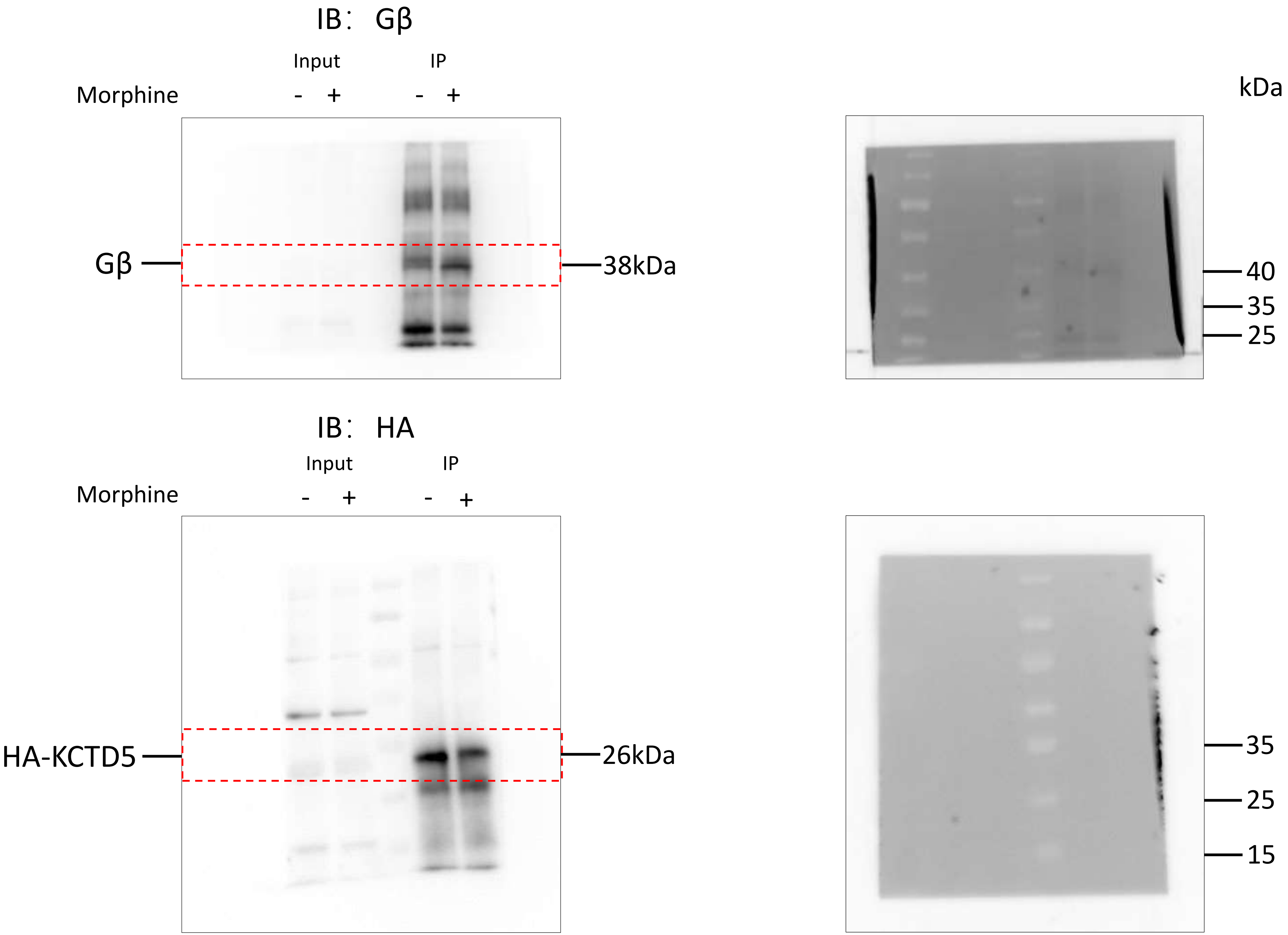
IB： Gβ
Input
IP
Morphine
-
+
-
+
Gβ
38kDa
kDa
40
35
25
IB： HA
Input
IP
Morphine
-
+
-
+
HA-KCTD5
26kDa
35
25
15

Figure 2D

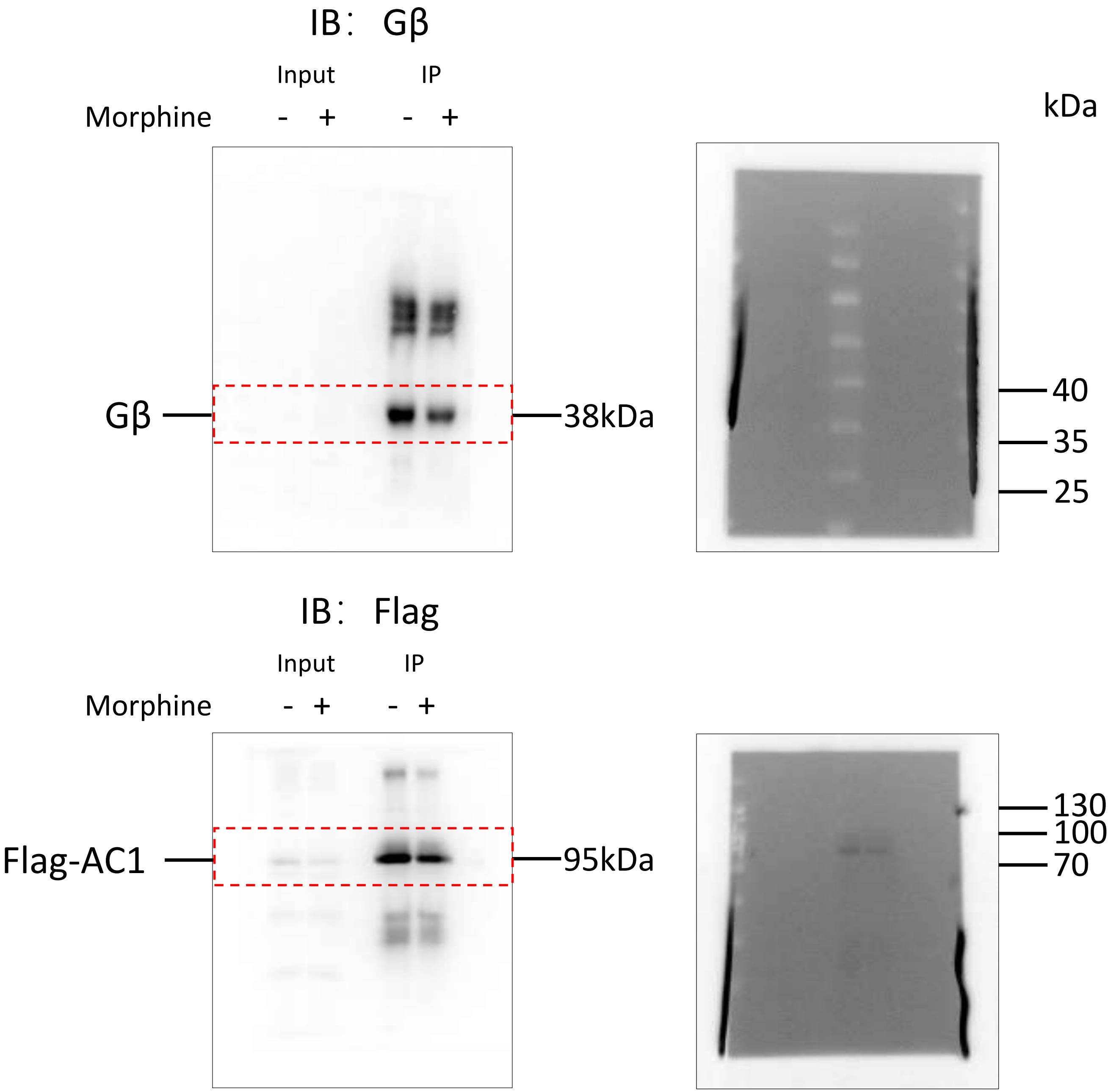
IB: Gβ
Input
IP
Morphine
- + - +
kDa
Gβ
38kDa
40
35
25
IB: Flag
Input
IP
Morphine
- + - +
Flag-AC1
95kDa
130
100
70

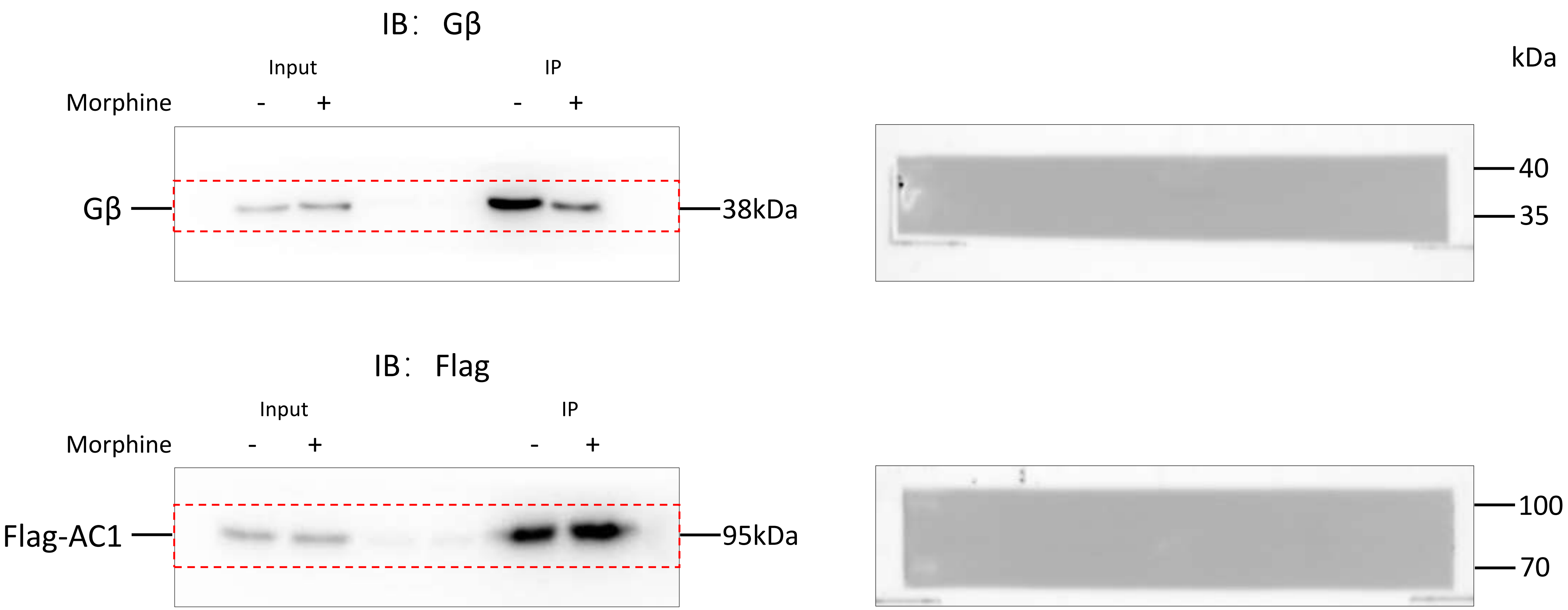
IB: Gβ
Input
IP
Morphine
-
+
-
+
Gβ
38kDa
kDa
40
35
IB: Flag
Input
IP
Morphine
-
+
-
+
Flag-AC1
95kDa
100
70

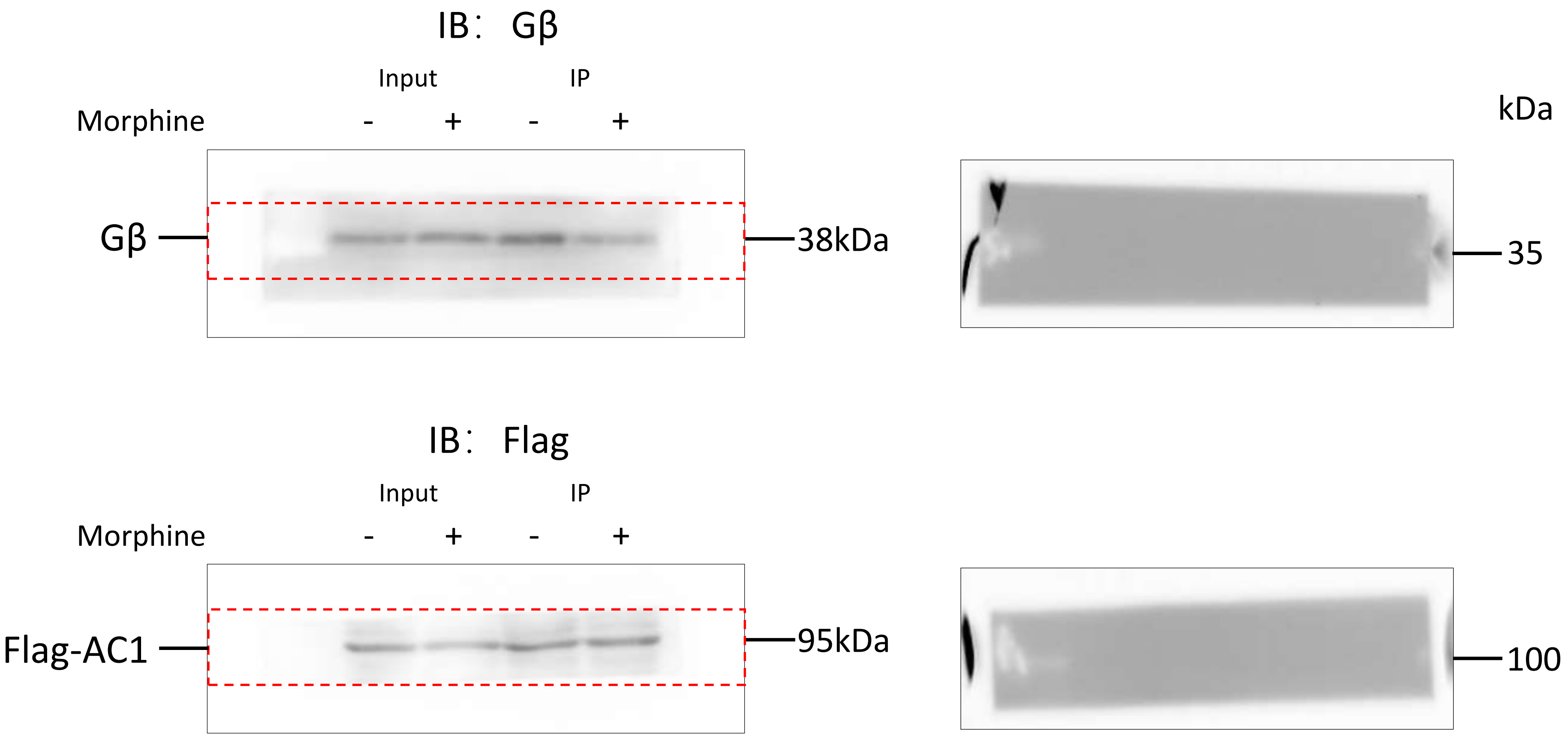

IB： Gβ
Input
IP
Morphine
- + - +
Gβ
38kDa
kDa
35
IB： Flag
Input
IP
Morphine
- + - +
Flag-AC1
95kDa
100

Figure 2E

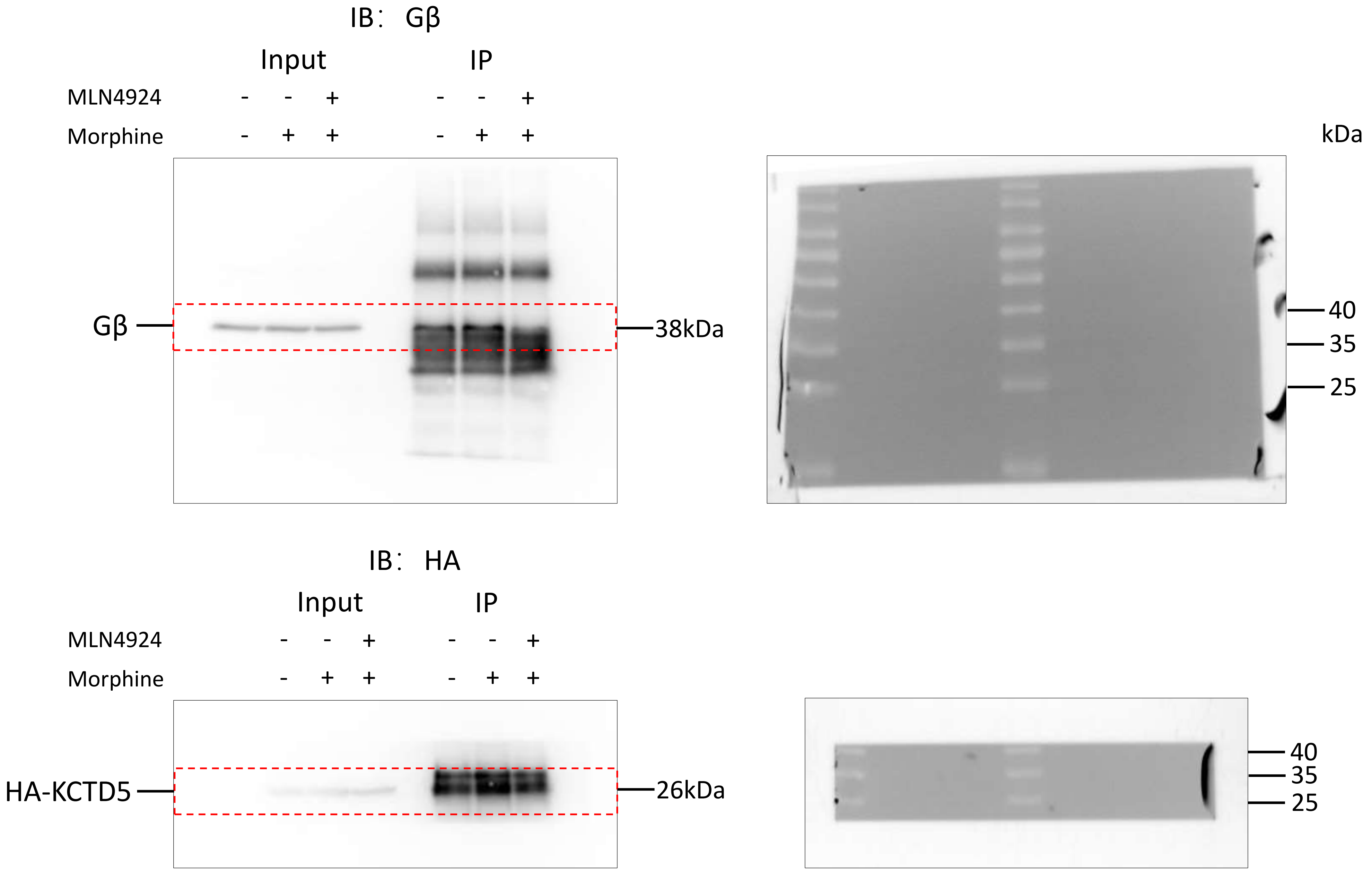
IB: Gβ
Input
IP
MLN4924 - - + - - +
Morphine - + + - + +
kDa
Gβ
38kDa
40
35
25
IB: HA
Input
IP
MLN4924 - - + - - +
Morphine - + + - + +
HA-KCTD5
26kDa
40
35
25

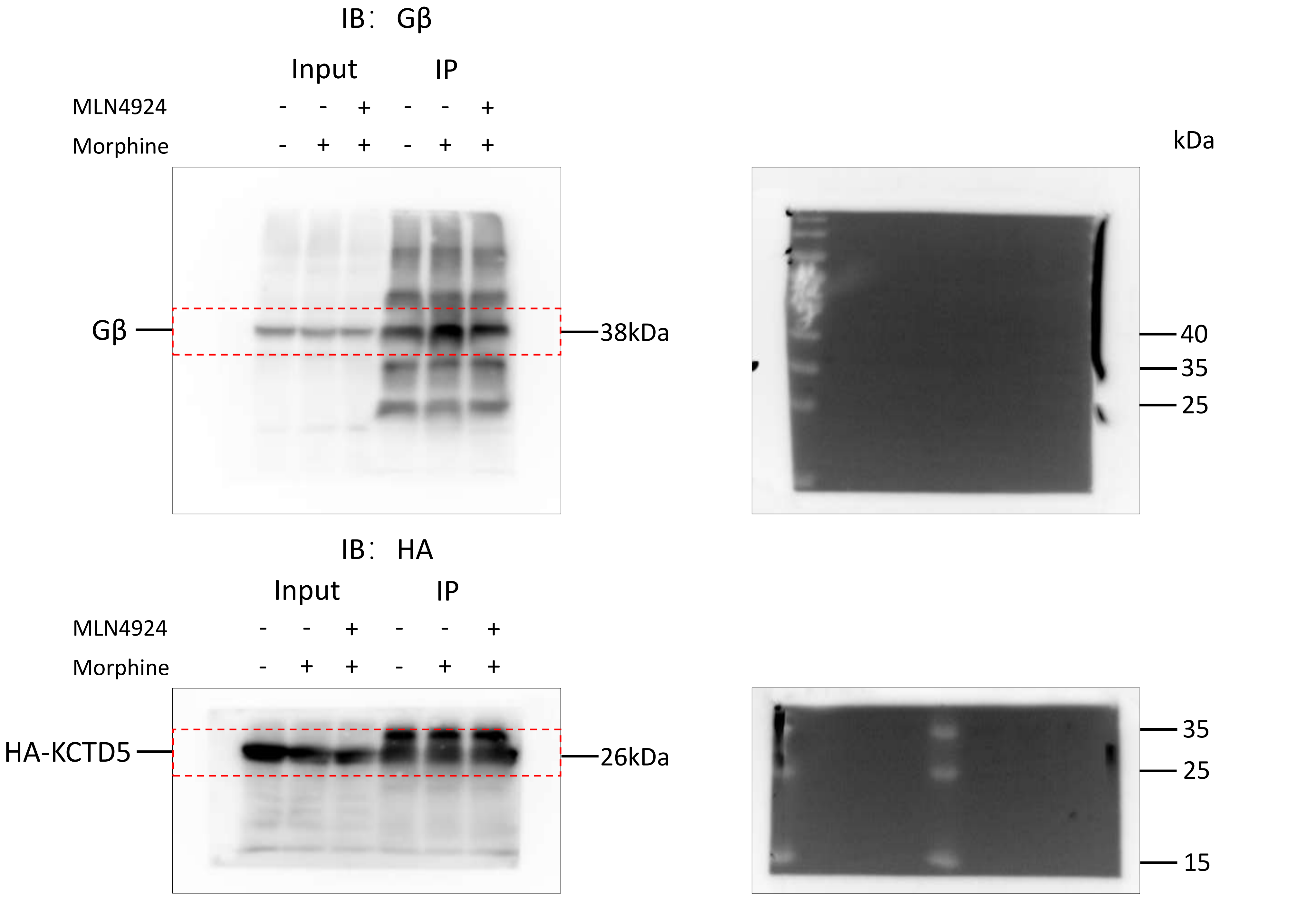

IB： Gβ
Input
IP
MLN4924 - - + - - +
Morphine - + + - + +
Gβ
38kDa
kDa
40
35
25
IB： HA
Input
IP
MLN4924 - - + - - +
Morphine - + + - + +
HA-KCTD5
26kDa
35
25
15

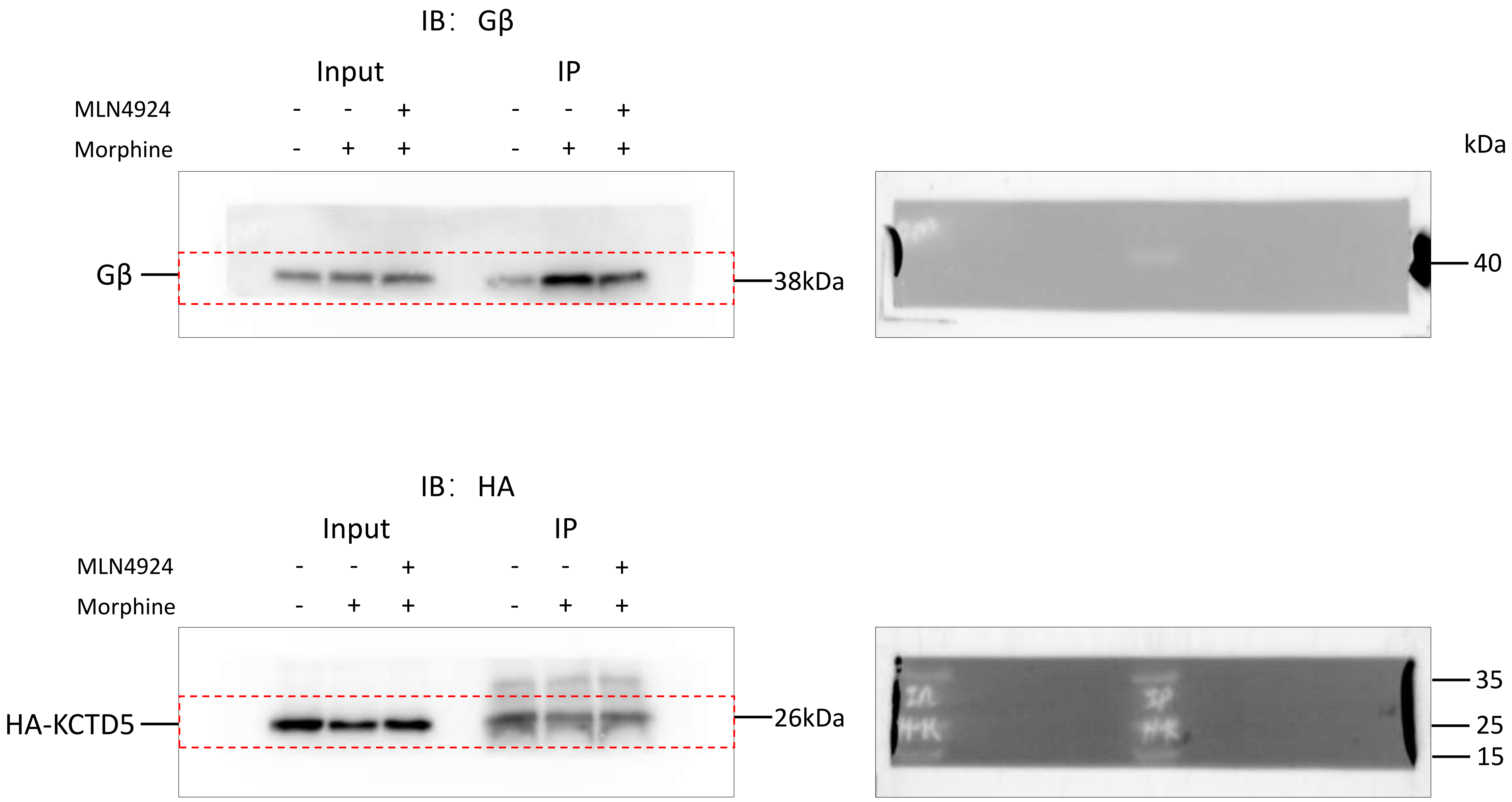

IB: Gβ
Input
IP
MLN4924 - - + - - +
Morphine - + + - + +
Gβ
38kDa
kDa
40
IB: HA
Input
IP
MLN4924 - - + - - +
Morphine - + + - + +
HA-KCTD5
26kDa
35
25
15

Figure 2F

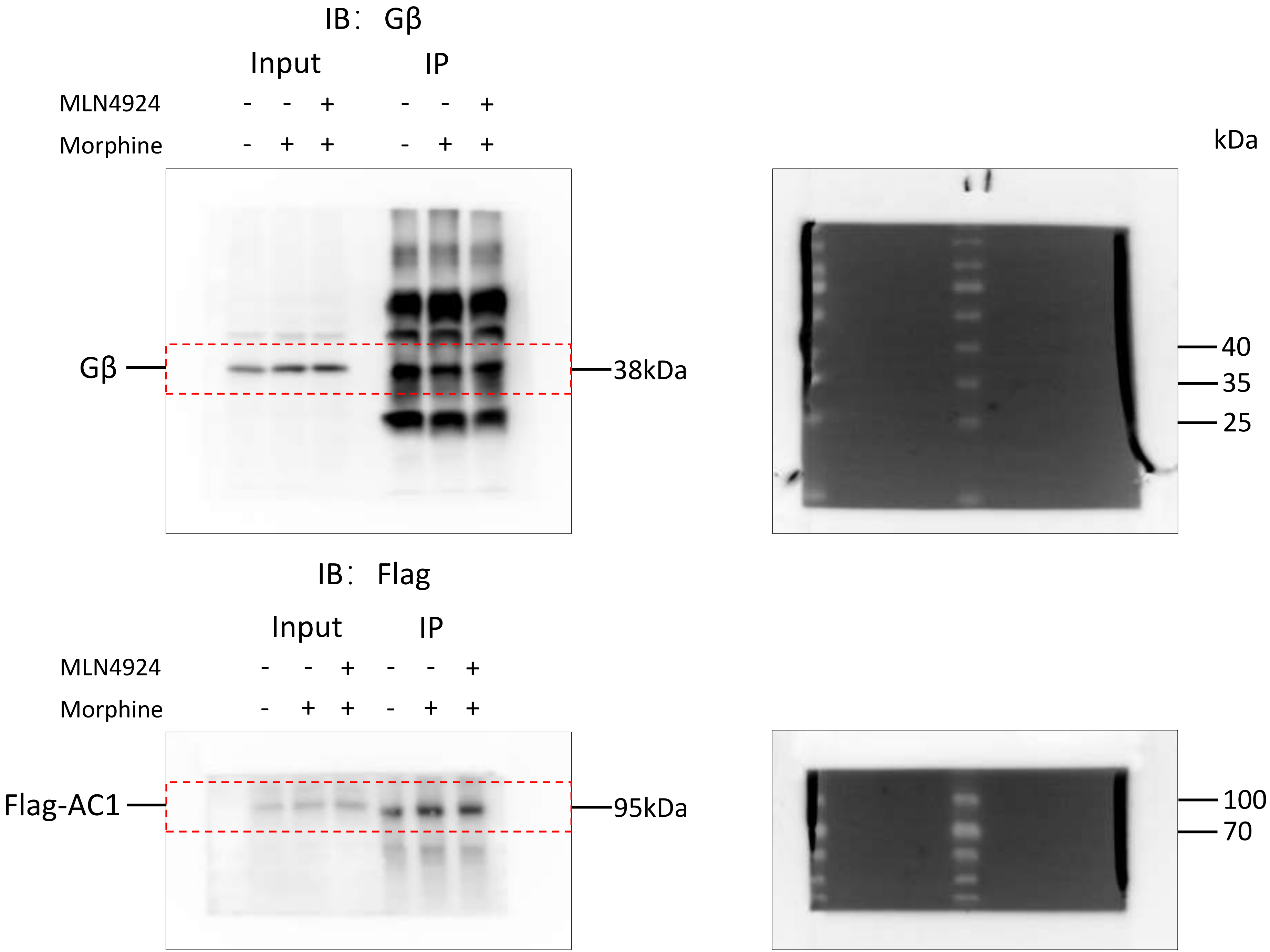
IB: Gβ
Input
IP
MLN4924 - - + - - +
Morphine - + + - + +
Gβ
38kDa
kDa
40
35
25
IB: Flag
Input
IP
MLN4924 - - + - - +
Morphine - + + - + +
Flag-AC1
95kDa
100
70

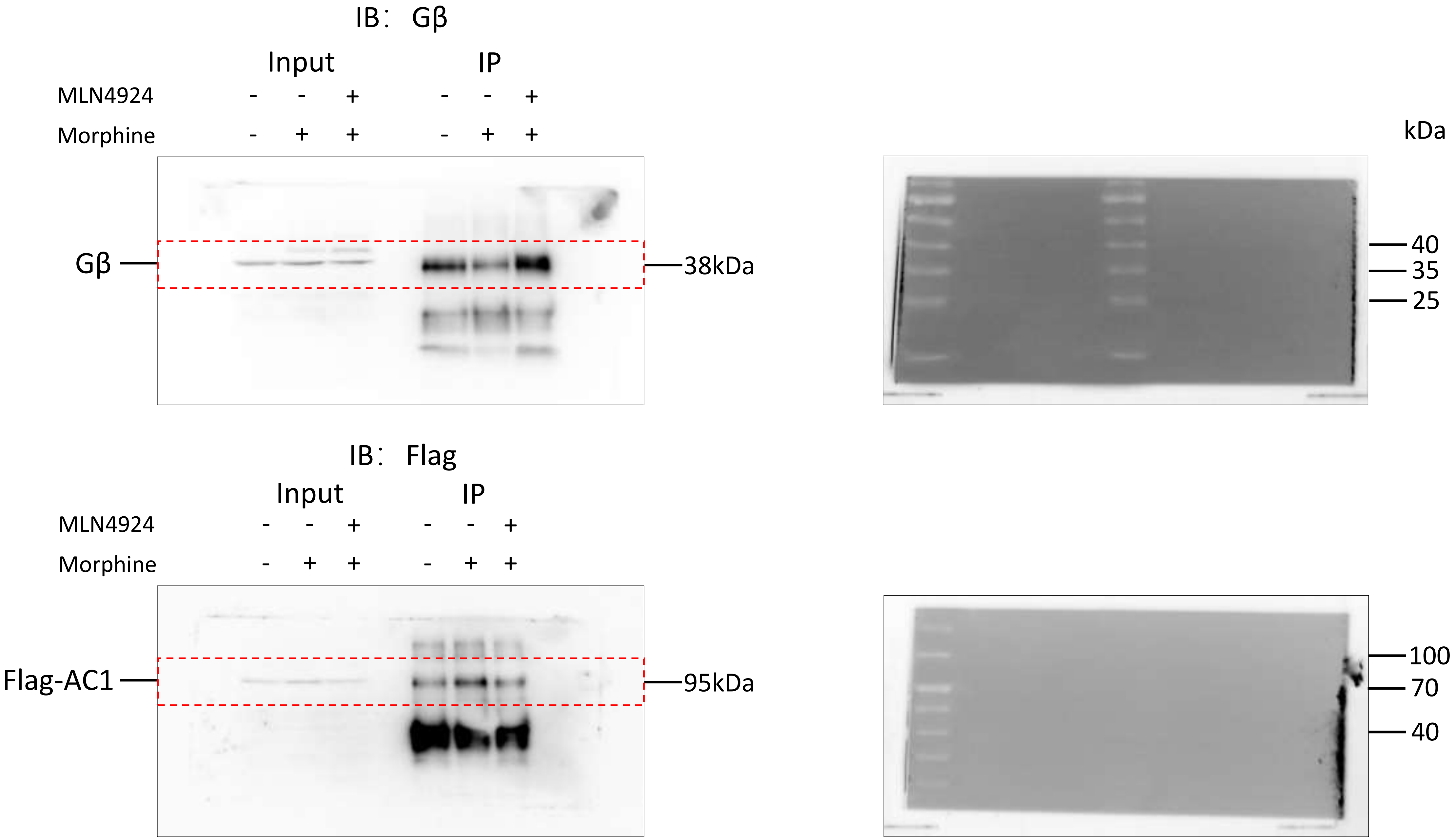

IB: Gβ
Input
IP
MLN4924
- - + - - +
Morphine
- + + - + +
Gβ
38kDa
kDa
40
35
25
IB: Flag
Input
IP
MLN4924
- - + - - +
Morphine
- + + - + +
Flag-AC1
95kDa
100
70
40

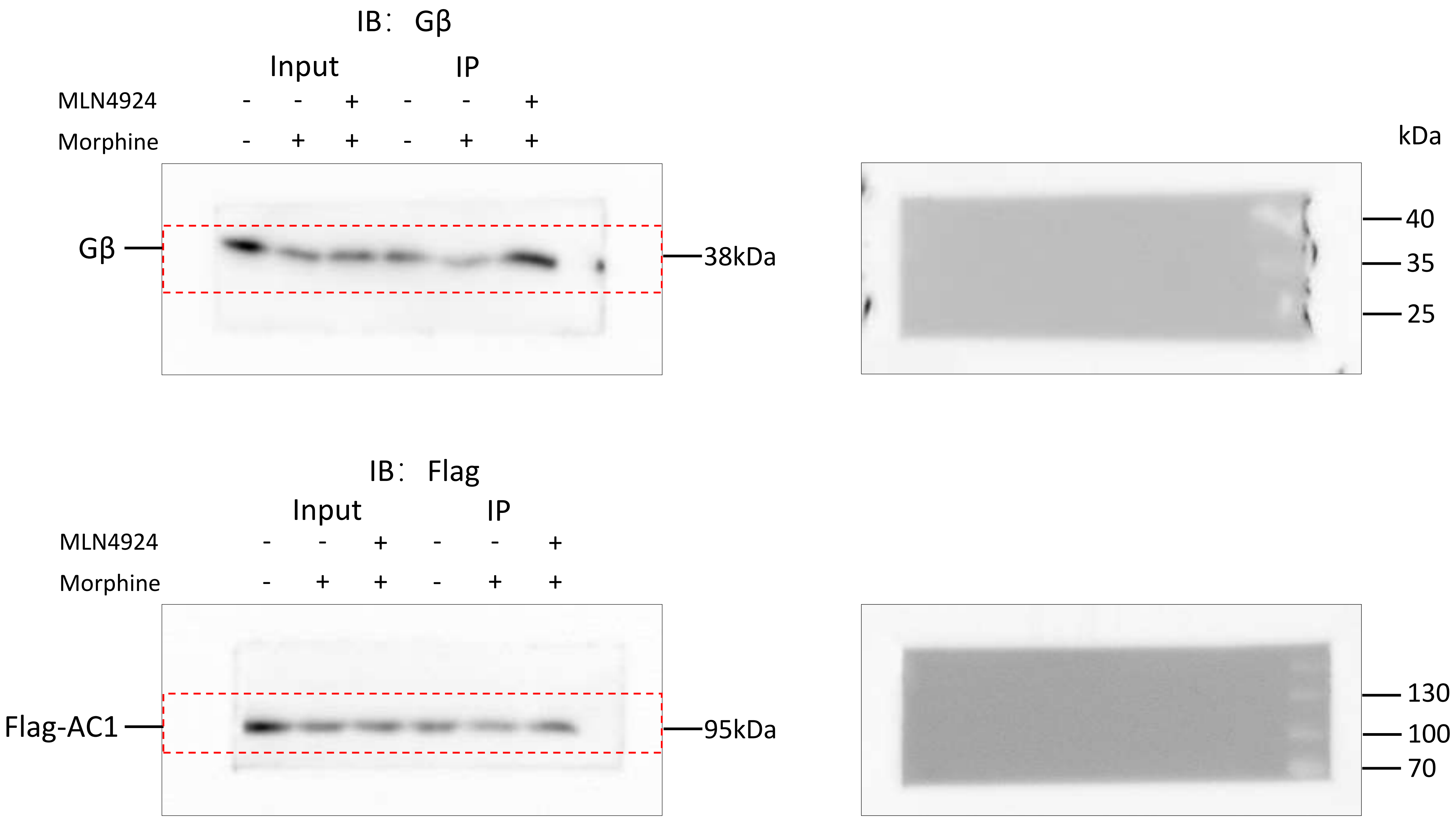
IB: Gβ
Input
IP
MLN4924 - - + - - +
Morphine - + + - + +
Gβ
38kDa
kDa
40
35
25
IB: Flag
Input
IP
MLN4924 - - + - - +
Morphine - + + - + +
Flag-AC1
95kDa
130
100
70

Figure 2G

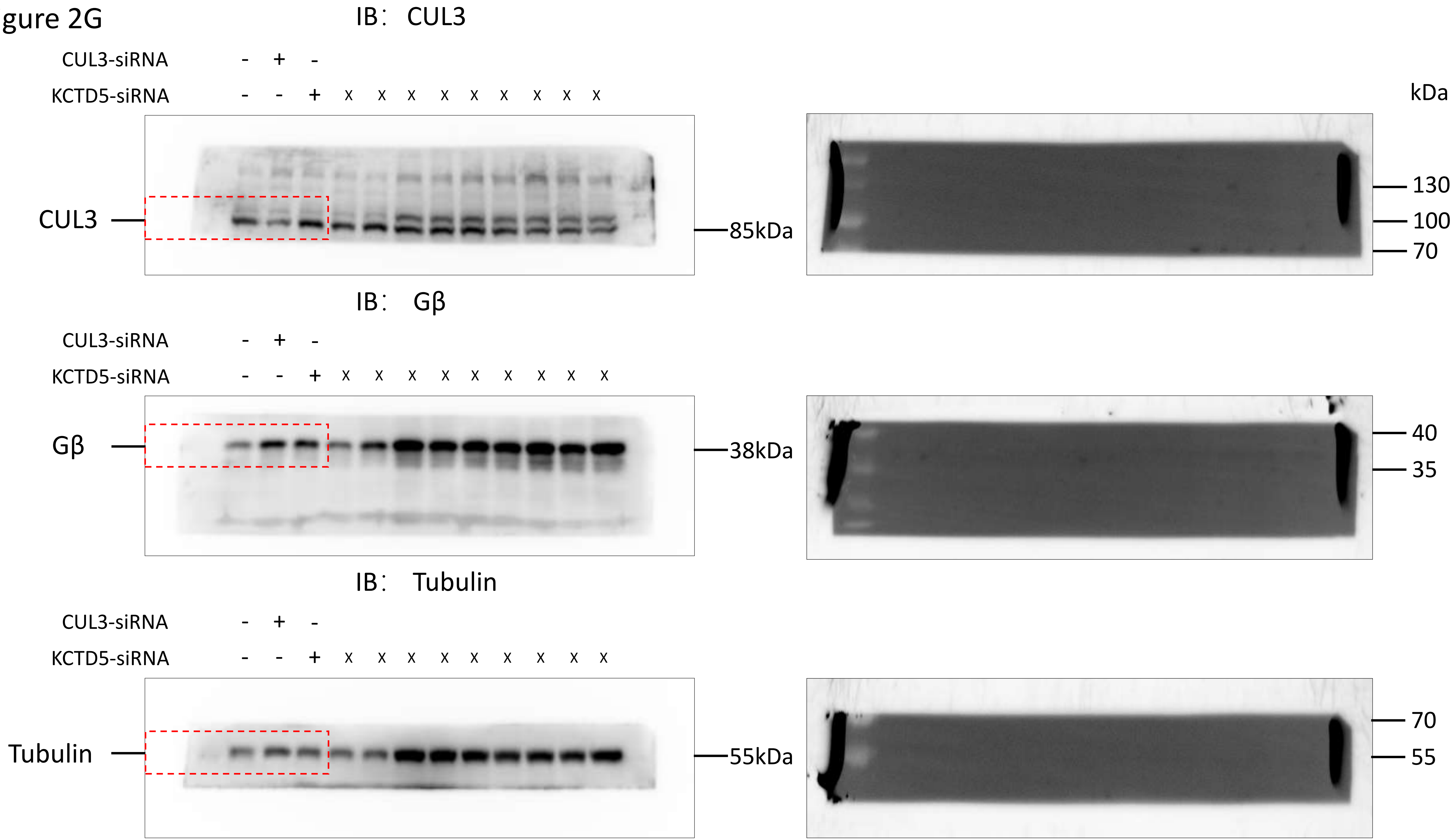
IB: CUL3
CUL3-siRNA - + -
KCTD5-siRNA - - + x x x x x x x x x
kDa
CUL3
85kDa
130
100
70
IB: Gβ
CUL3-siRNA - + -
KCTD5-siRNA - - + x x x x x x x x x
Gβ
38kDa
40
35
IB: Tubulin
CUL3-siRNA - + -
KCTD5-siRNA - - + x x x x x x x x x
Tubulin
55kDa
70
55

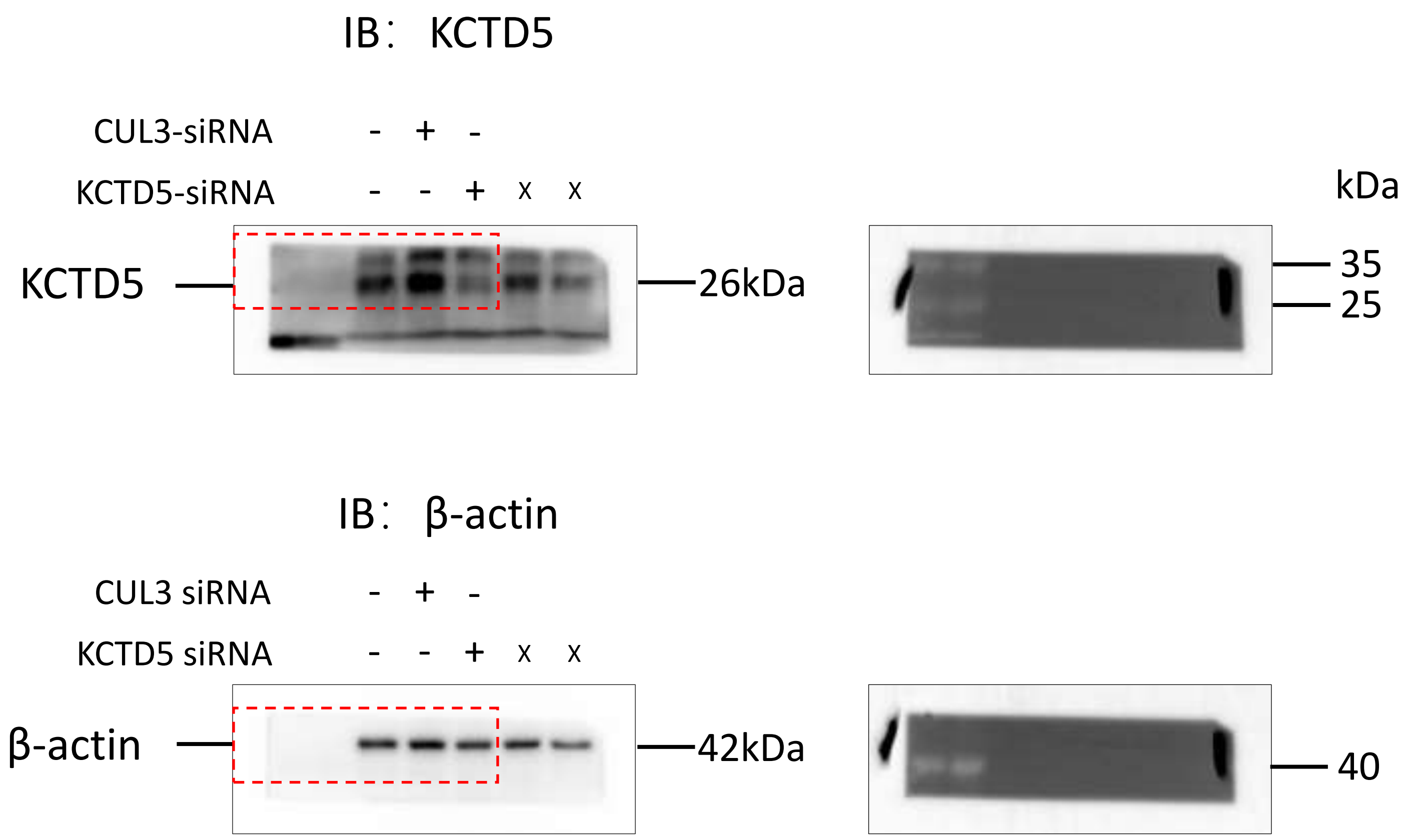
IB: KCTD5
CUL3-siRNA - + -
KCTD5-siRNA - - + x x
kDa
KCTD5
26kDa
35
25
IB: β-actin
CUL3 siRNA - + -
KCTD5 siRNA - - + x x
β-actin
42kDa
40

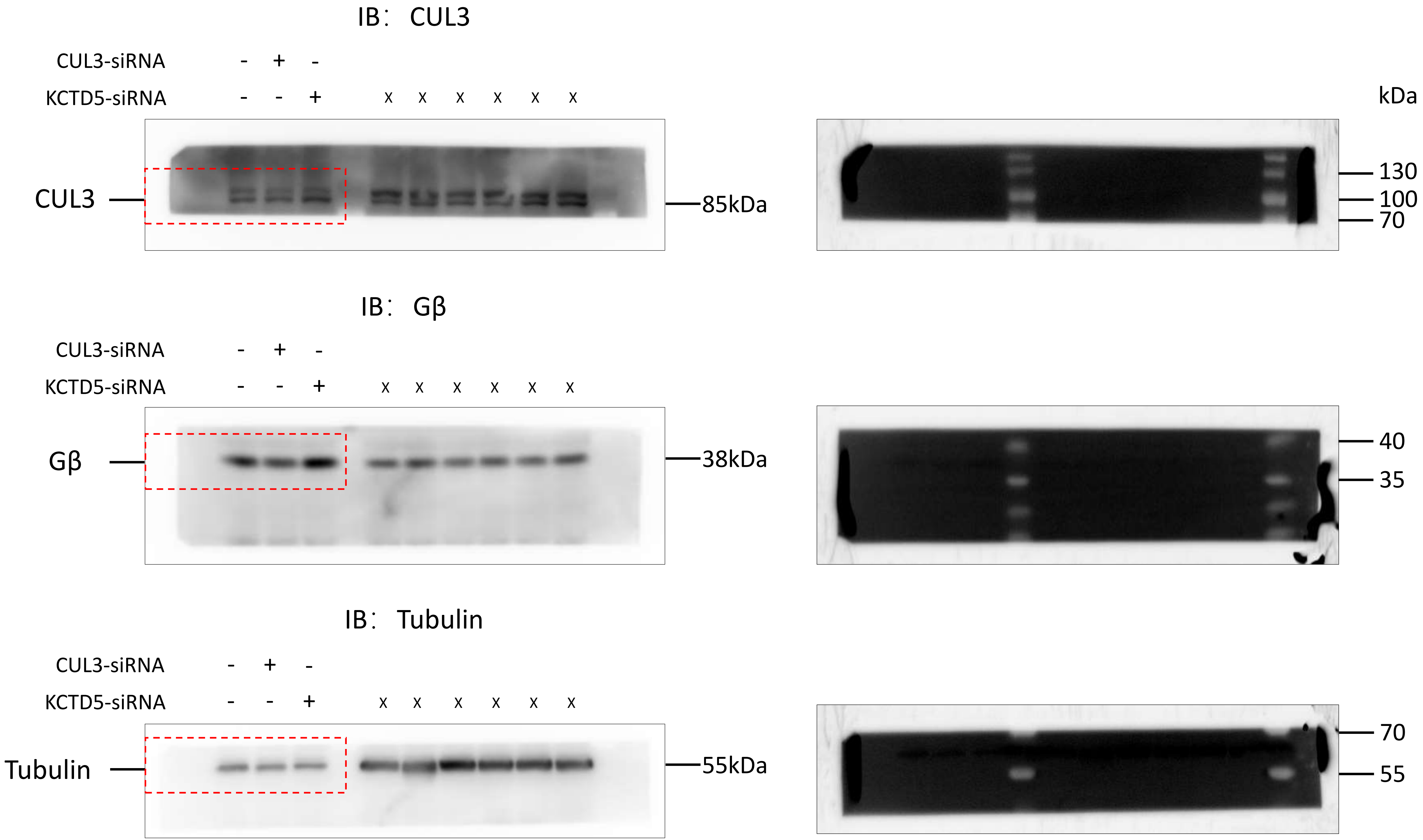
IB: CUL3
CUL3-siRNA - + -
KCTD5-siRNA - - + x x x x x x
kDa
CUL3
85kDa
130
100
70
IB: Gβ
CUL3-siRNA - + -
KCTD5-siRNA - - + x x x x x x
Gβ
38kDa
40
35
IB: Tubulin
CUL3-siRNA - + -
KCTD5-siRNA - - + x x x x x x
Tubulin
55kDa
70
55

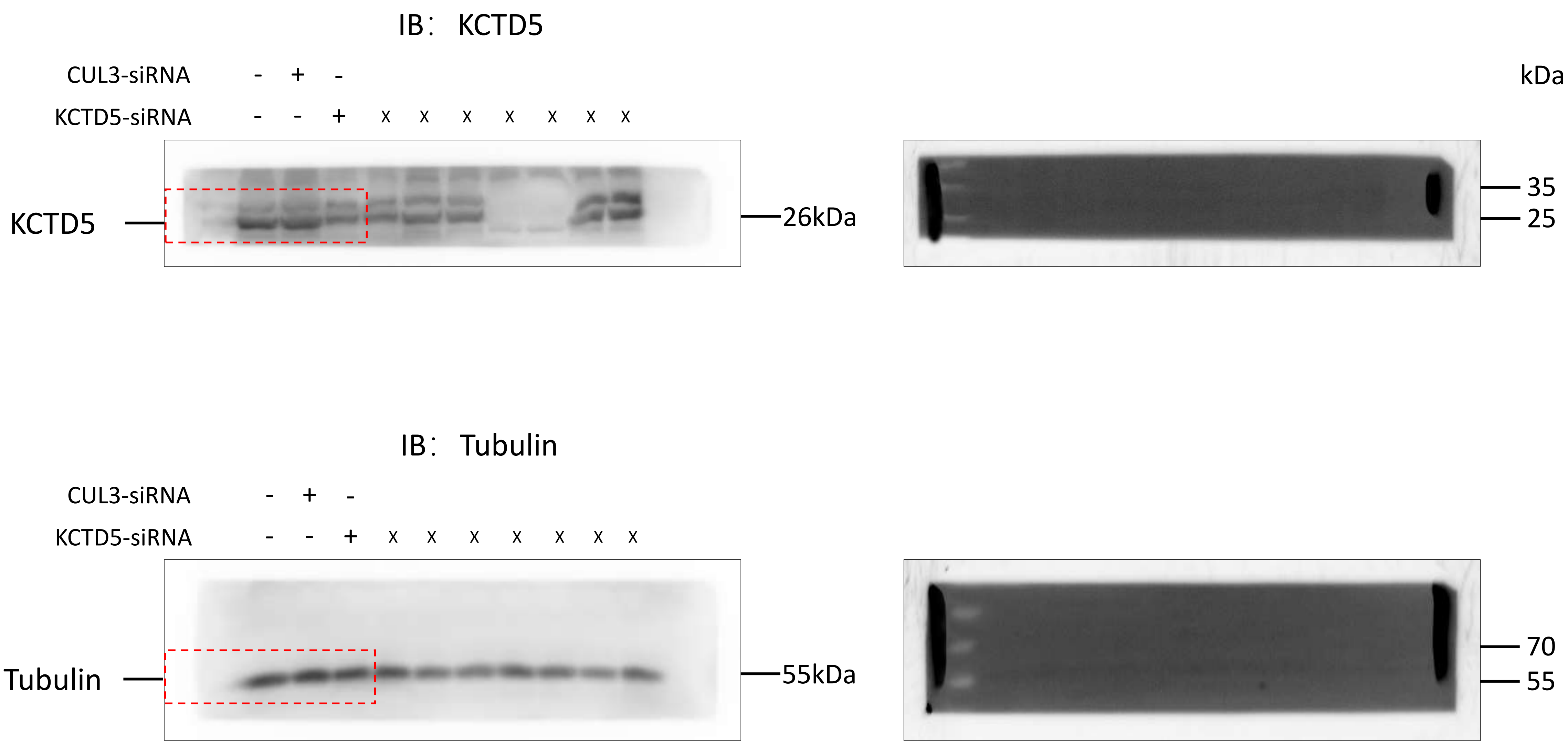
IB： KCTD5
CUL3-siRNA - + -
KCTD5-siRNA - - + x x x x x x x
KCTD5
26kDa
kDa
35
25
IB： Tubulin
CUL3-siRNA - + -
KCTD5-siRNA - - + x x x x x x x
Tubulin
55kDa
70
55

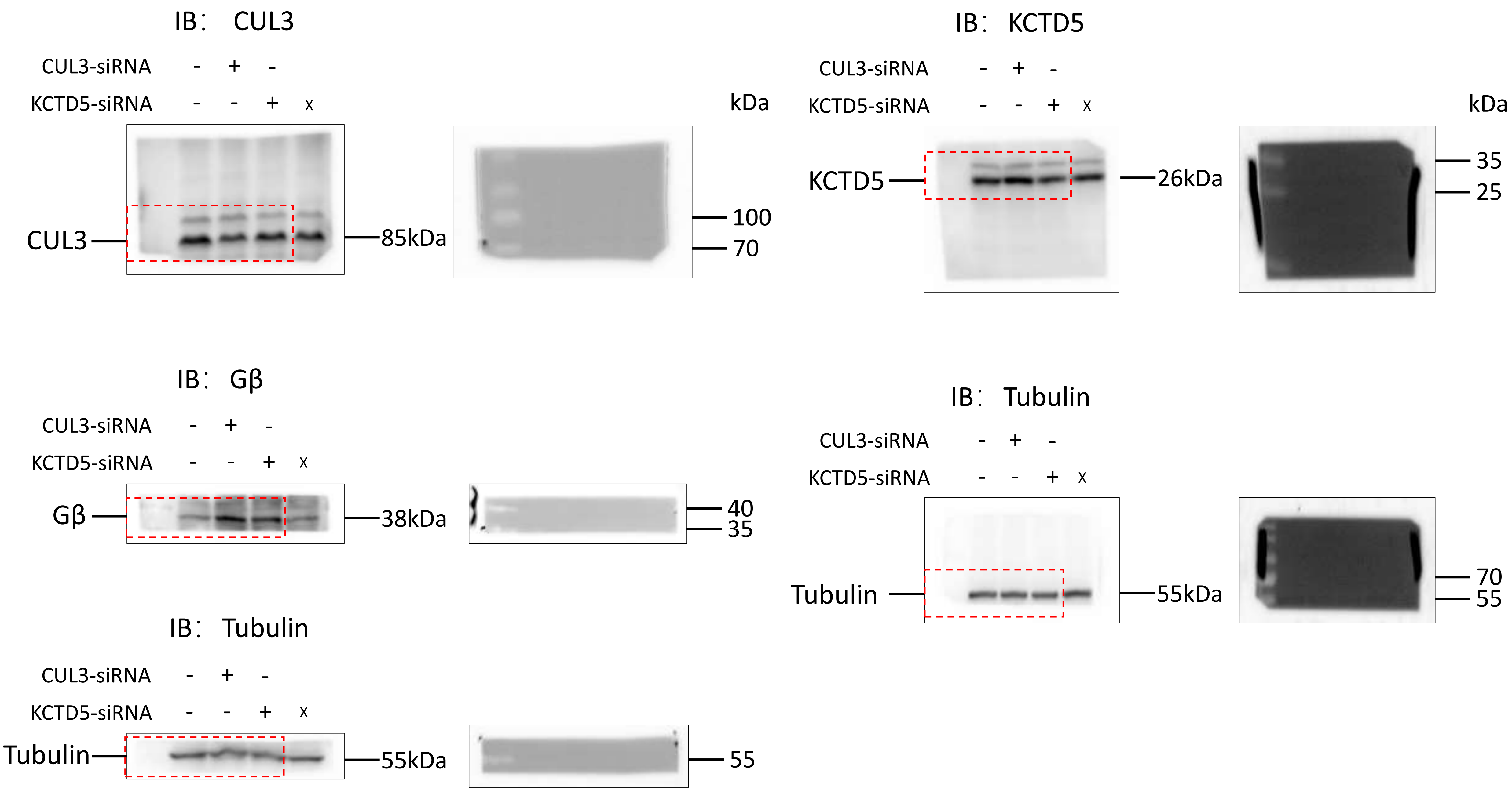
IB: CUL3
CUL3-siRNA - + -
KCTD5-siRNA - - + x
kDa
CUL3
85kDa
100
70
IB: KCTD5
CUL3-siRNA - + -
KCTD5-siRNA - - + x
kDa
KCTD5
26kDa
35
25
IB: Gβ
CUL3-siRNA - + -
KCTD5-siRNA - - + x
Gβ
38kDa
40
35
IB: Tubulin
CUL3-siRNA - + -
KCTD5-siRNA - - + x
Tubulin
55kDa
70
55
IB: Tubulin
CUL3-siRNA - + -
KCTD5-siRNA - - + x
Tubulin
55kDa
55

Figure 2H

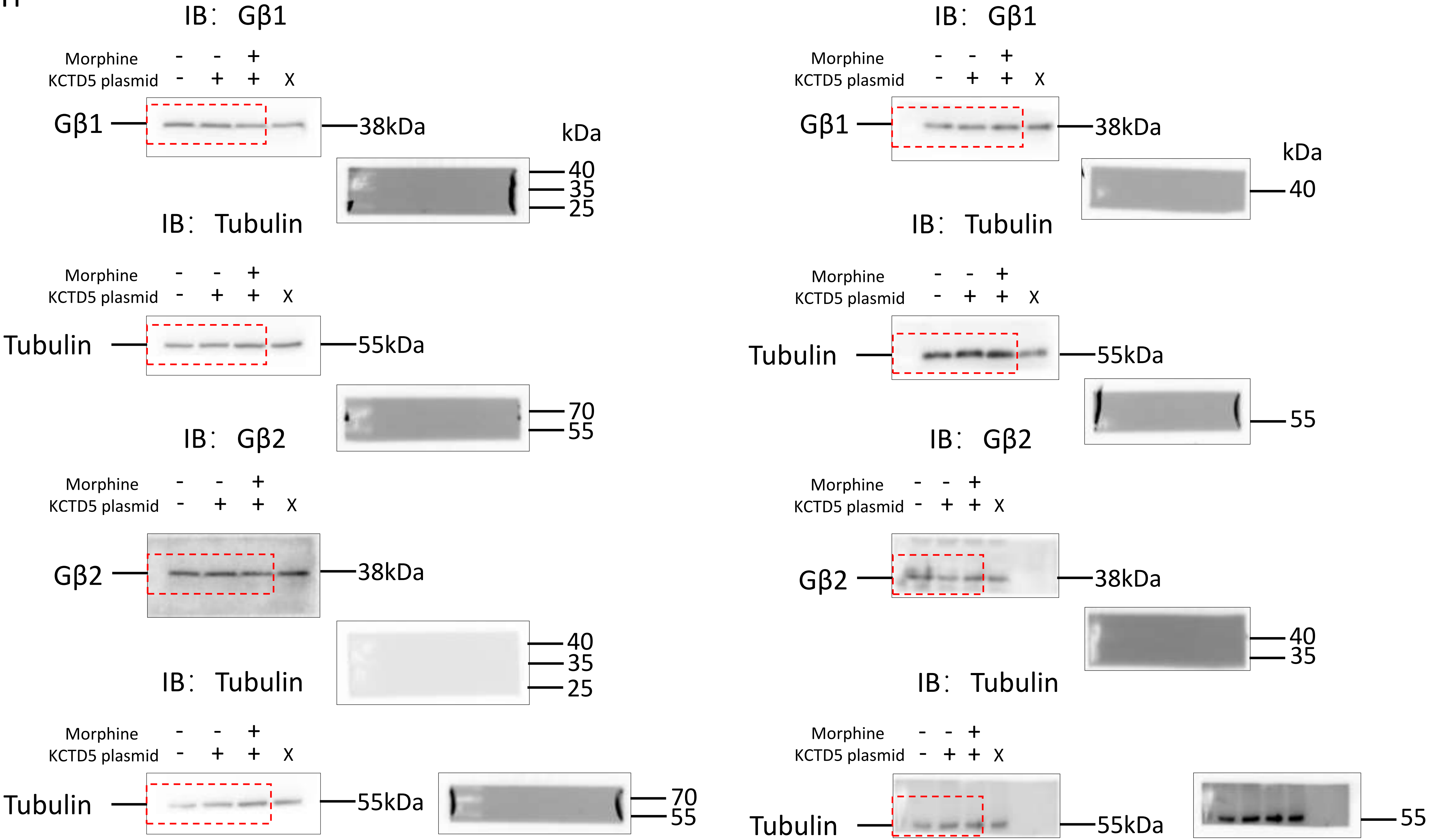
IB: Gβ1
Morphine - - +
KCTD5 plasmid - + + X
Gβ1
38kDa
kDa
40
35
25
IB: Tubulin
Morphine - - +
KCTD5 plasmid - + + X
Tubulin
55kDa
70
55
IB: Gβ2
Morphine - - +
KCTD5 plasmid - + + X
Gβ2
38kDa
40
35
25
IB: Tubulin
Morphine - - +
KCTD5 plasmid - + + X
Tubulin
55kDa
70
55
IB: Gβ1
Morphine - - +
KCTD5 plasmid - + + X
Gβ1
38kDa
kDa
40
IB: Tubulin
Morphine - - +
KCTD5 plasmid - + + X
Tubulin
55kDa
55
IB: Gβ2
Morphine - - +
KCTD5 plasmid - + + X
Gβ2
38kDa
40
35
IB: Tubulin
Morphine - - +
KCTD5 plasmid - + + X
Tubulin
55kDa
55

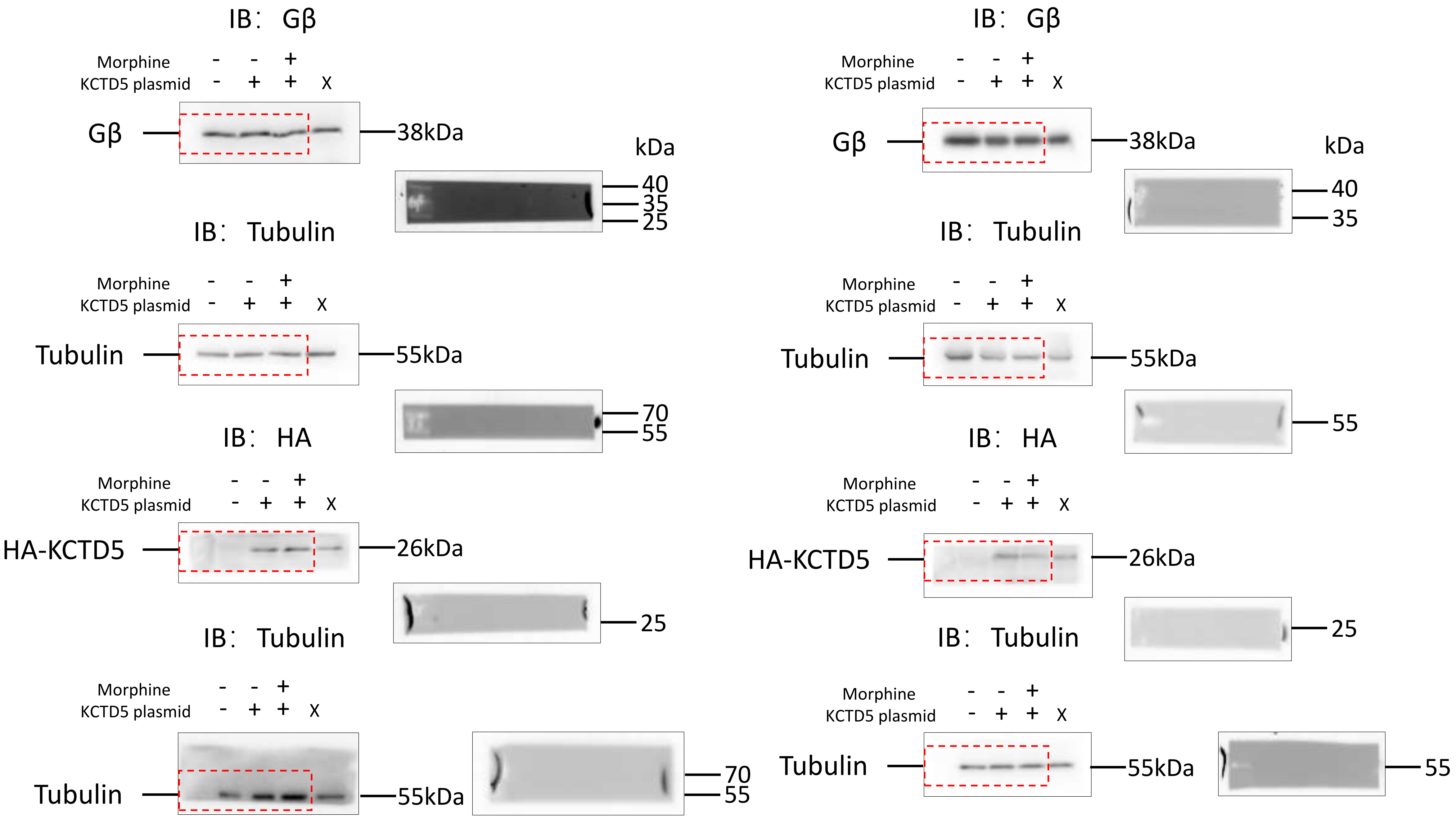
IB: Gβ
Morphine - - +
KCTD5 plasmid - + + X
Gβ
38kDa
kDa
40
35
25
IB: Tubulin
Morphine - - +
KCTD5 plasmid - + + X
Tubulin
55kDa
70
55
IB: HA
Morphine - - +
KCTD5 plasmid - + + X
HA-KCTD5
26kDa
25
IB: Tubulin
Morphine - - +
KCTD5 plasmid - + + X
Tubulin
55kDa
70
55
IB: Gβ
Morphine - - +
KCTD5 plasmid - + + X
Gβ
38kDa
kDa
40
35
IB: Tubulin
Morphine - - +
KCTD5 plasmid - + + X
Tubulin
55kDa
55
IB: HA
Morphine - - +
KCTD5 plasmid - + + X
HA-KCTD5
26kDa
25
IB: Tubulin
Morphine - - +
KCTD5 plasmid - + + X
Tubulin
55kDa
55

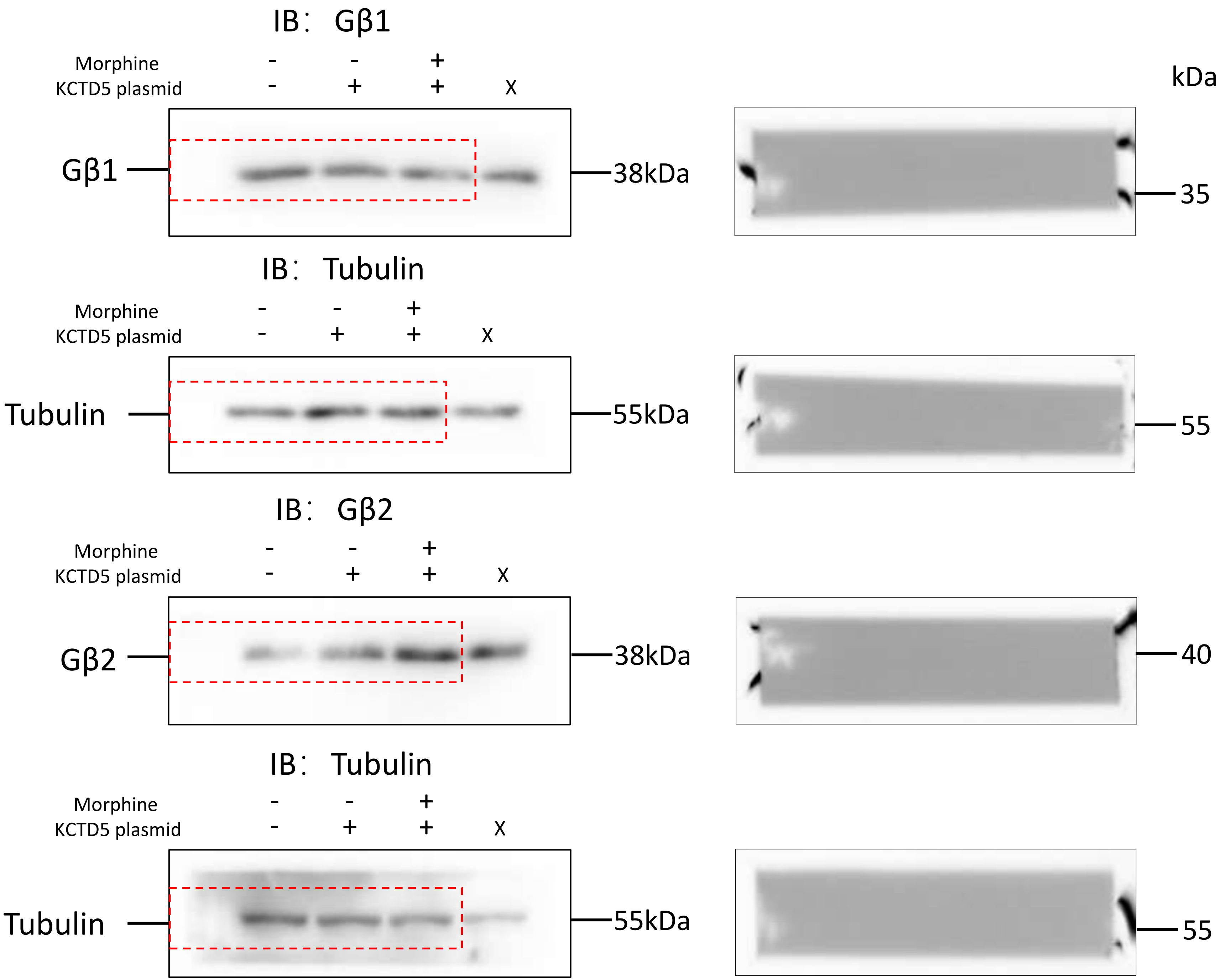
IB: Gβ1
Morphine - - +
KCTD5 plasmid - + + X
Gβ1
38kDa
kDa
35
IB: Tubulin
Morphine - - +
KCTD5 plasmid - + + X
Tubulin
55kDa
55
IB: Gβ2
Morphine - - +
KCTD5 plasmid - + + X
Gβ2
38kDa
40
IB: Tubulin
Morphine - - +
KCTD5 plasmid - + + X
Tubulin
55kDa
55

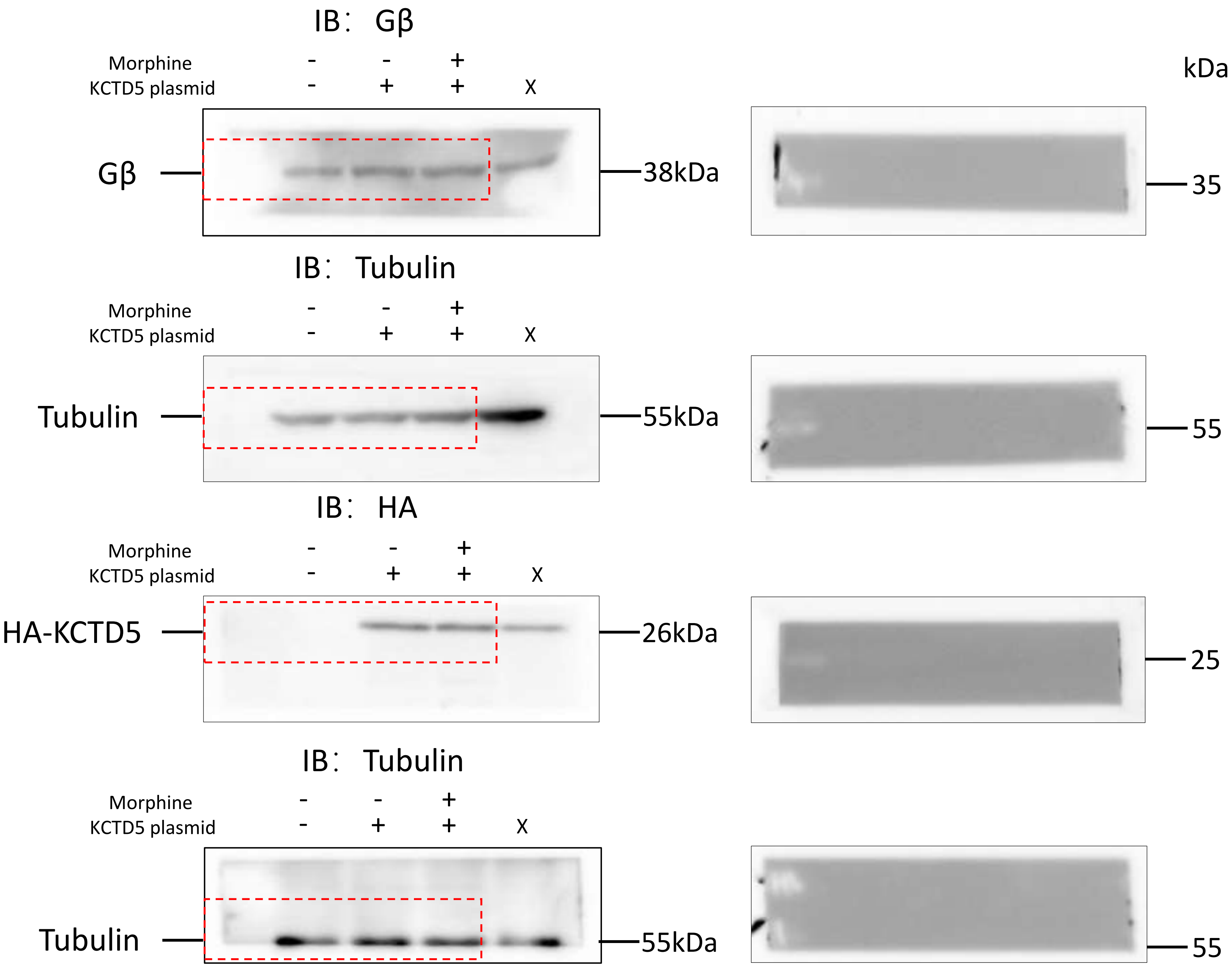
IB: Gβ
Morphine - - +
KCTD5 plasmid - + + X
Gβ
38kDa
kDa
35
IB: Tubulin
Morphine - - +
KCTD5 plasmid - + + X
Tubulin
55kDa
55
IB: HA
Morphine - - +
KCTD5 plasmid - + + X
HA-KCTD5
26kDa
25
IB: Tubulin
Morphine - - +
KCTD5 plasmid - + + X
Tubulin
55kDa
55

Figure 3E

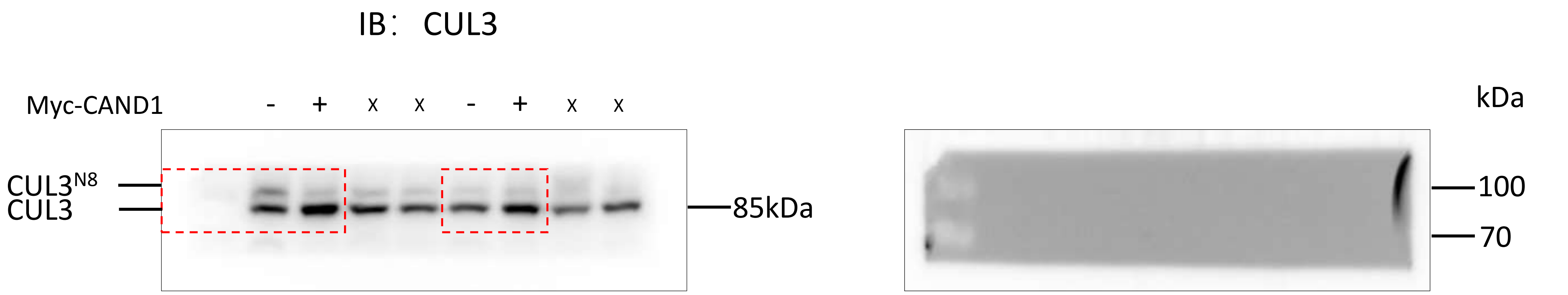
IB: CUL3
Myc-CAND1 - + x x - + x x
kDa
CUL3N8
CUL3
85kDa
100
70

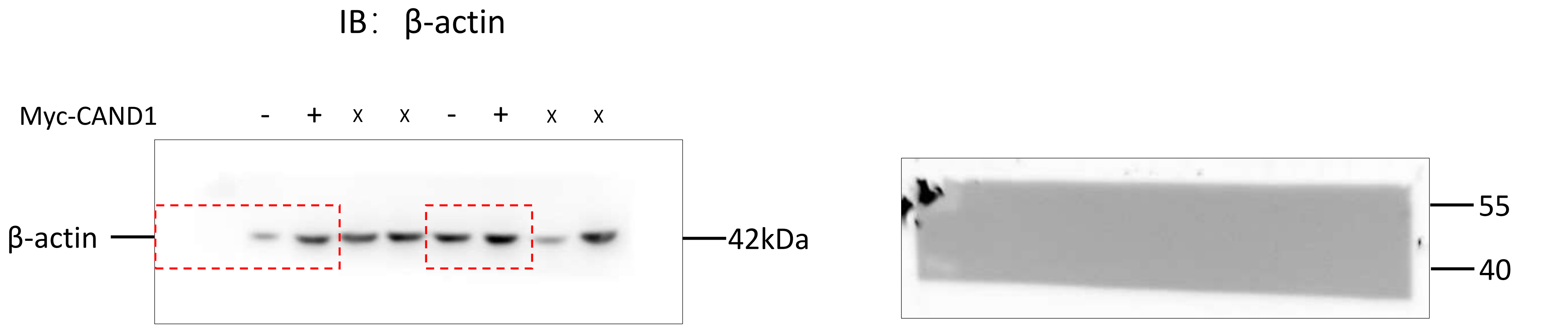
IB: β-actin
Myc-CAND1 - + x x - + x x
β-actin
42kDa
55
40

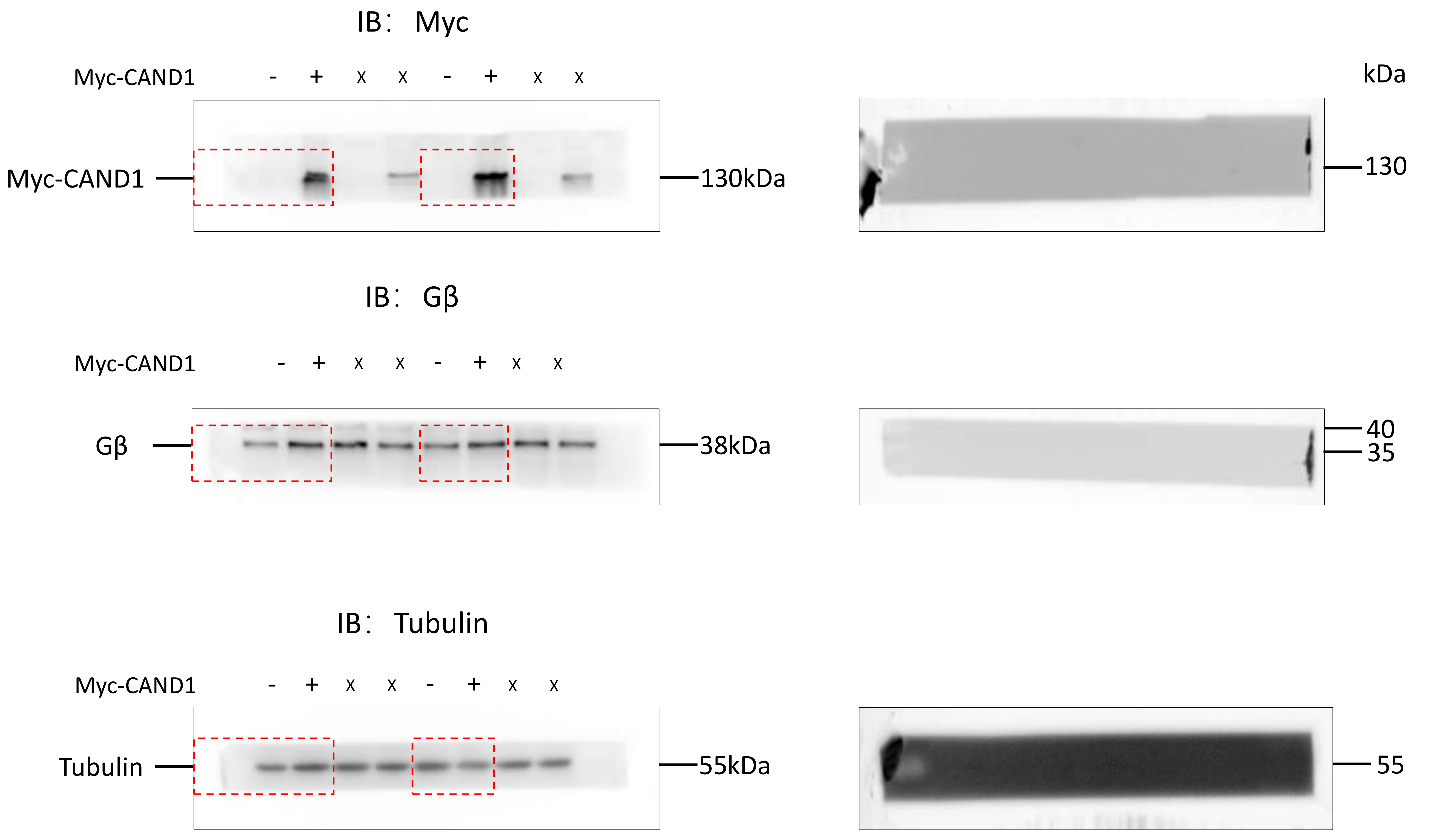
IB： Myc
Myc-CAND1 - + x x - + x x
kDa
Myc-CAND1
130kDa
130
IB： Gβ
Myc-CAND1 - + x x - + x x
Gβ
38kDa
40
35
IB： Tubulin
Myc-CAND1 - + x x - + x x
Tubulin
55kDa
55

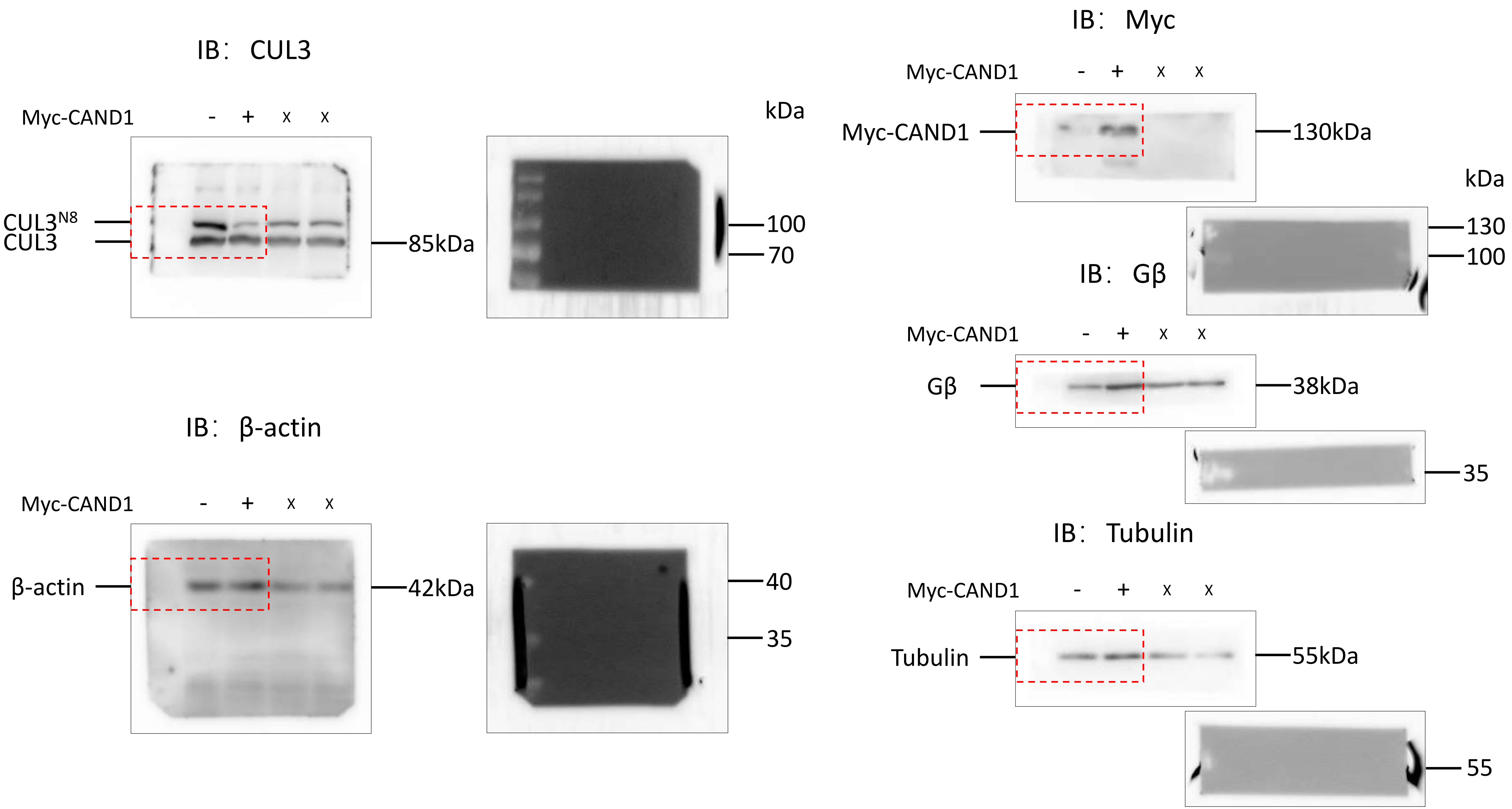
IB： CUL3
Myc-CAND1 - + x x
CUL3N8
CUL3
85kDa
kDa
100
70
IB： β-actin
Myc-CAND1 - + x x
β-actin
42kDa
40
35
IB： Myc
Myc-CAND1 - + x x
Myc-CAND1
130kDa
kDa
130
100
IB： Gβ
Myc-CAND1 - + x x
Gβ
38kDa
35
IB： Tubulin
Myc-CAND1 - + x x
Tubulin
55kDa
55

Figure 3F

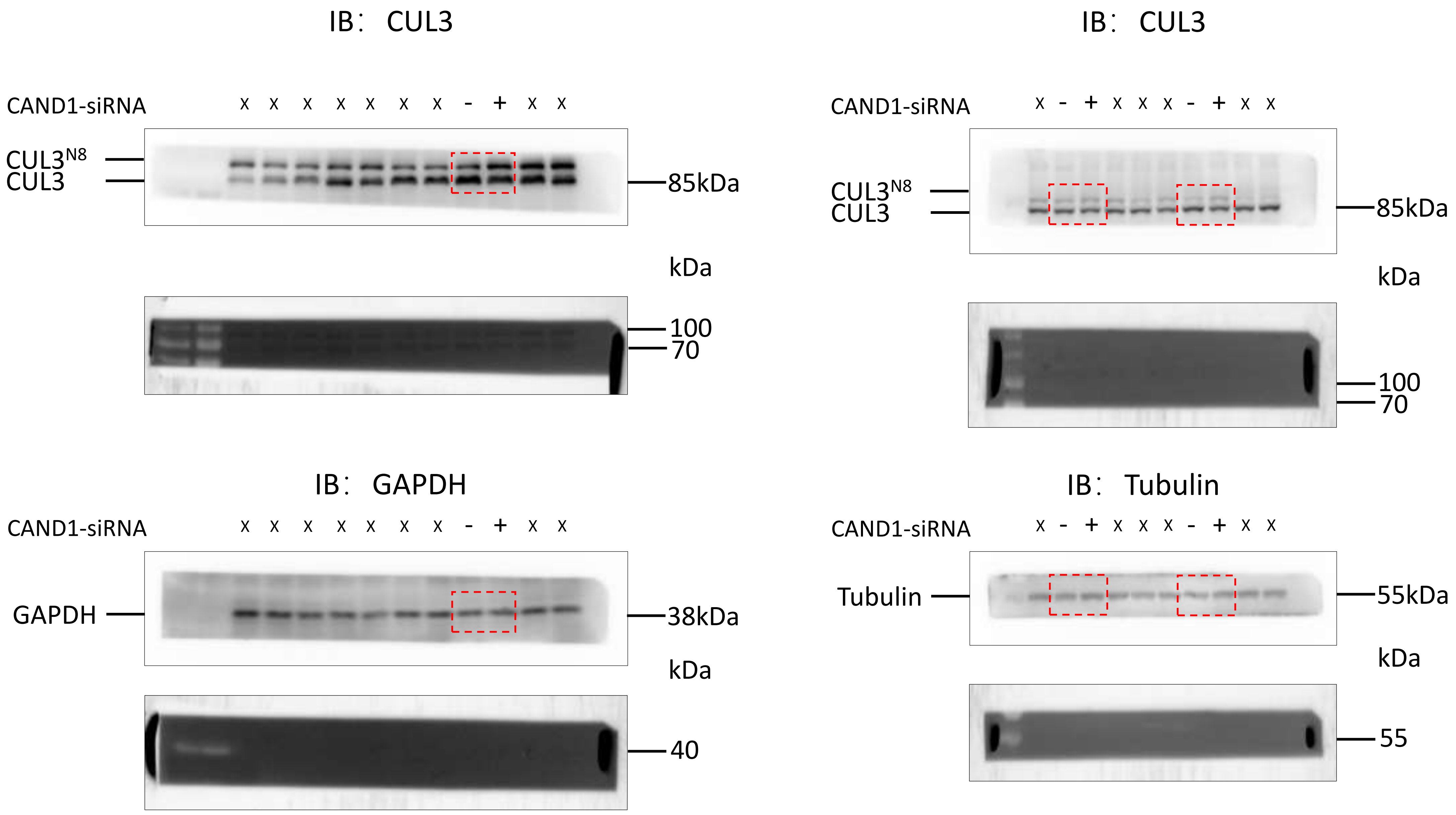
IB： CUL3
CAND1-siRNA x x x x x x x - + x x
CUL3^{N8}
CUL3
85kDa
kDa
100
70
IB： CUL3
CAND1-siRNA x - + x x x - + x x
CUL3^{N8}
CUL3
85kDa
kDa
100
70
IB： GAPDH
CAND1-siRNA x x x x x x x - + x x
GAPDH
38kDa
kDa
40
IB： Tubulin
CAND1-siRNA x - + x x x - + x x
Tubulin
55kDa
kDa
55

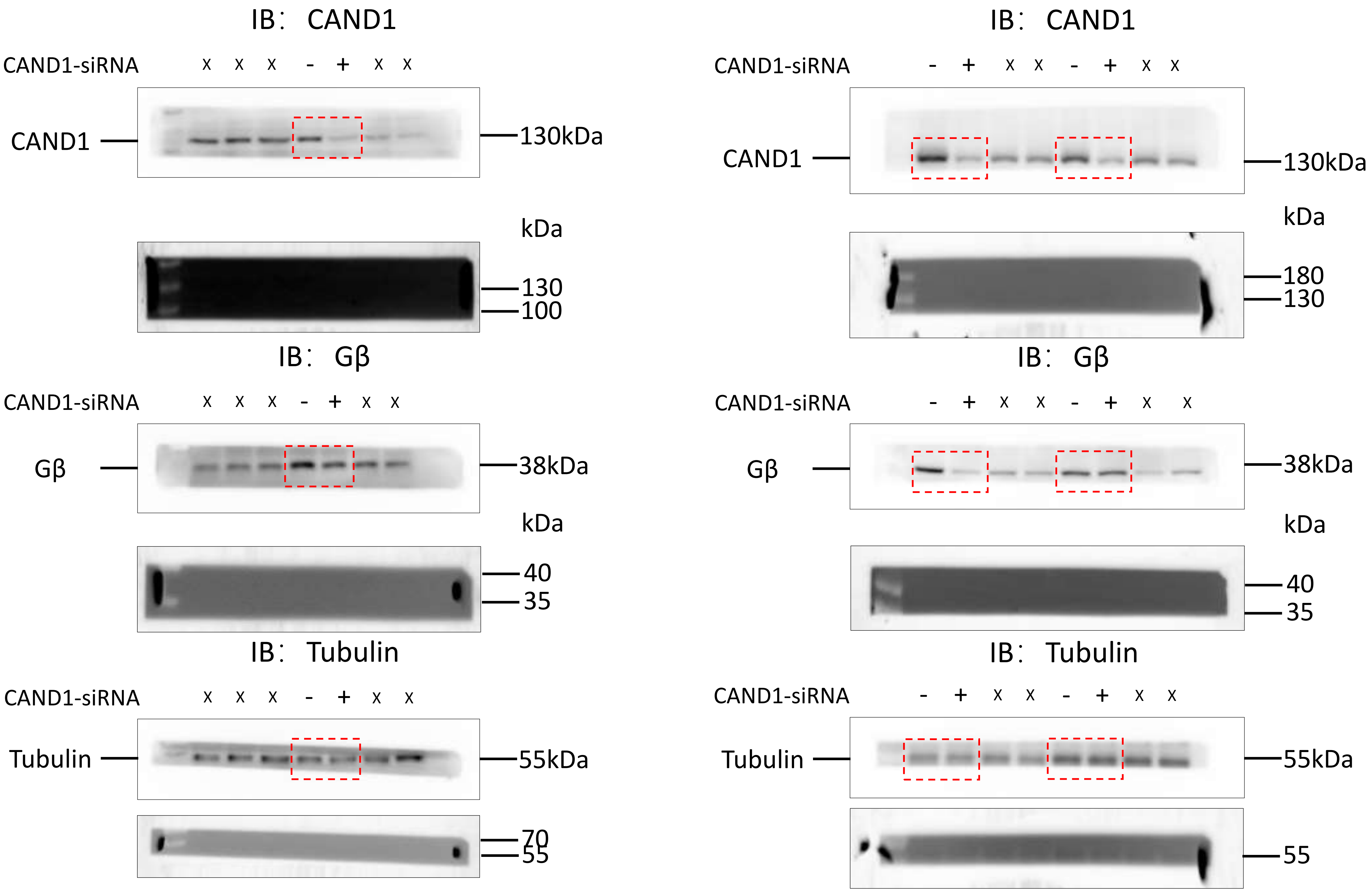
IB： CAND1
CAND1-siRNA x x x - + x x
CAND1
130kDa
kDa
130
100
IB： Gβ
CAND1-siRNA x x x - + x x
Gβ
38kDa
kDa
40
35
IB： Tubulin
CAND1-siRNA x x x - + x x
Tubulin
55kDa
70
55
IB： CAND1
CAND1-siRNA - + x x - + x x
CAND1
130kDa
kDa
180
130
IB： Gβ
CAND1-siRNA - + x x - + x x
Gβ
38kDa
kDa
40
35
IB： Tubulin
CAND1-siRNA - + x x - + x x
Tubulin
55kDa
55

Figure 3G

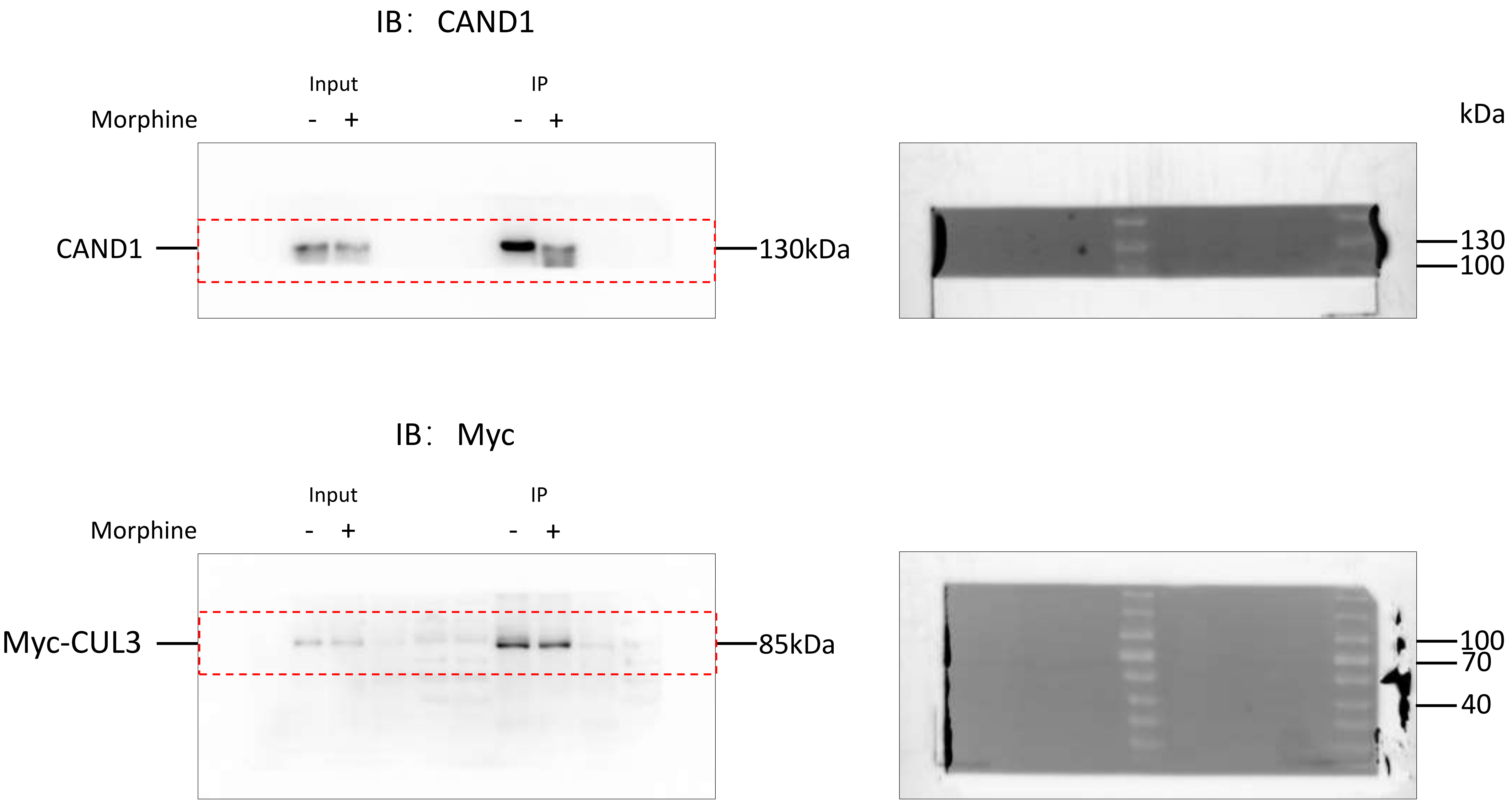
IB: CAND1
Input
IP
Morphine
- + - +
kDa
CAND1
130kDa
130
100
IB: Myc
Input
IP
Morphine
- + - +
Myc-CUL3
85kDa
100
70
40

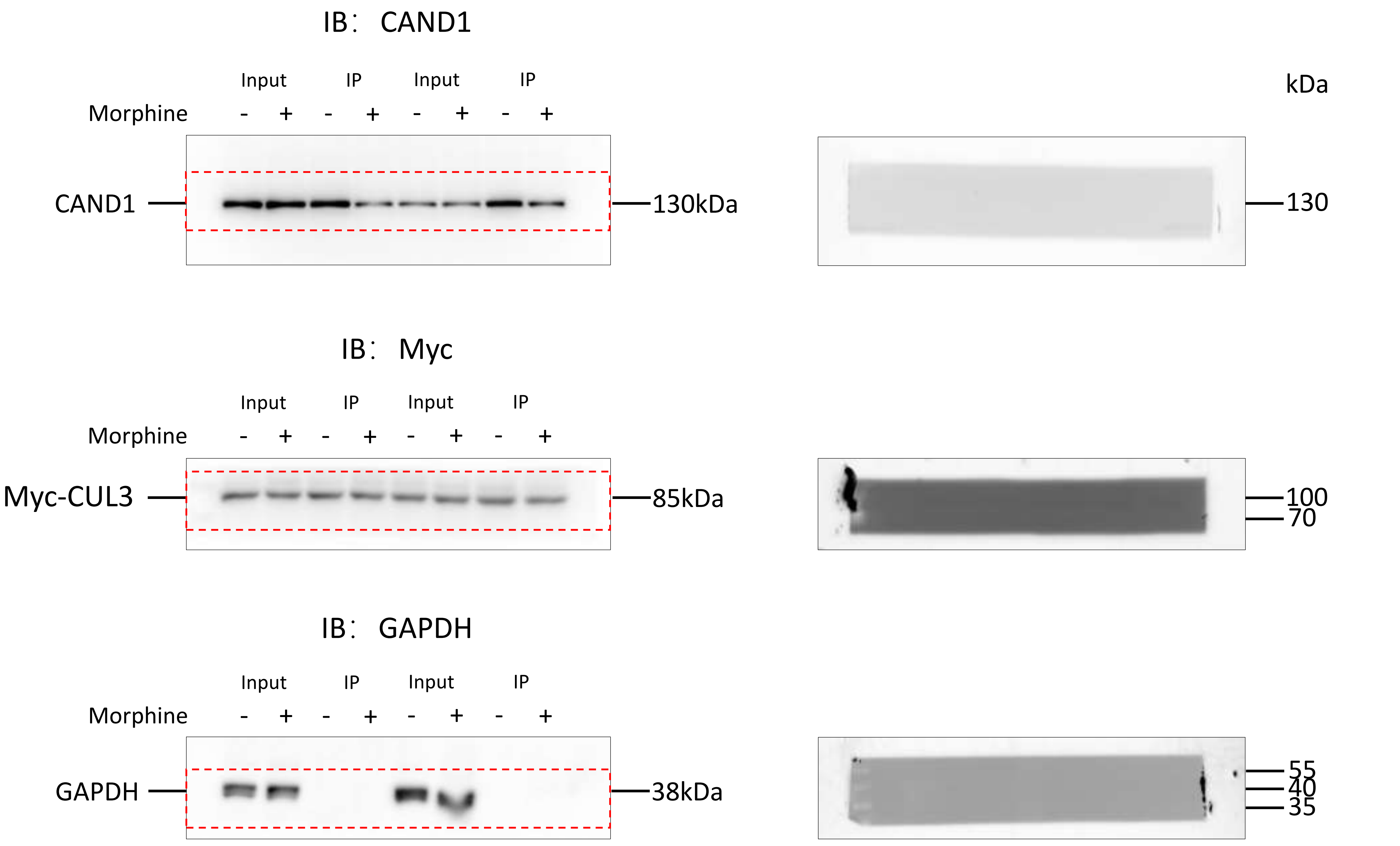
IB： CAND1
Input
IP
Input
IP
kDa
Morphine
- + - + - + - +
CAND1
130kDa
130
IB： Myc
Input
IP
Input
IP
Morphine
- + - + - + - +
Myc-CUL3
85kDa
100
70
IB： GAPDH
Input
IP
Input
IP
Morphine
- + - + - + - +
GAPDH
38kDa
55
40
35

Figure 3H

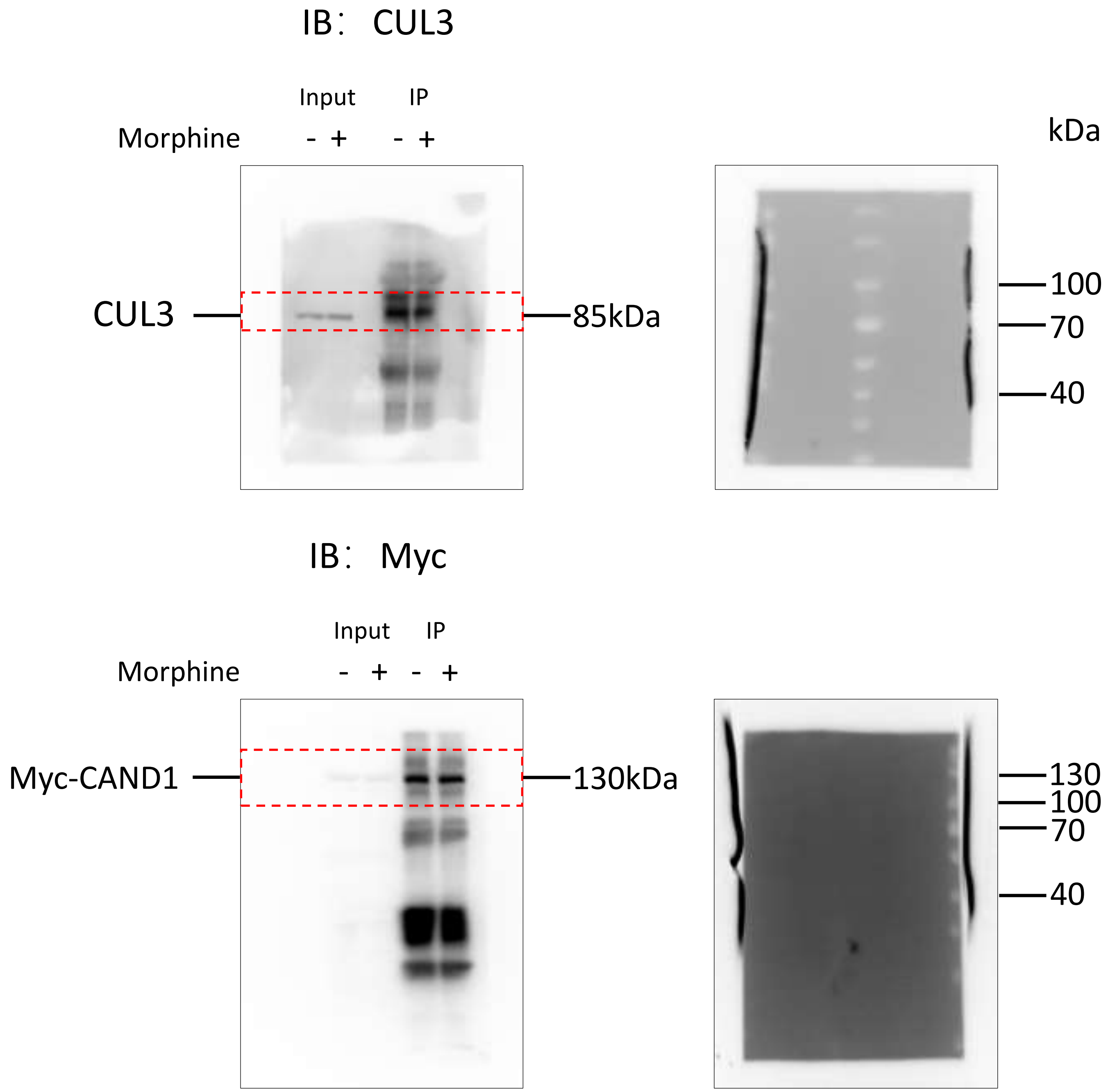

IB： CUL3
Input
IP
Morphine
- + - +
kDa
CUL3
85kDa
100
70
40
IB： Myc
Input
IP
Morphine
- + - +
Myc-CAND1
130kDa
130
100
70
40

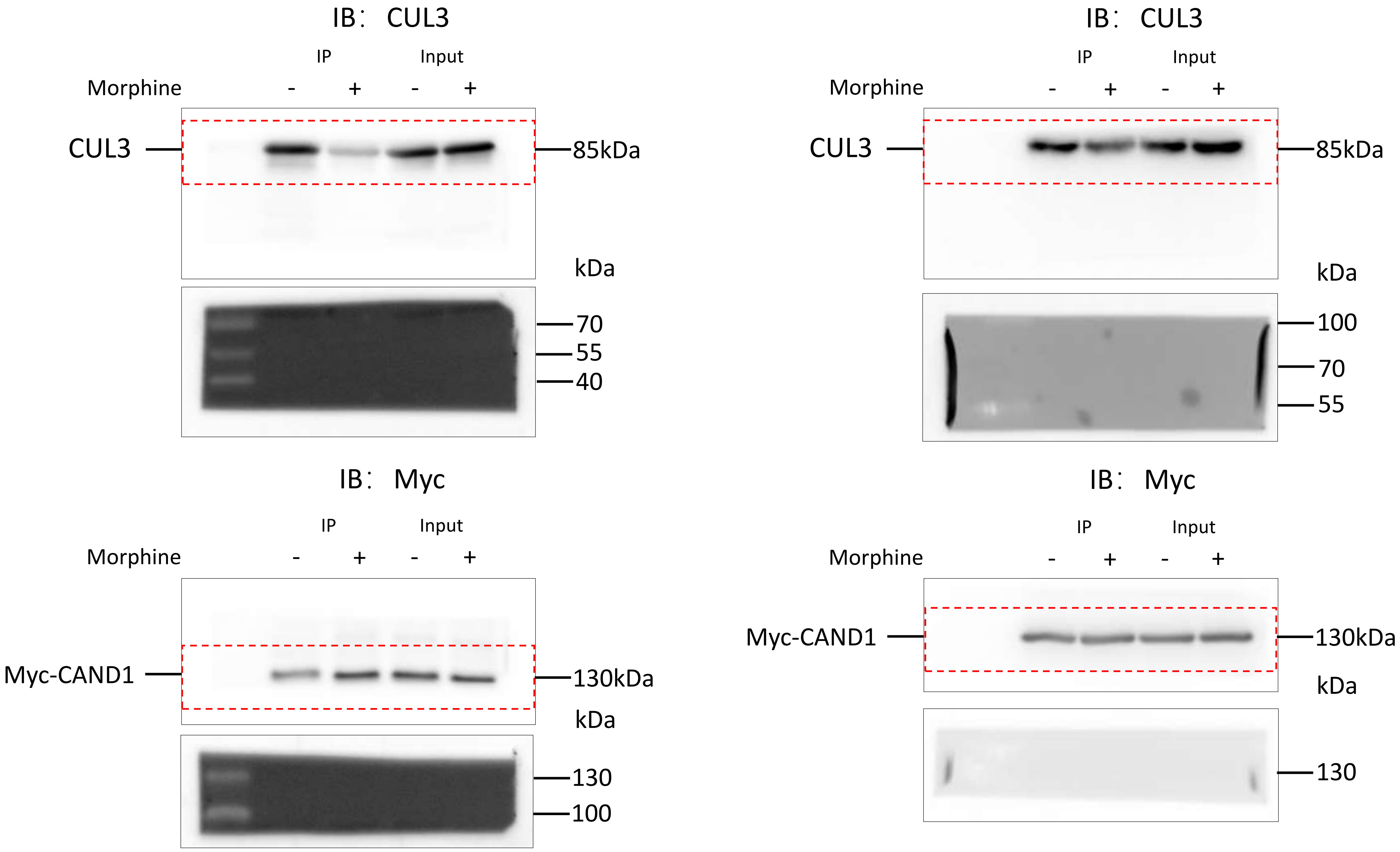
IB: CUL3
IP
Input
Morphine
-
+
-
+
CUL3
85kDa
kDa
70
55
40
IB: CUL3
IP
Input
Morphine
-
+
-
+
CUL3
85kDa
kDa
100
70
55
IB: Myc
IP
Input
Morphine
-
+
-
+
Myc-CAND1
130kDa
kDa
130
100
IB: Myc
IP
Input
Morphine
-
+
-
+
Myc-CAND1
130kDa
kDa
130

Figure 3I

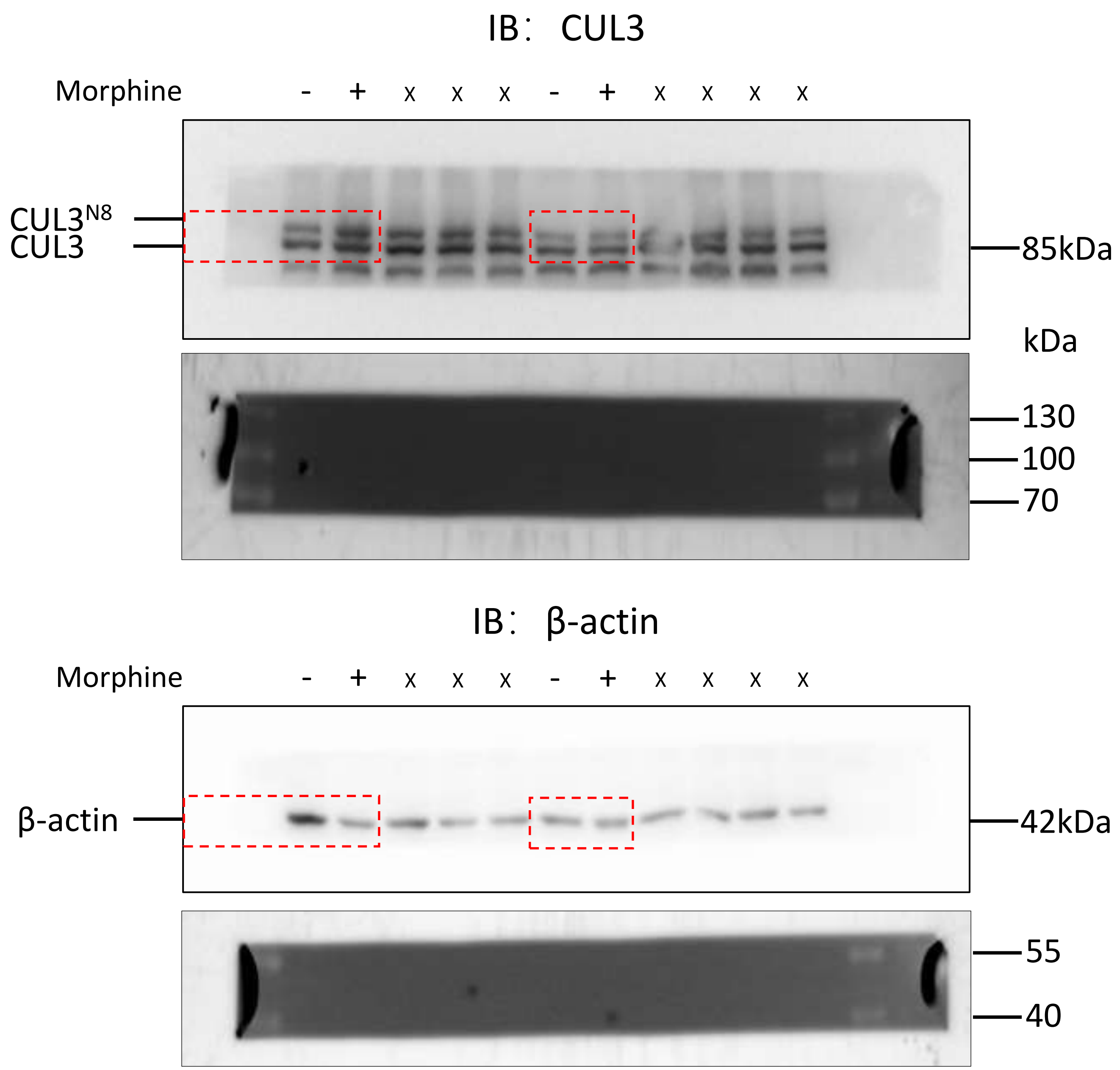
IB: CUL3
Morphine - + x x x - + x x x x
CUL3N8
CUL3
85kDa
kDa
130
100
70
IB: β-actin
Morphine - + x x x - + x x x x
β-actin
42kDa
55
40

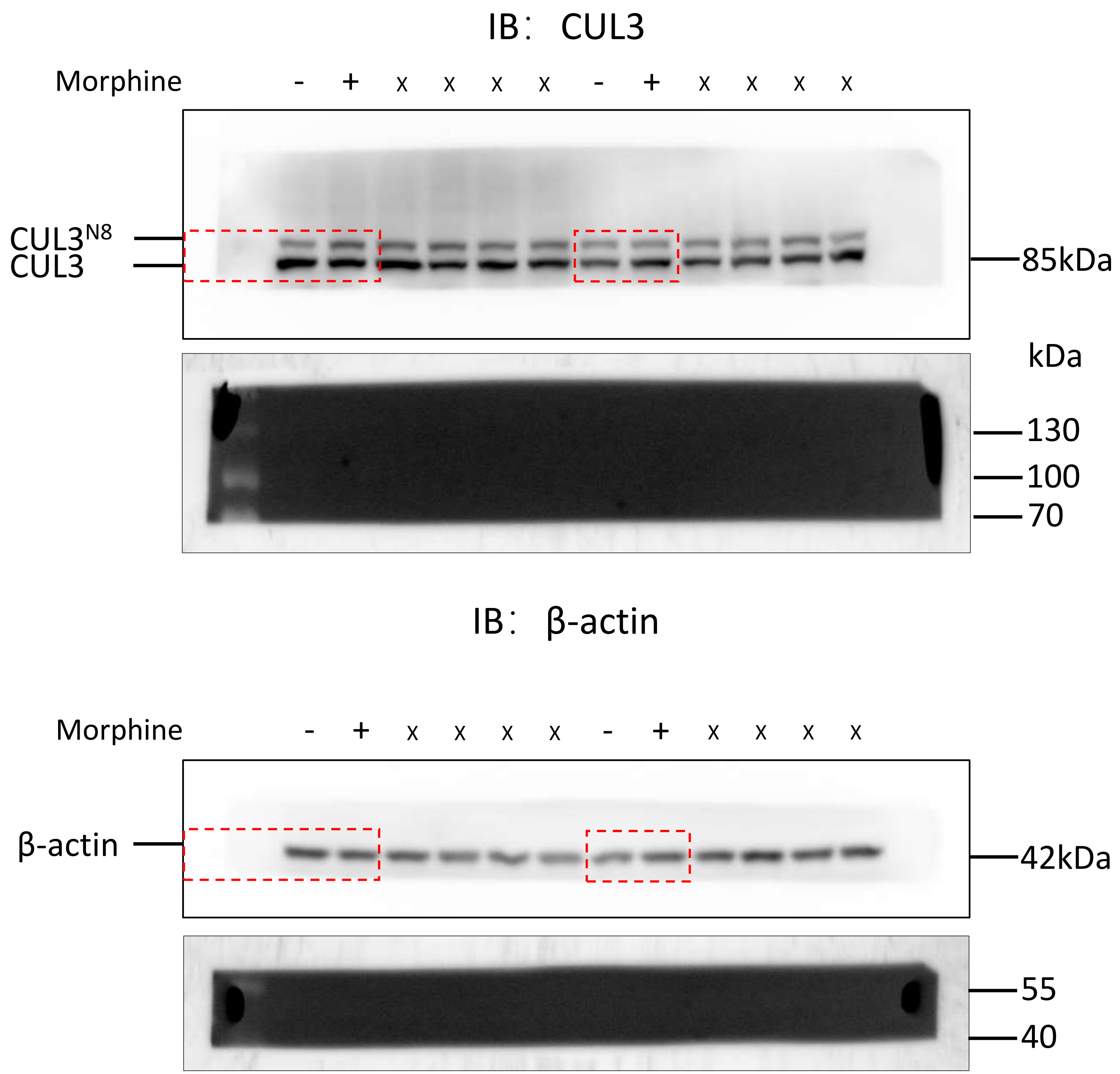
IB：CUL3
Morphine
- + x x x x - + x x x x
CUL3^N8
CUL3
85kDa
kDa
130
100
70
IB：β-actin
Morphine
- + x x x x - + x x x x
β-actin
42kDa
55
40

Figure 3J

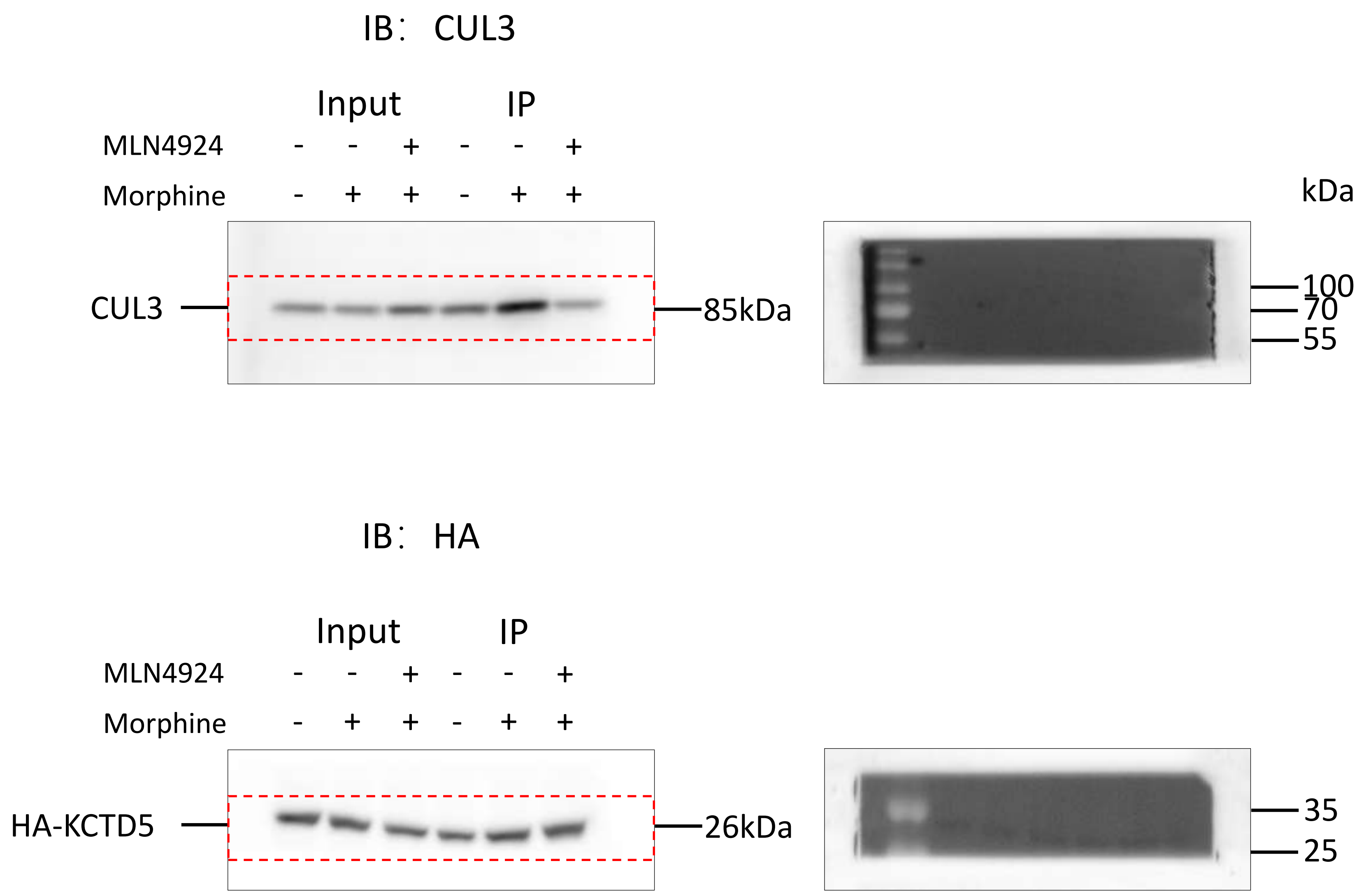
IB: CUL3
Input
IP
MLN4924 - - + - - +
Morphine - + + - + +
kDa
CUL3
85kDa
100
70
55
IB: HA
Input
IP
MLN4924 - - + - - +
Morphine - + + - + +
HA-KCTD5
26kDa
35
25

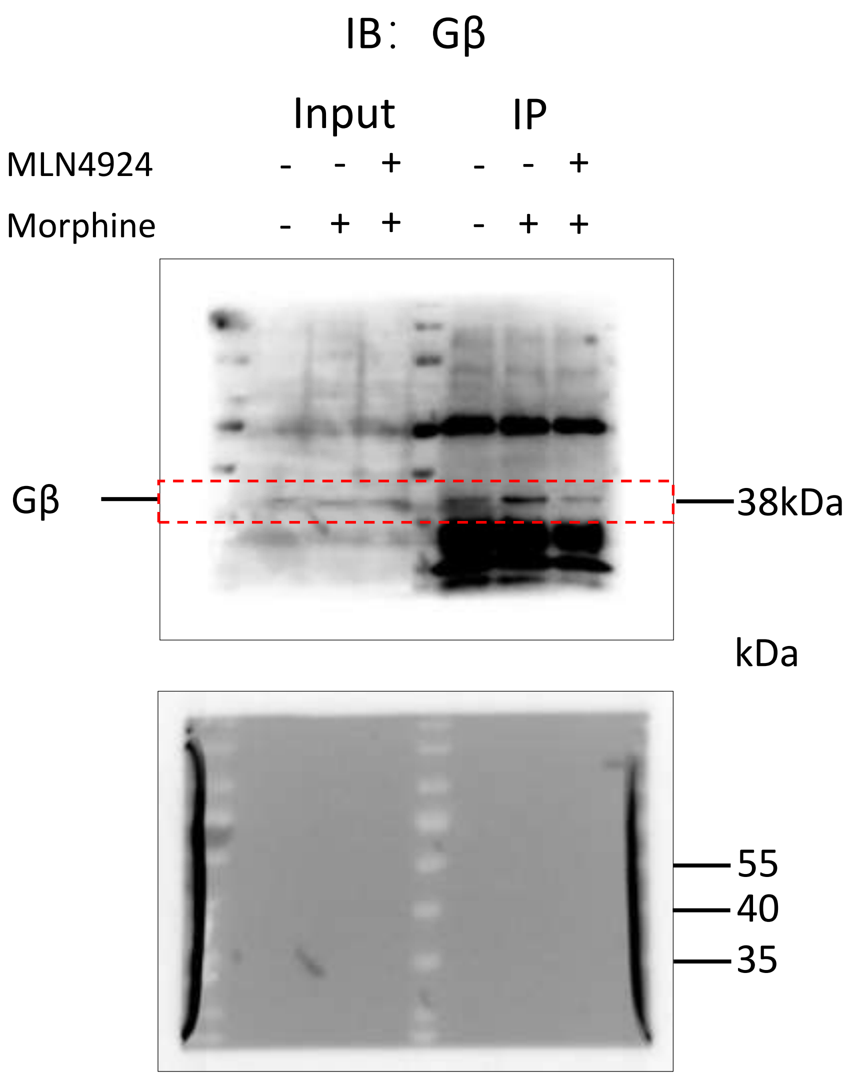
IB: Gβ
Input
IP
MLN4924 - - + - - +
Morphine - + + - + +
Gβ
38kDa
kDa
55
40
35

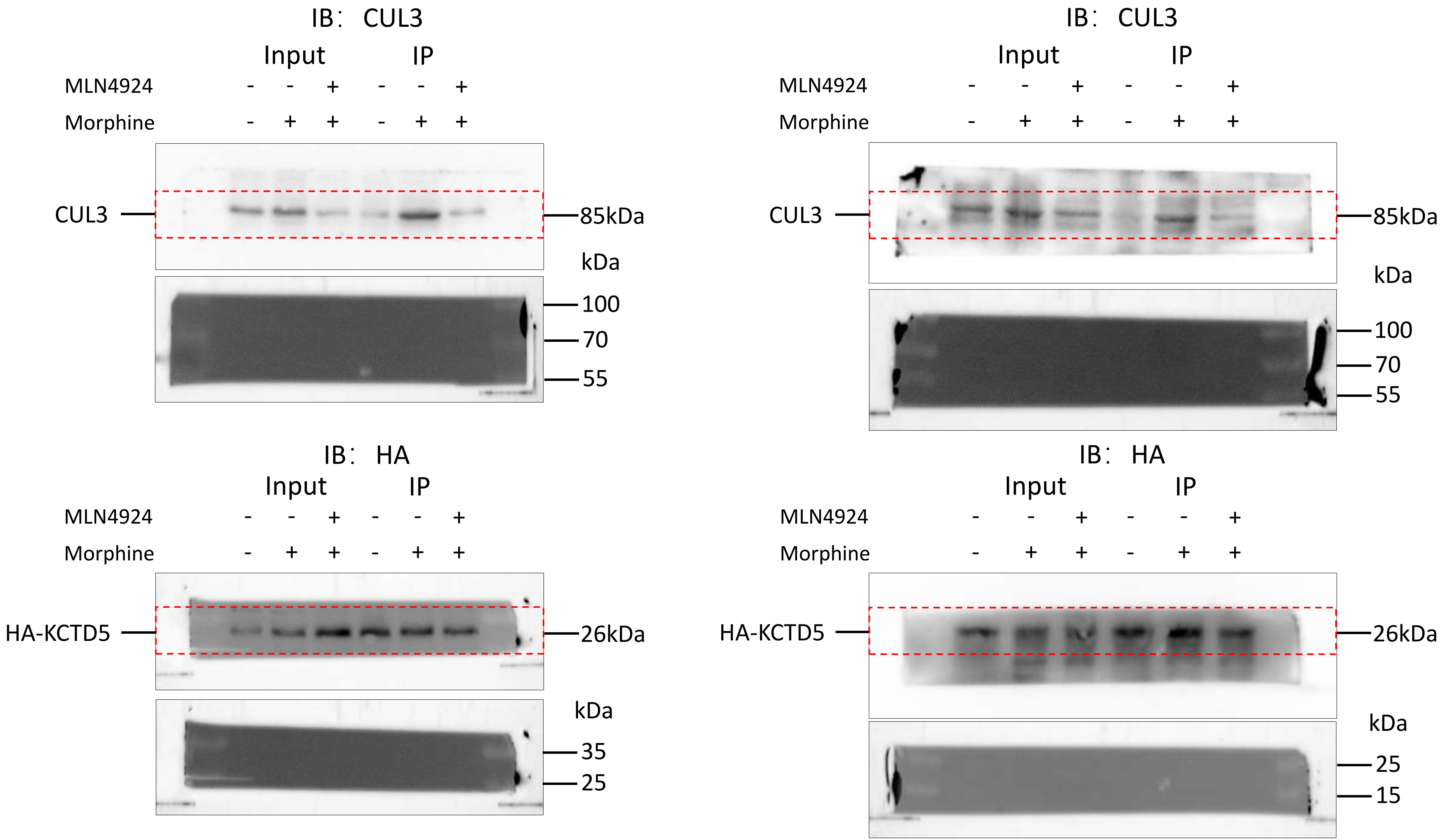
IB： CUL3
Input
IP
MLN4924
- - + - - +
Morphine
- + + - + +
CUL3
85kDa
kDa
100
70
55
IB： CUL3
Input
IP
MLN4924
- - + - - +
Morphine
- + + - + +
CUL3
85kDa
kDa
100
70
55
IB： HA
Input
IP
MLN4924
- - + - - +
Morphine
- + + - + +
HA-KCTD5
26kDa
kDa
35
25
IB： HA
Input
IP
MLN4924
- - + - - +
Morphine
- + + - + +
HA-KCTD5
26kDa
kDa
25
15

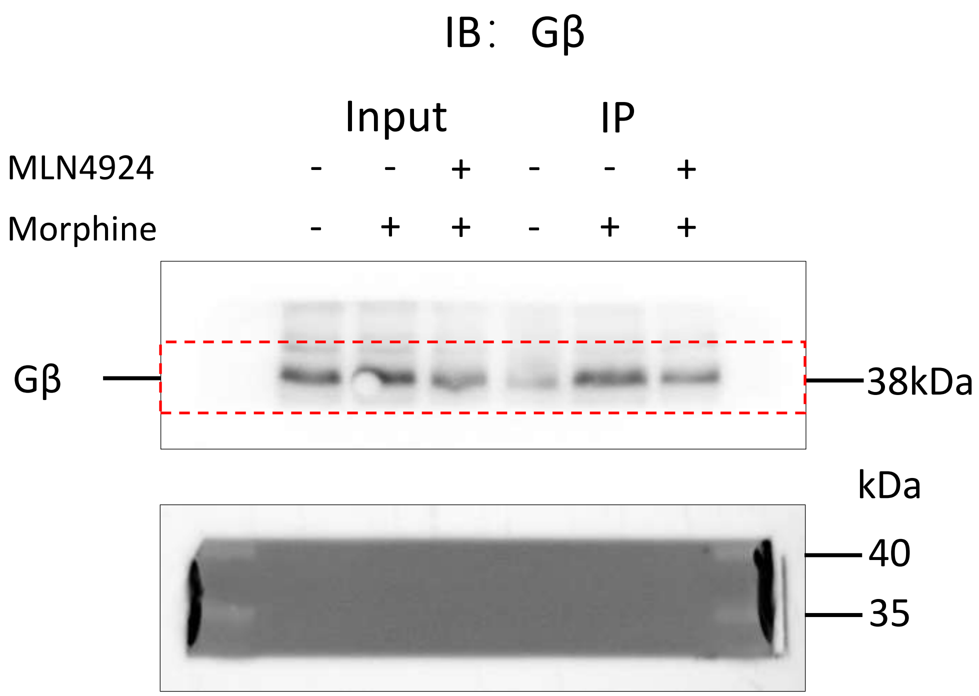
IB： Gβ
Input
IP
MLN4924 - - + - - +
Morphine - + + - + +
Gβ
38kDa
kDa
40
35

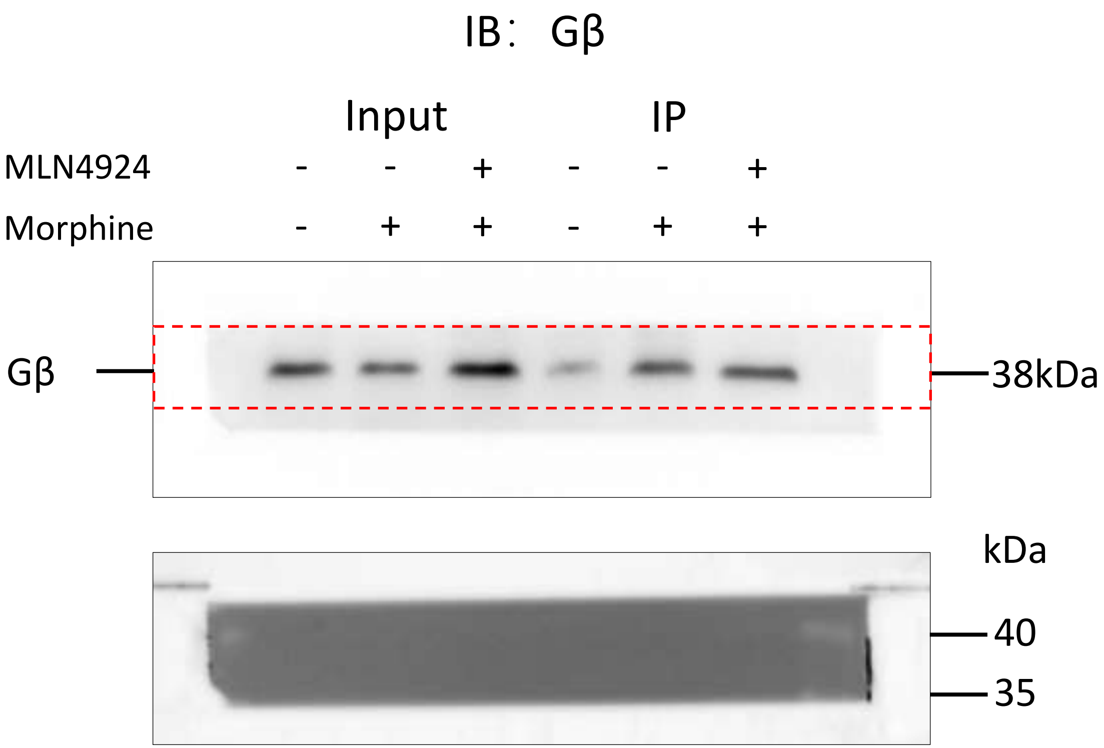
IB： Gβ
Input
IP
MLN4924 - - + - - +
Morphine - + + - + +
Gβ
38kDa
kDa
40
35

Figure 3K

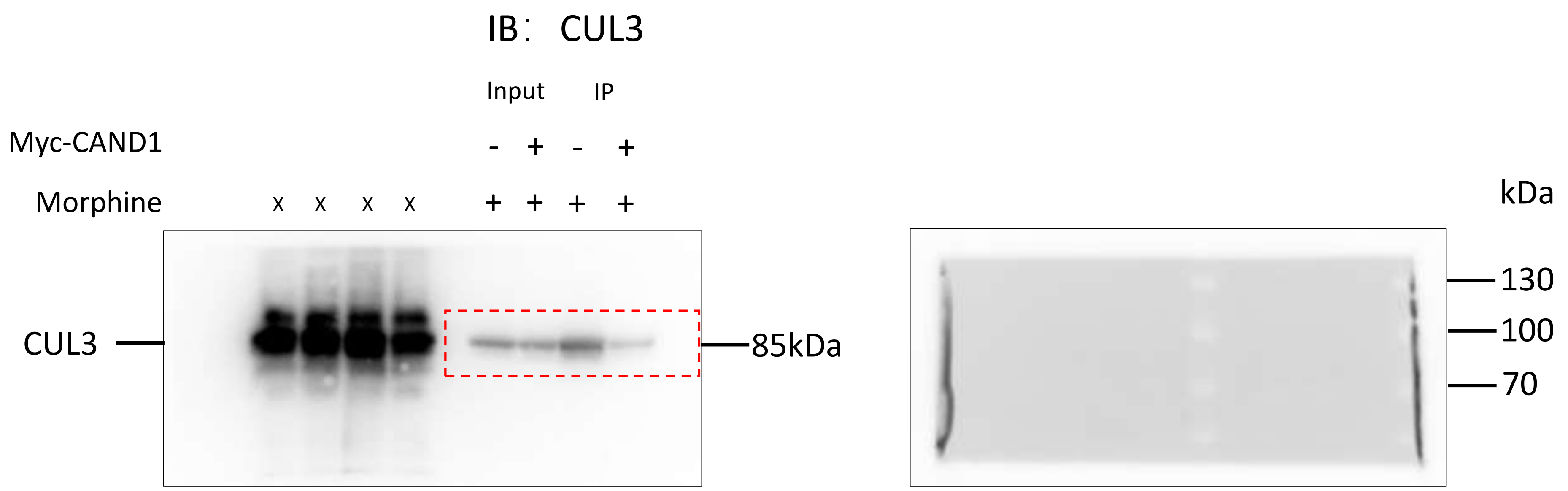
IB： CUL3
Input
IP
Myc-CAND1
- + - +
Morphine
x x x x
+ + + +
CUL3
85kDa
kDa
130
100
70

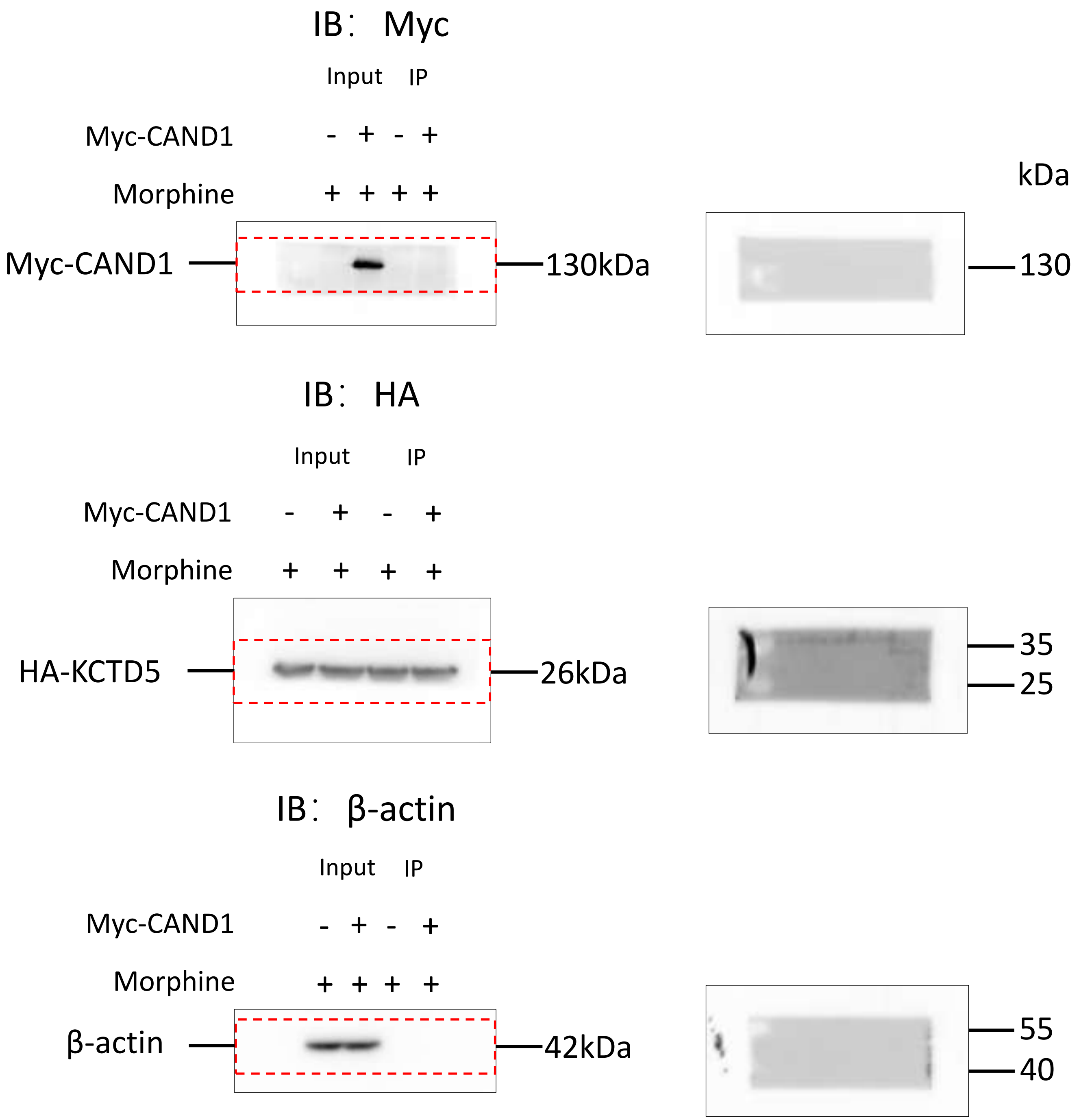
IB： Myc
Input IP
Myc-CAND1 - + - +
Morphine + + + +
Myc-CAND1
130kDa
kDa
130
IB： HA
Input IP
Myc-CAND1 - + - +
Morphine + + + +
HA-KCTD5
26kDa
35
25
IB： β-actin
Input IP
Myc-CAND1 - + - +
Morphine + + + +
β-actin
42kDa
55
40

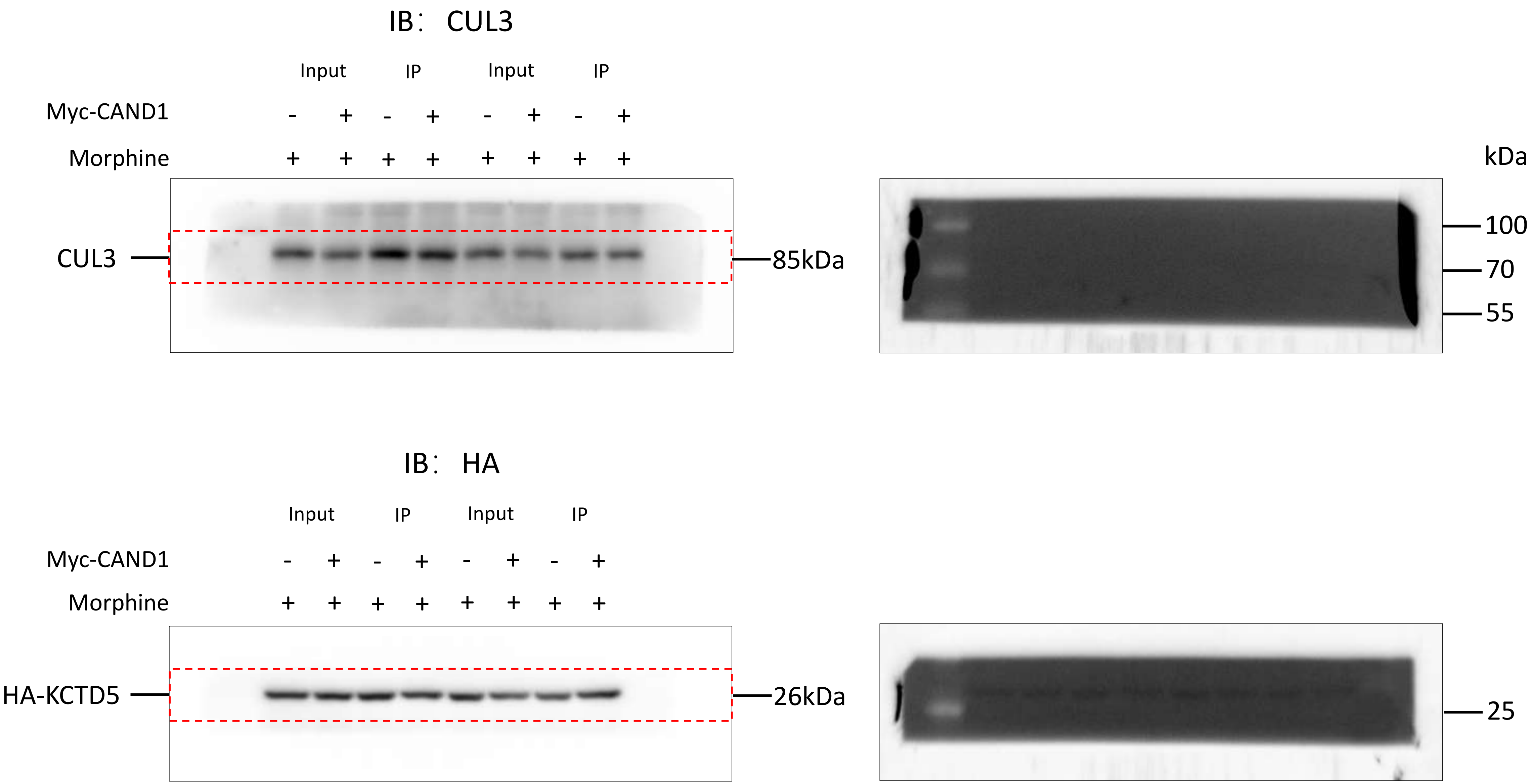
IB： CUL3
Input
IP
Input
IP
Myc-CAND1
- + - + - + - +
Morphine
+ + + + + + + +
CUL3
85kDa
kDa
100
70
55
IB： HA
Input
IP
Input
IP
Myc-CAND1
- + - + - + - +
Morphine
+ + + + + + + +
HA-KCTD5
26kDa
25

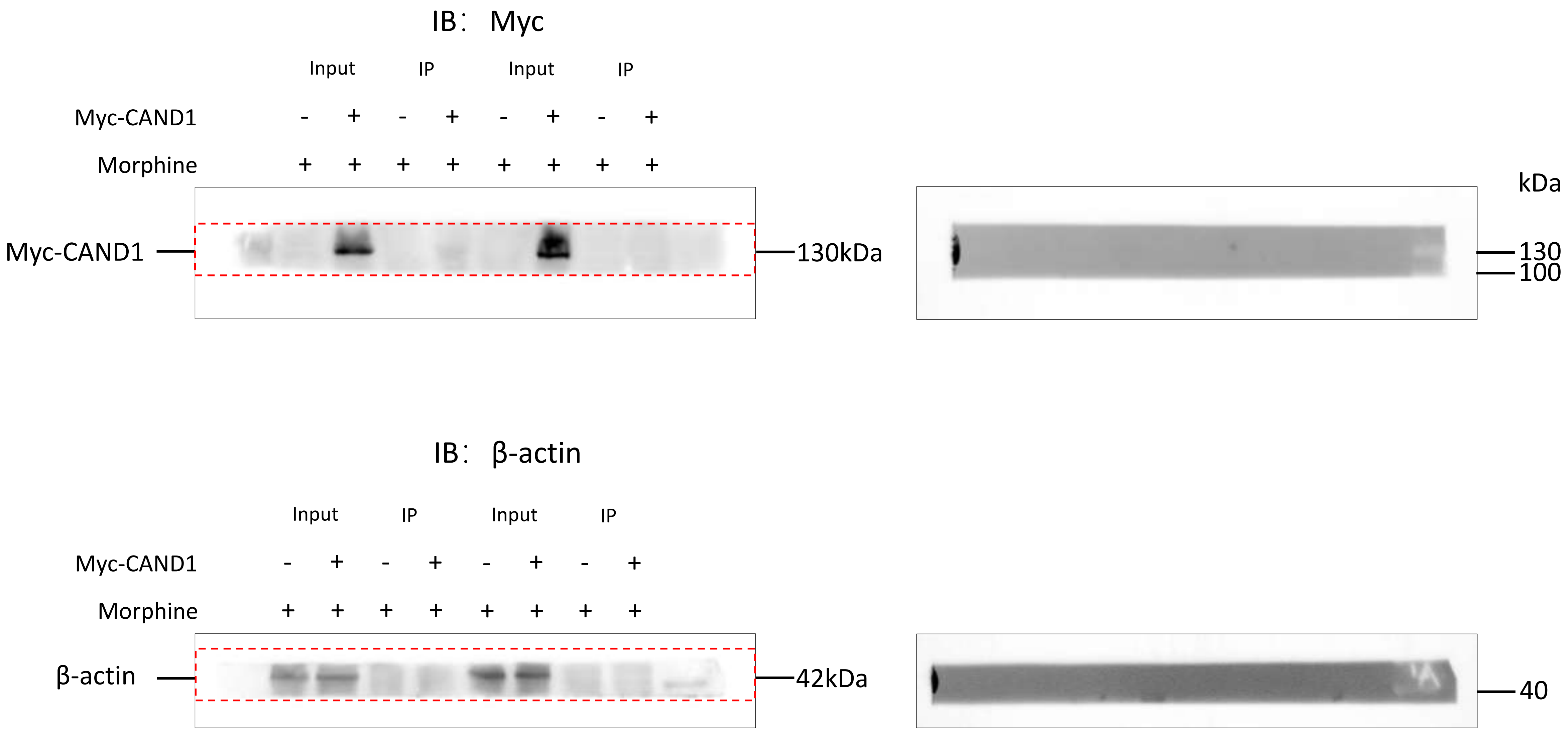
IB: Myc
Input IP Input IP
Myc-CAND1 - + - + - + - +
Morphine + + + + + + + +
Myc-CAND1
130kDa
kDa
130
100
IB: β-actin
Input IP Input IP
Myc-CAND1 - + - + - + - +
Morphine + + + + + + + +
β-actin
42kDa
40

Figure 3L

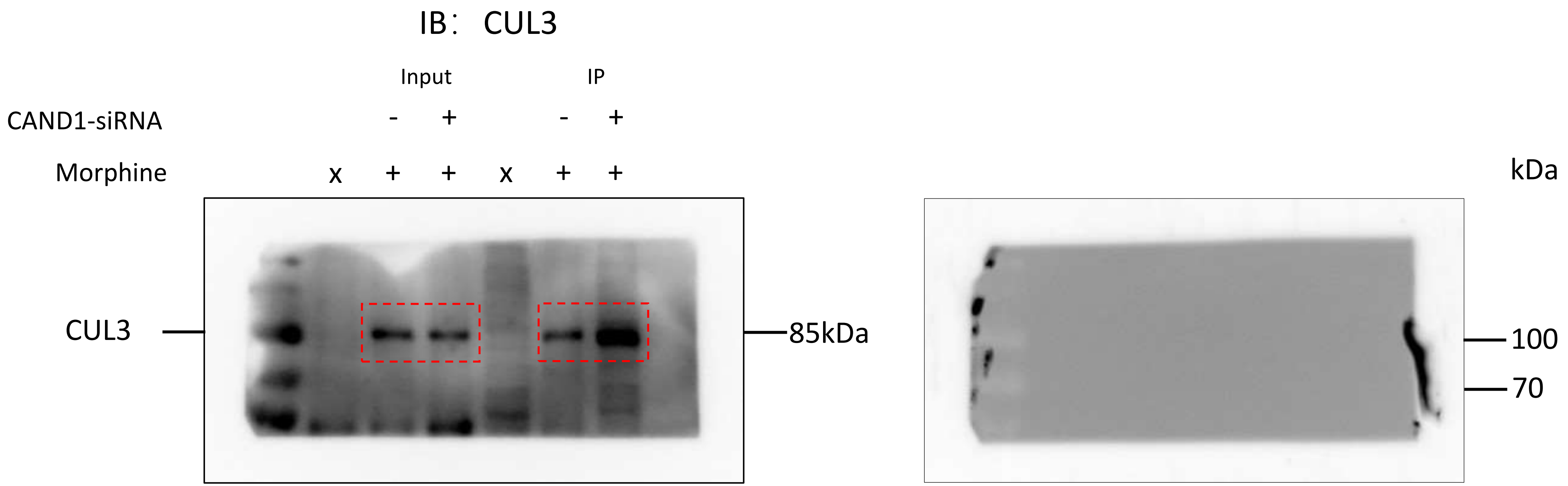

IB: CUL3
Input
IP
CAND1-siRNA
- + - +
Morphine
x + + x + +
kDa
CUL3
85kDa
100
70

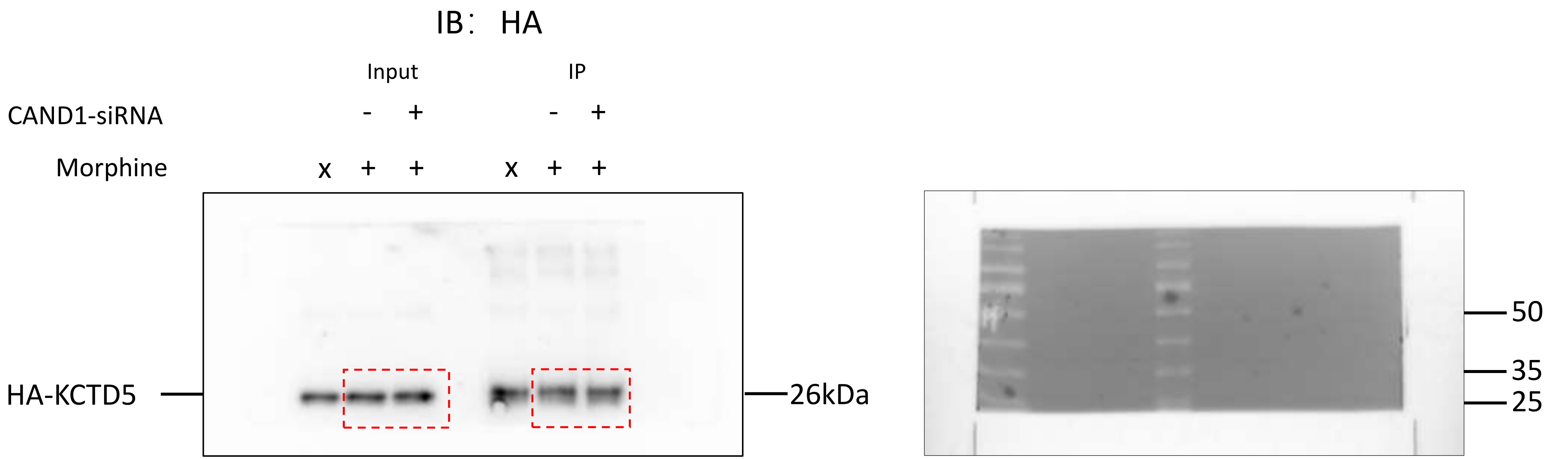

IB: HA
Input
IP
CAND1-siRNA
- + - +
Morphine
x + + x + +
HA-KCTD5
26kDa
50
35
25

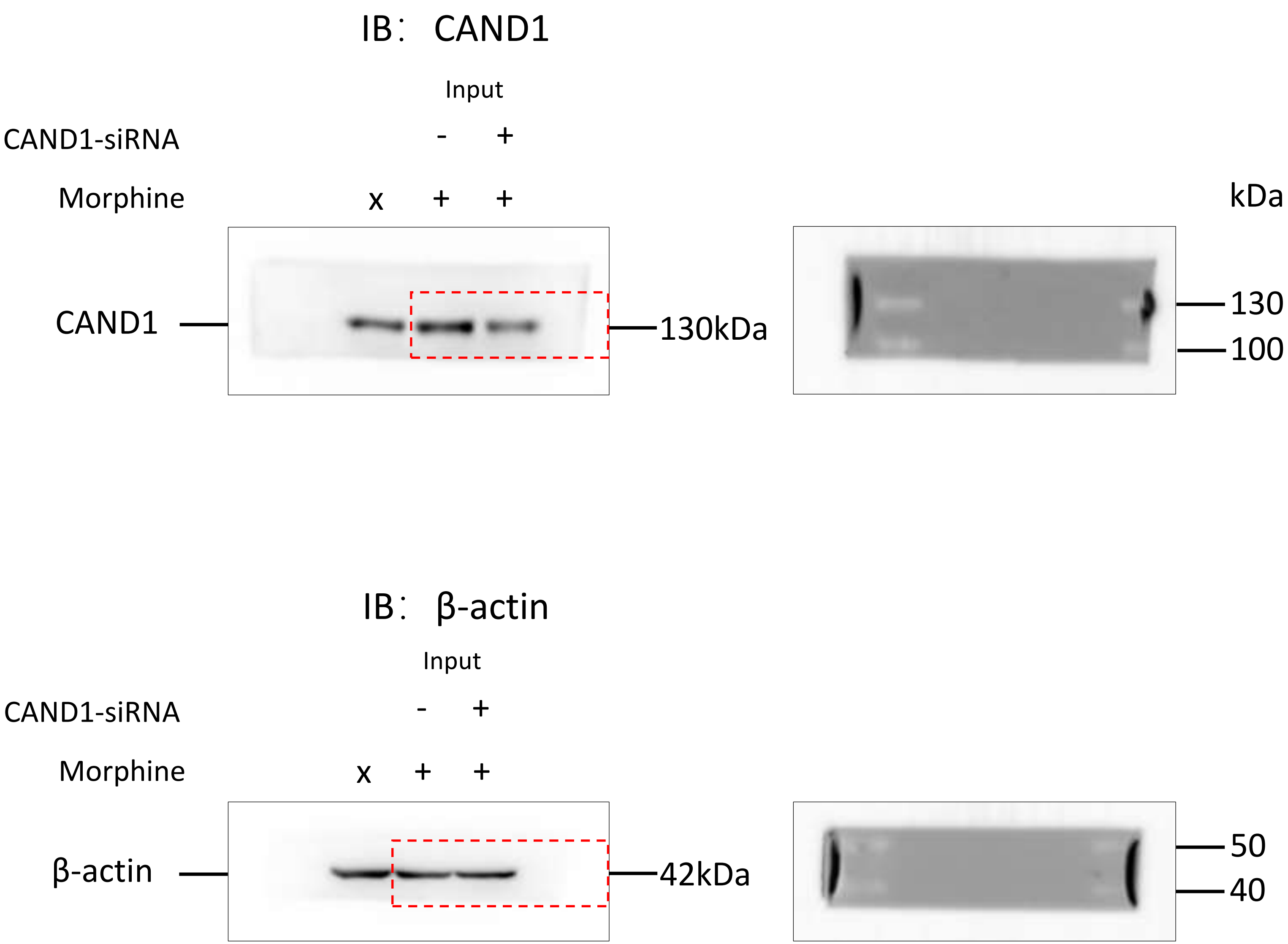
IB：CAND1
Input
CAND1-siRNA
-
+
Morphine
x
+
+
CAND1
130kDa
kDa
130
100
IB：β-actin
Input
CAND1-siRNA
-
+
Morphine
x
+
+
β-actin
42kDa
50
40

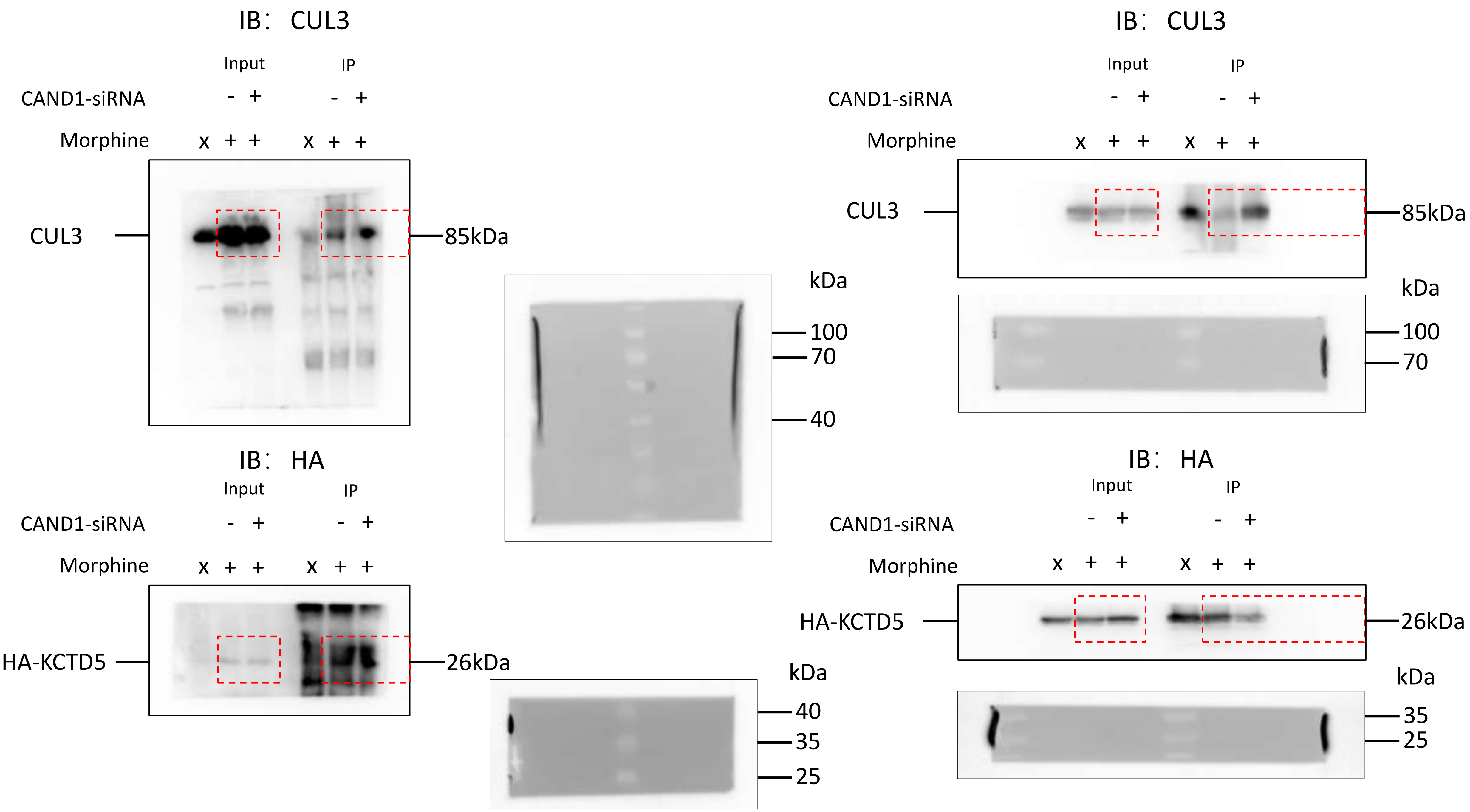
IB: CUL3
Input
IP
CAND1-siRNA
- + - +
Morphine
x + + x + +
CUL3
85kDa
kDa
100
70
40
IB: HA
Input
IP
CAND1-siRNA
- + - +
Morphine
x + + x + +
HA-KCTD5
26kDa
kDa
40
35
25
IB: CUL3
Input
IP
CAND1-siRNA
- + - +
Morphine
x + + x + +
CUL3
85kDa
kDa
100
70
IB: HA
Input
IP
CAND1-siRNA
- + - +
Morphine
x + + x + +
HA-KCTD5
26kDa
kDa
35
25

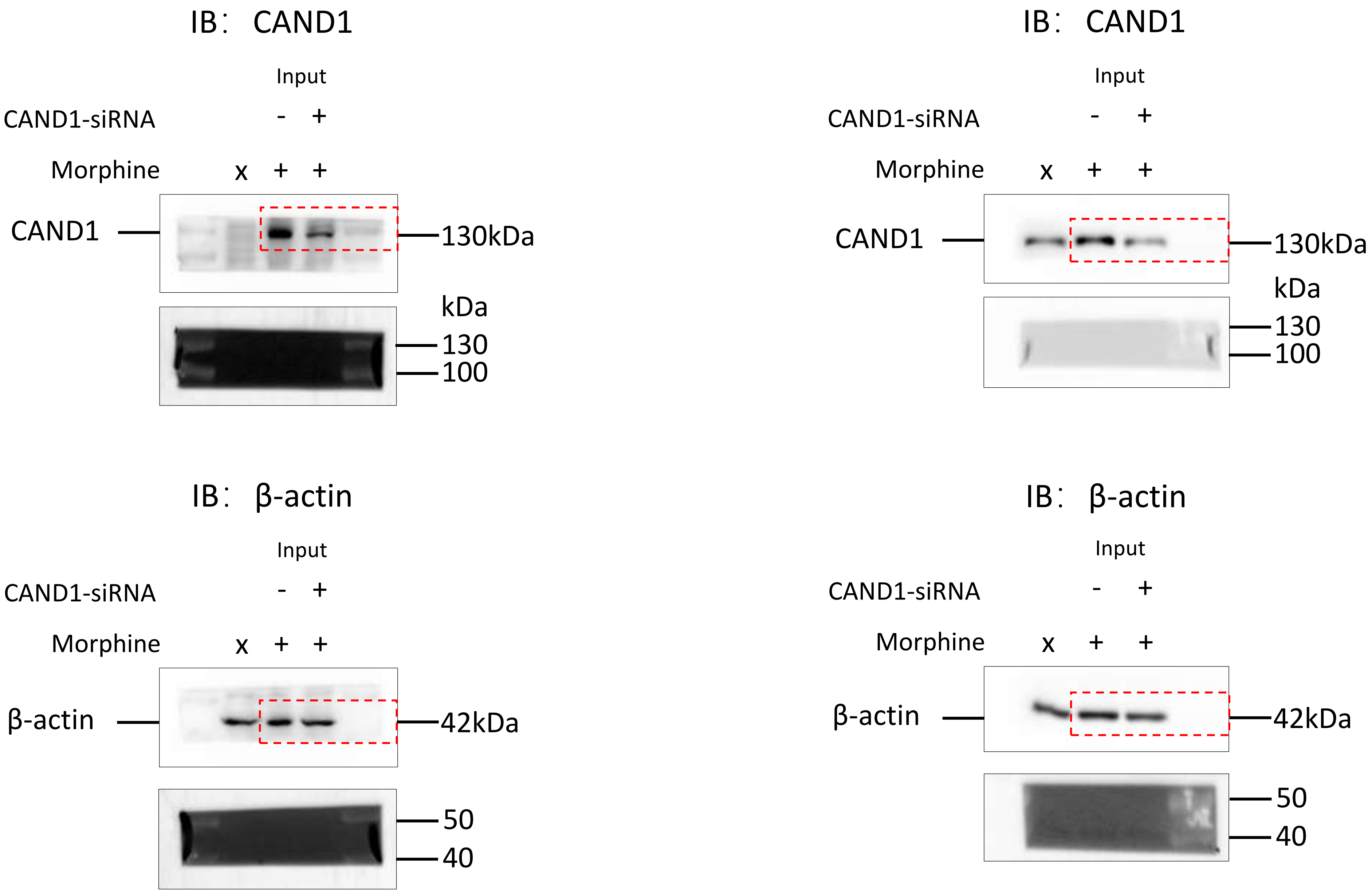
IB： CAND1
Input
CAND1-siRNA - +
Morphine x + +
CAND1
130kDa
kDa
130
100
IB： CAND1
Input
CAND1-siRNA - +
Morphine x + +
CAND1
130kDa
kDa
130
100
IB： β-actin
Input
CAND1-siRNA - +
Morphine x + +
β-actin
42kDa
50
40
IB： β-actin
Input
CAND1-siRNA - +
Morphine x + +
β-actin
42kDa
50
40

Figure 4F

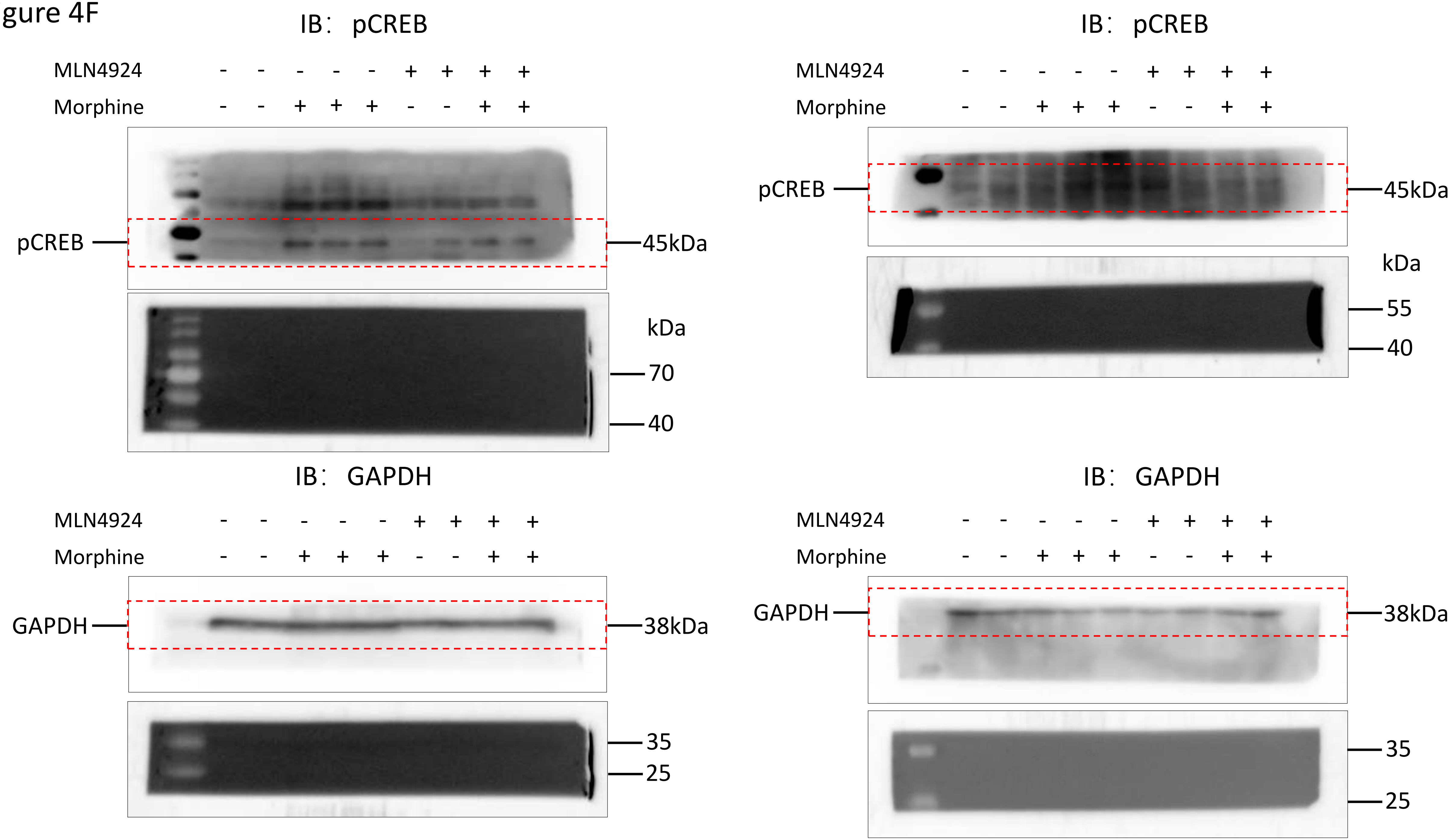
IB: pCREB
MLN4924 - - - - - + + + +
Morphine - - + + + - - + +
pCREB
45kDa
kDa
70
40
IB: pCREB
MLN4924 - - - - - + + + +
Morphine - - + + + - - + +
pCREB
45kDa
kDa
55
40
IB: GAPDH
MLN4924 - - - - - + + + +
Morphine - - + + + - - + +
GAPDH
38kDa
35
25
IB: GAPDH
MLN4924 - - - - - + + + +
Morphine - - + + + - - + +
GAPDH
38kDa
35
25

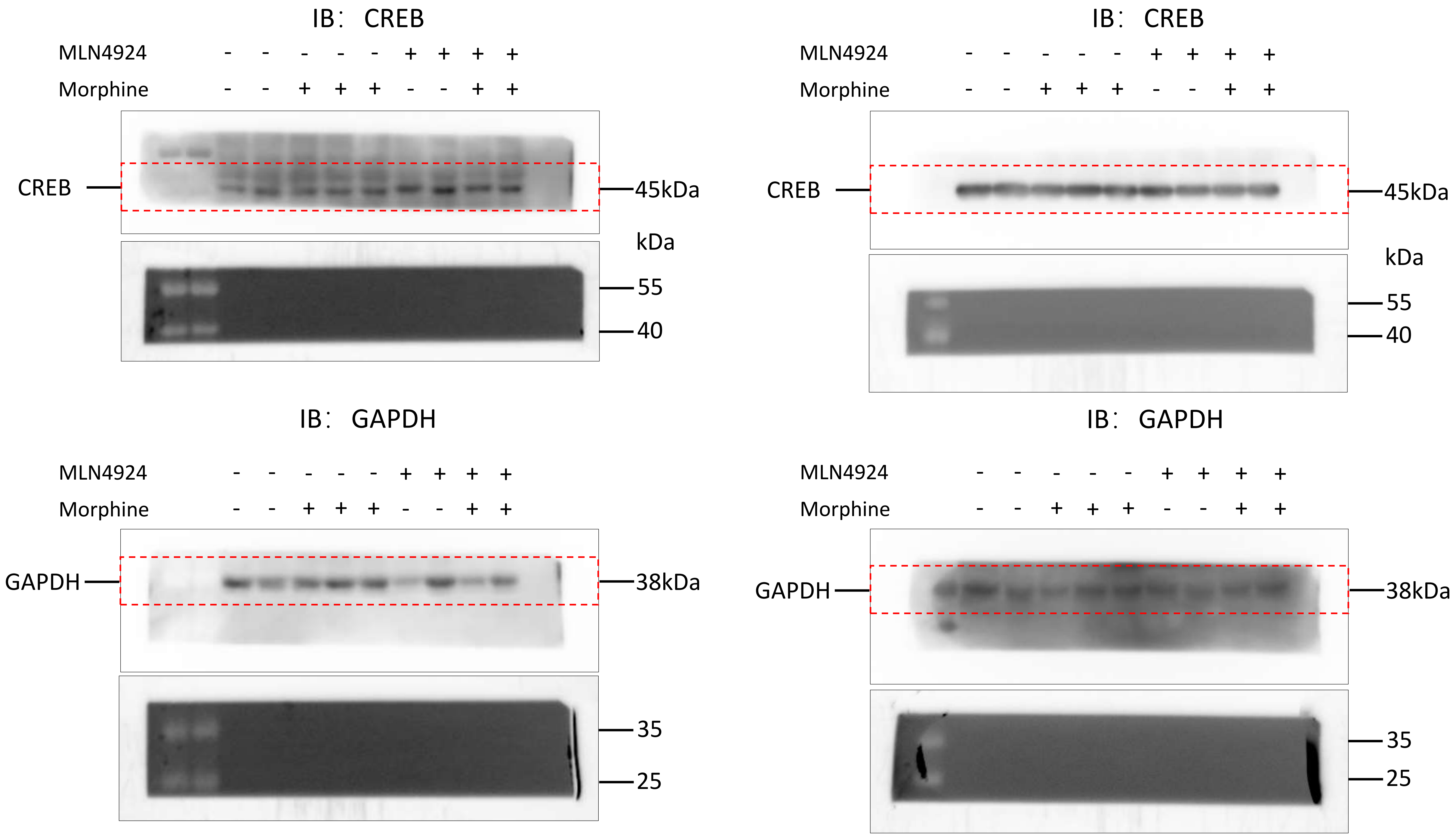
IB： CREB
MLN4924 - - - - - + + + +
Morphine - - + + + - - + +
CREB
45kDa
kDa
55
40
IB： CREB
MLN4924 - - - - - + + + +
Morphine - - + + + - - + +
CREB
45kDa
kDa
55
40
IB： GAPDH
MLN4924 - - - - - + + + +
Morphine - - + + + - - + +
GAPDH
38kDa
35
25
IB： GAPDH
MLN4924 - - - - - + + + +
Morphine - - + + + - - + +
GAPDH
38kDa
35
25

Figure 5F

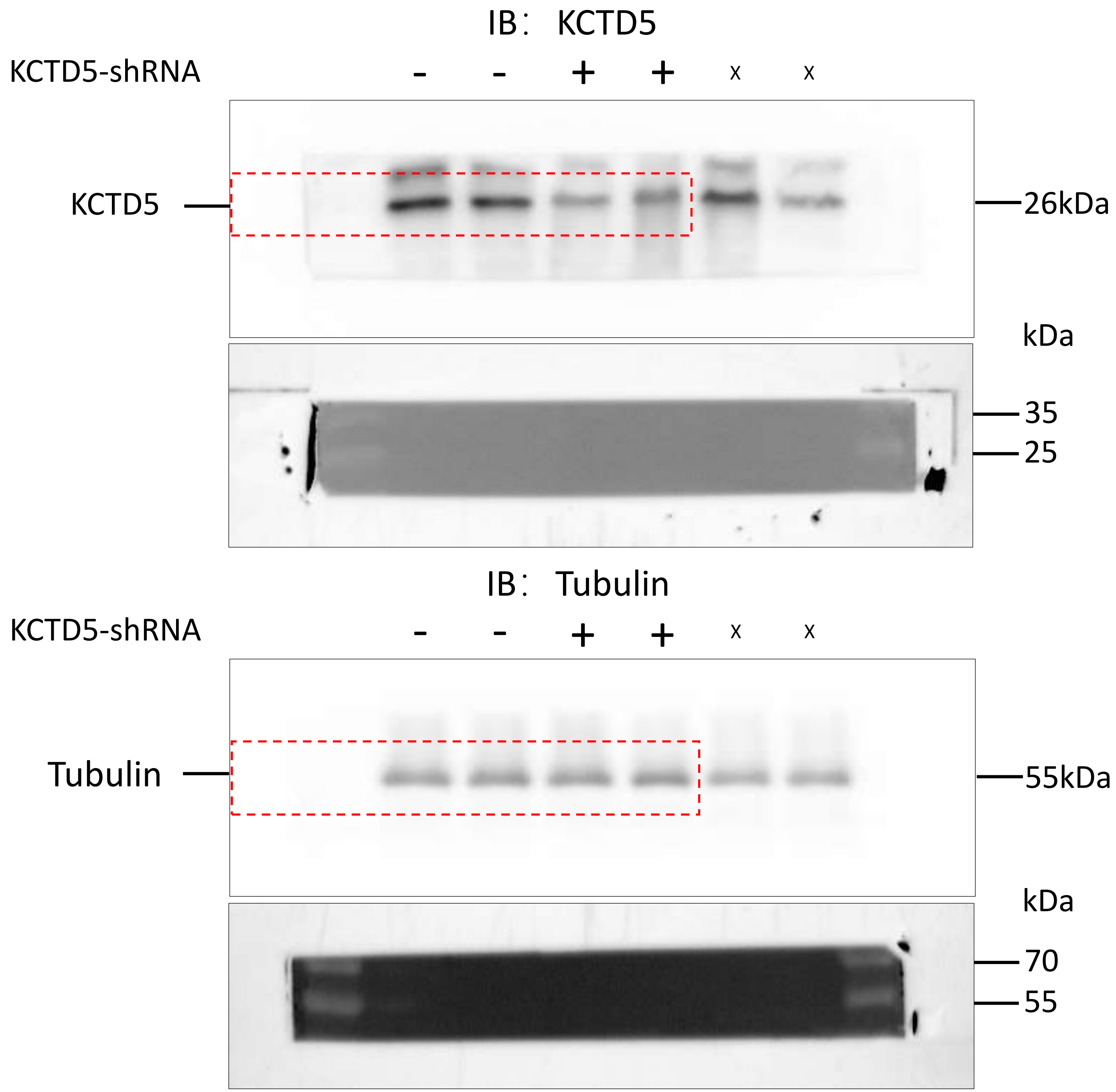
IB: KCTD5
KCTD5-shRNA
- - + + x x
KCTD5
26kDa
kDa
35
25
IB: Tubulin
KCTD5-shRNA
- - + + x x
Tubulin
55kDa
kDa
70
55

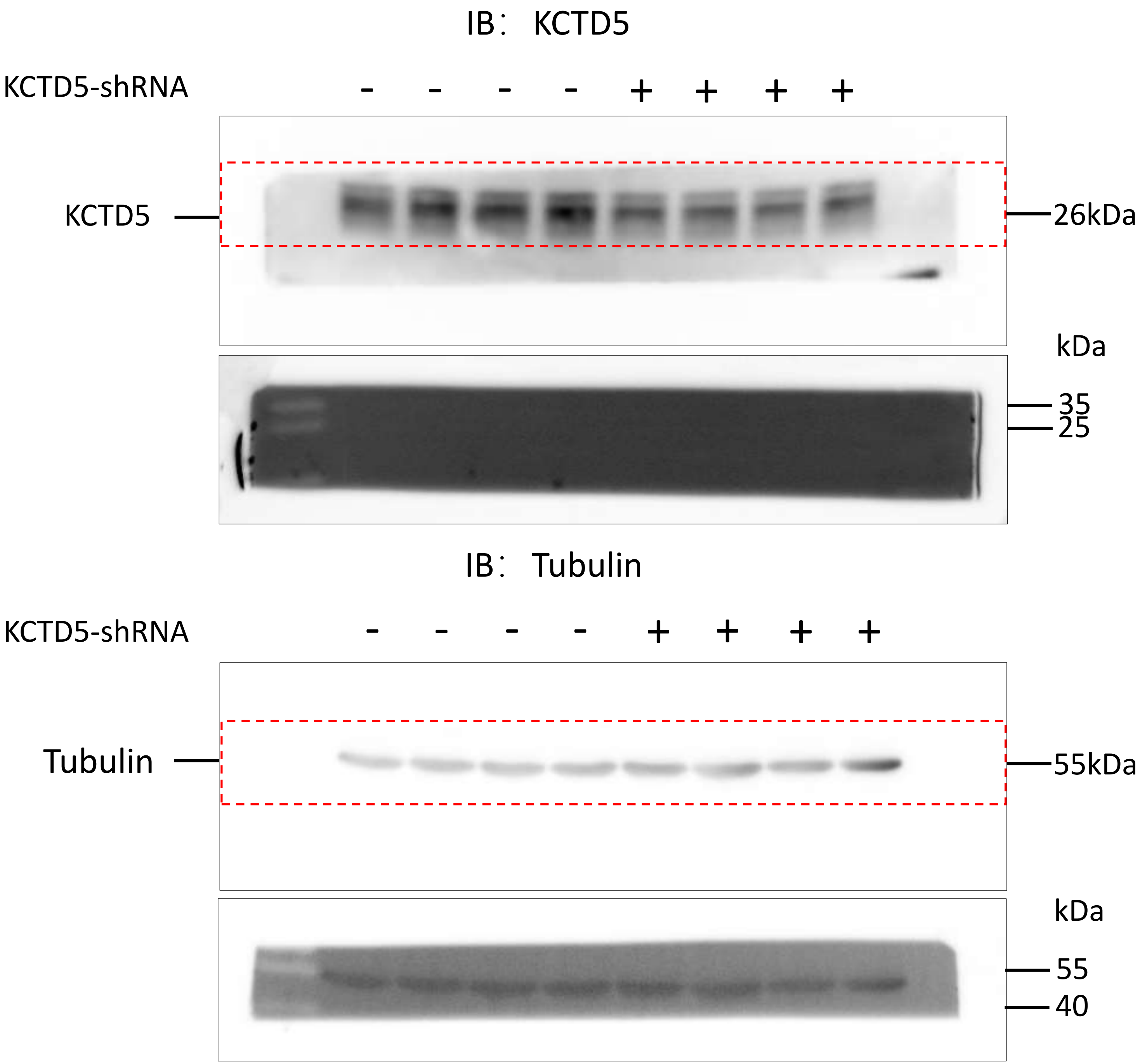
IB： KCTD5
KCTD5-shRNA
- - - - + + + +
KCTD5
26kDa
kDa
35
25
IB： Tubulin
KCTD5-shRNA
- - - - + + + +
Tubulin
55kDa
kDa
55
40

Figure 7F

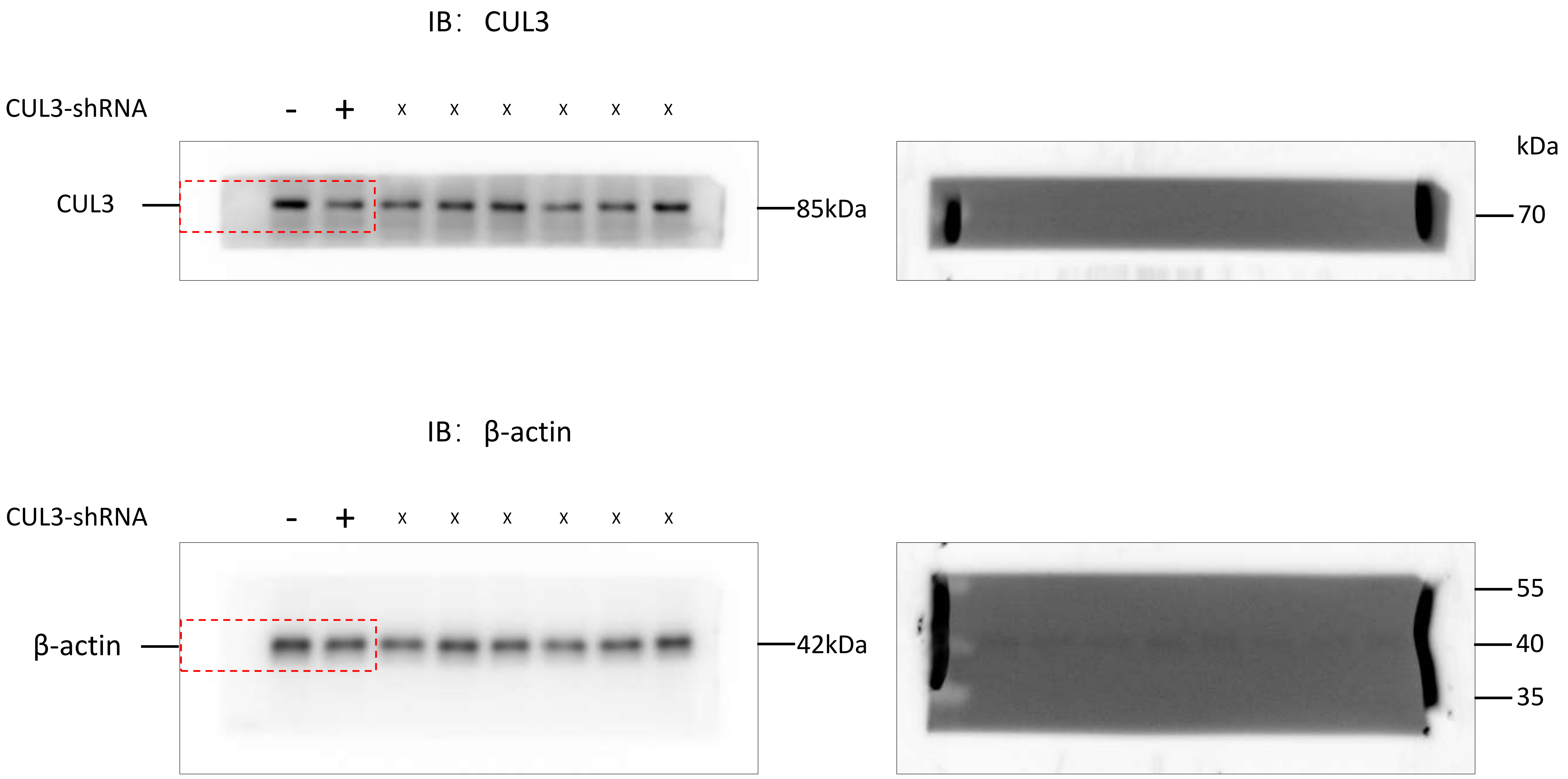
IB: CUL3
CUL3-shRNA
-
+
x
x
x
x
x
x
CUL3
85kDa
kDa
70
IB: β-actin
CUL3-shRNA
-
+
x
x
x
x
x
x
β-actin
42kDa
55
40
35

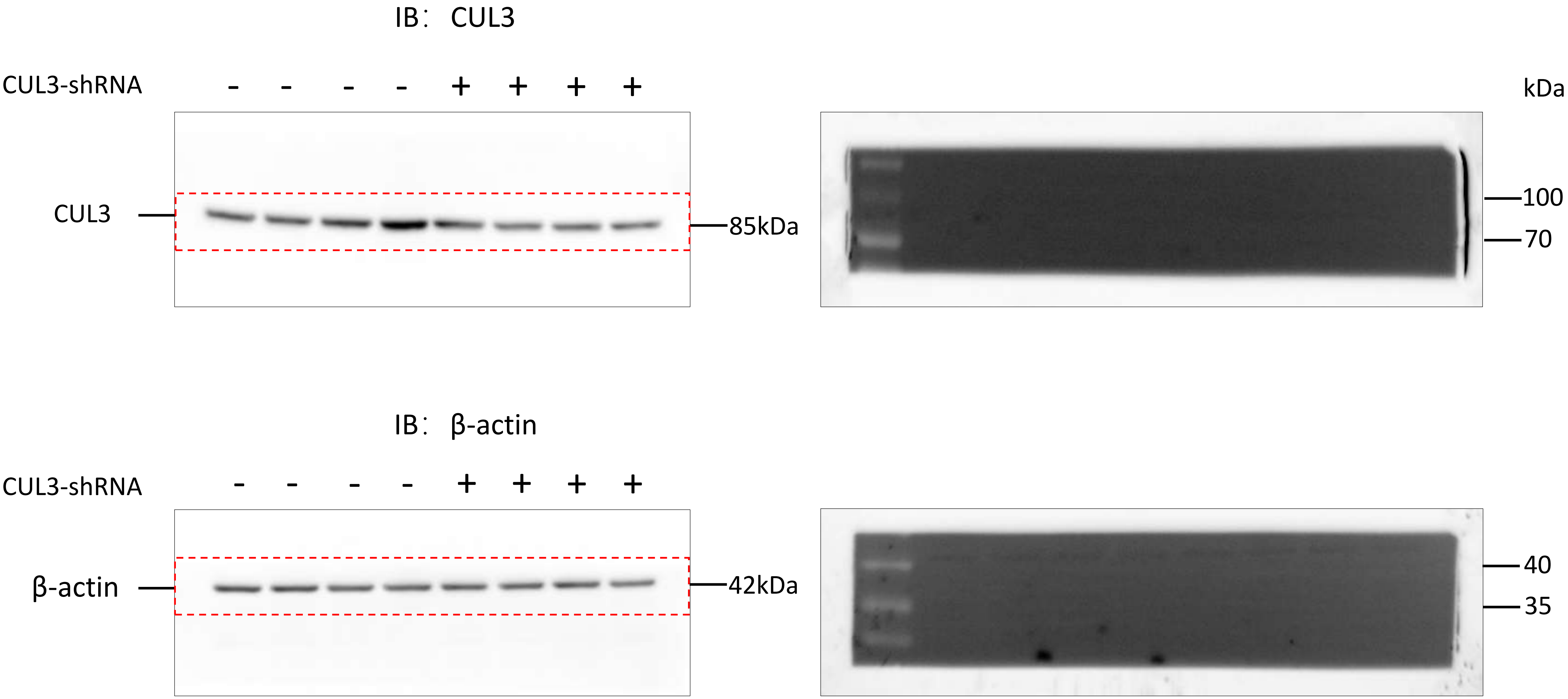
IB: CUL3
CUL3-shRNA - - - - + + + +
CUL3
85kDa
kDa
100
70
IB: β-actin
CUL3-shRNA - - - - + + + +
β-actin
42kDa
40
35

Figure S1C

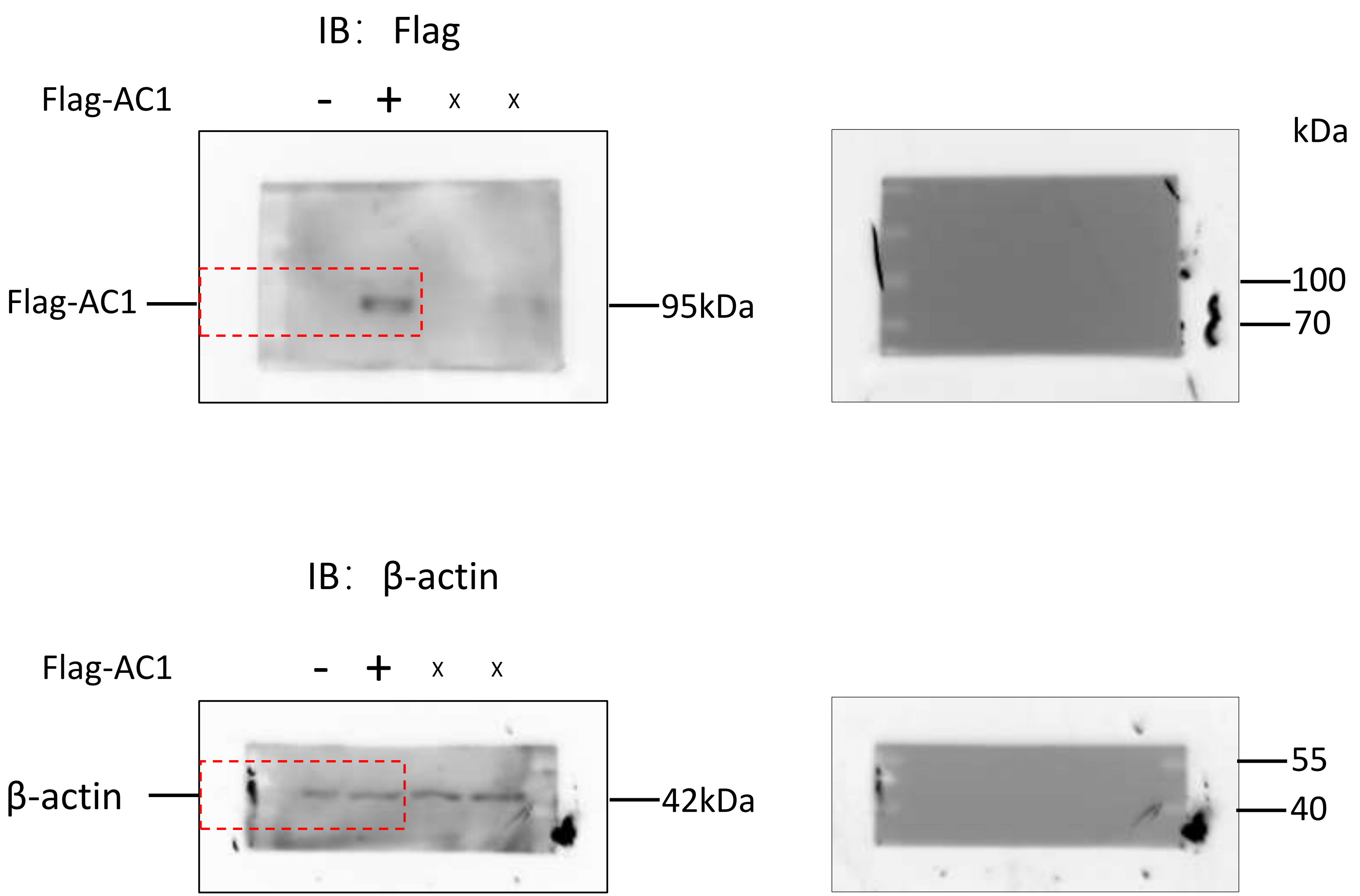
IB： Flag
Flag-AC1
- + x x
Flag-AC1
95kDa
kDa
100
70
IB： β-actin
Flag-AC1
- + x x
β-actin
42kDa
55
40

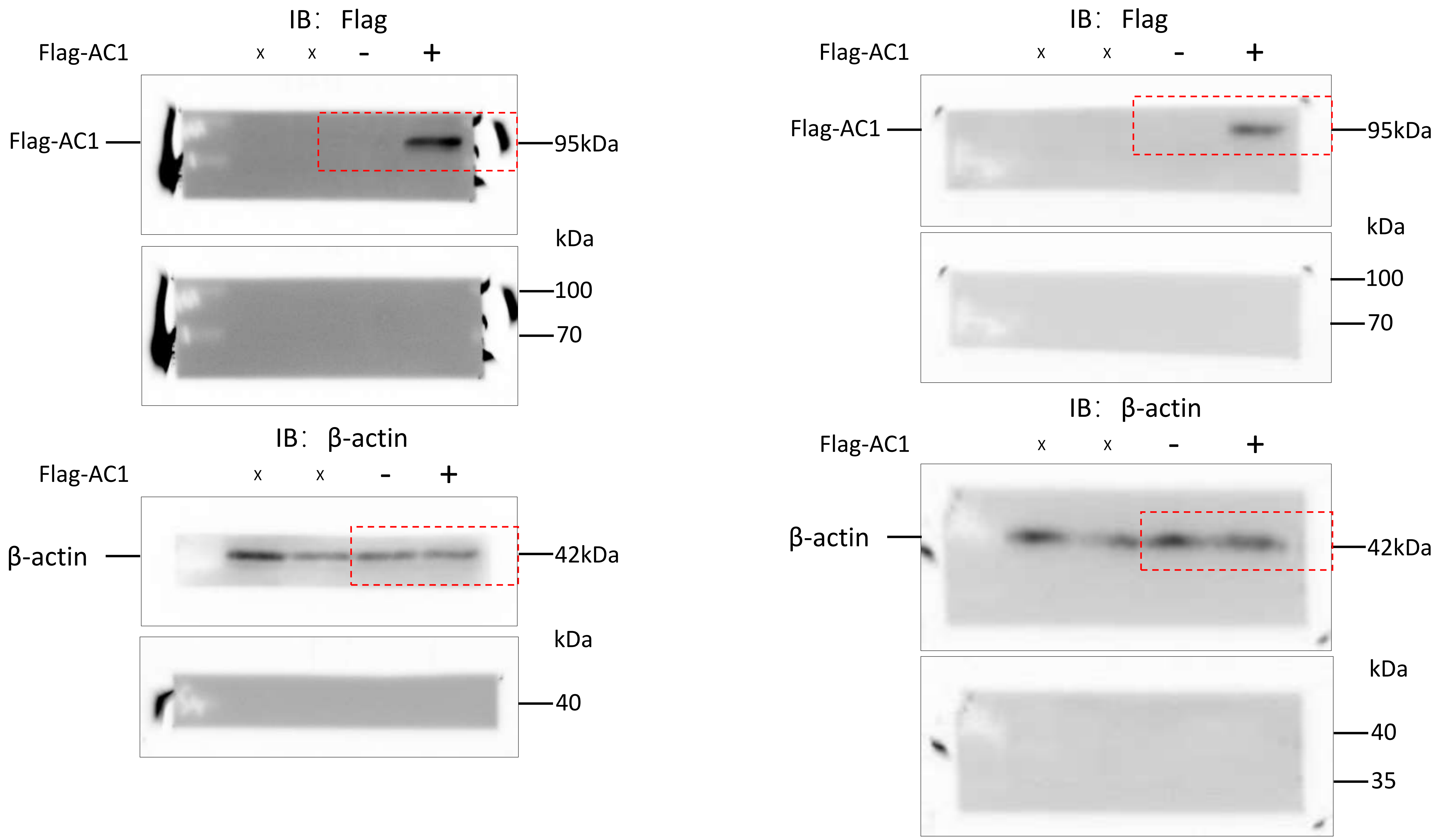
IB: Flag
Flag-AC1 x x - +
Flag-AC1
95kDa
kDa
100
70
IB: β-actin
Flag-AC1 x x - +
β-actin
42kDa
kDa
40
IB: Flag
Flag-AC1 x x - +
Flag-AC1
95kDa
kDa
100
70
IB: β-actin
Flag-AC1 x x - +
β-actin
42kDa
kDa
40
35

Figure S2C

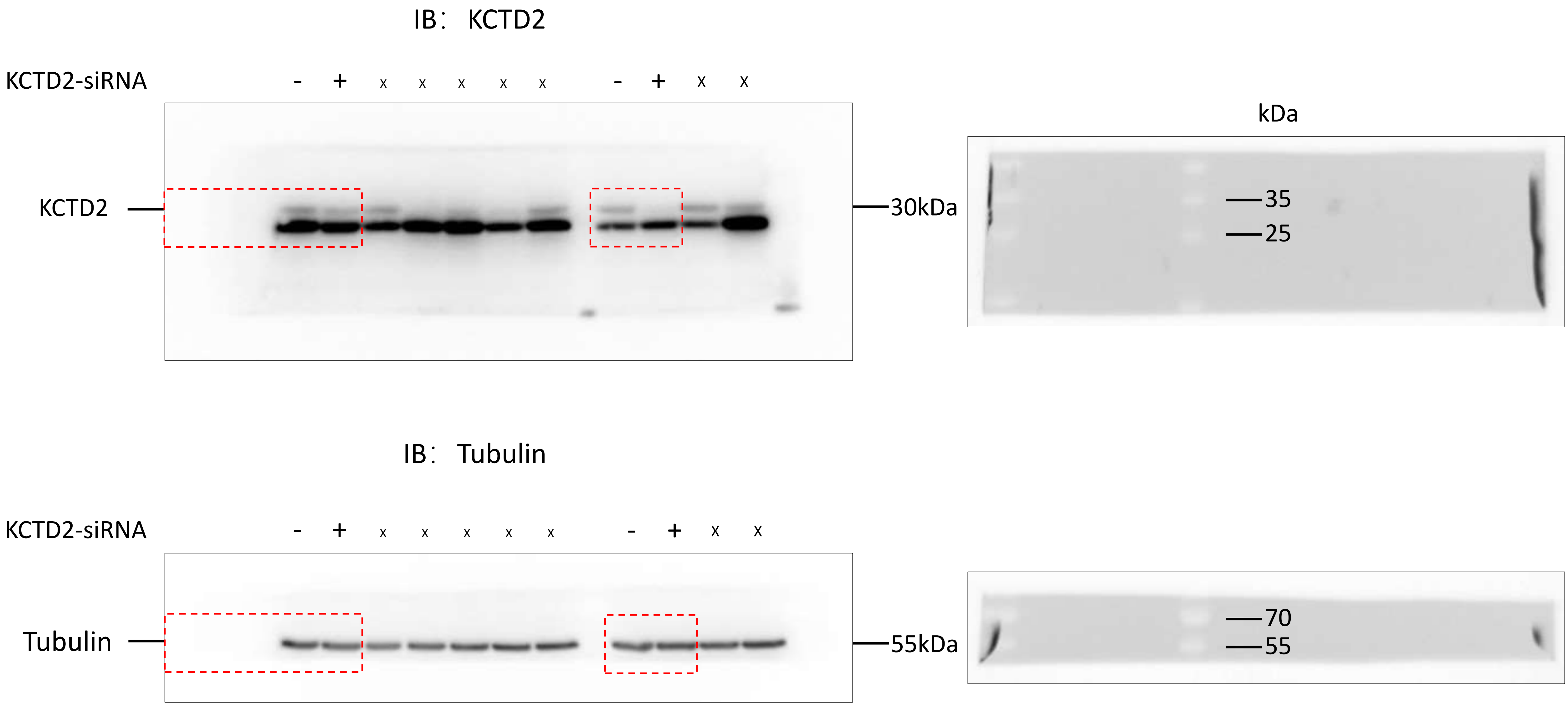
IB： KCTD2
KCTD2-siRNA
- + x x x x x - + x x
KCTD2
30kDa
kDa
35
25
IB： Tubulin
KCTD2-siRNA
- + x x x x x - + x x
Tubulin
55kDa
70
55

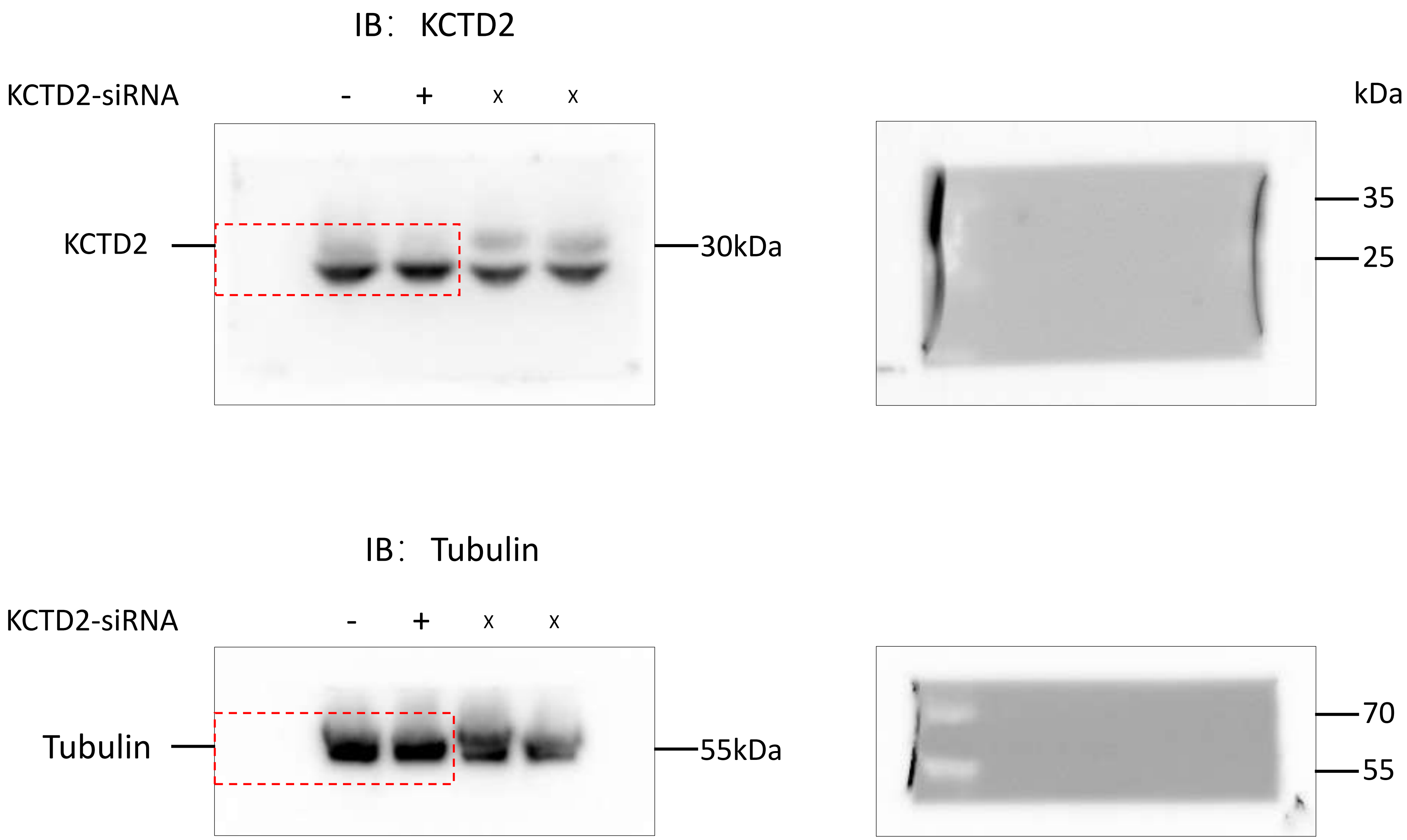
IB： KCTD2
KCTD2-siRNA
- + x x
kDa
KCTD2
30kDa
35
25
IB： Tubulin
KCTD2-siRNA
- + x x
Tubulin
55kDa
70
55

Figure S2D

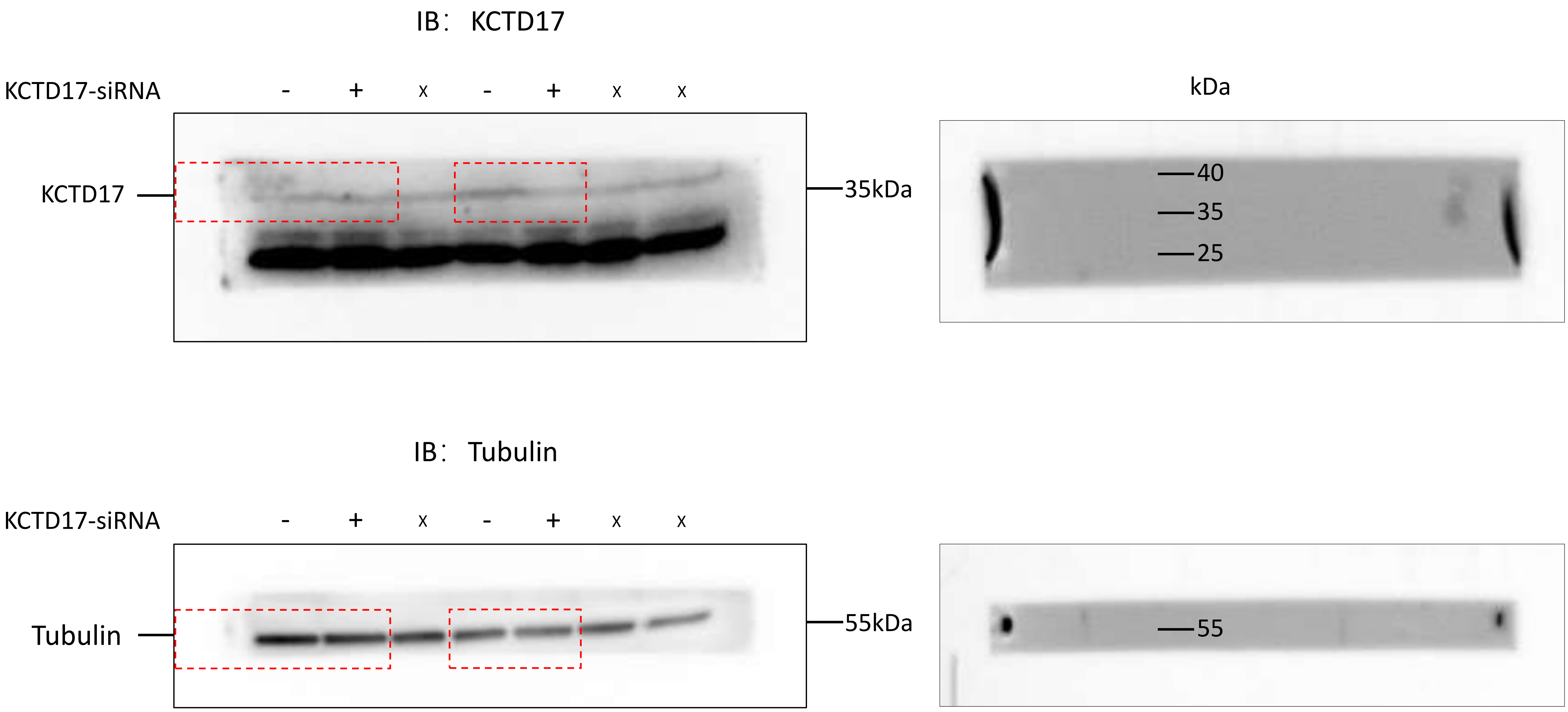
IB： KCTD17
KCTD17-siRNA
- + x - + x x
KCTD17
35kDa
kDa
40
35
25
IB： Tubulin
KCTD17-siRNA
- + x - + x x
Tubulin
55kDa
55

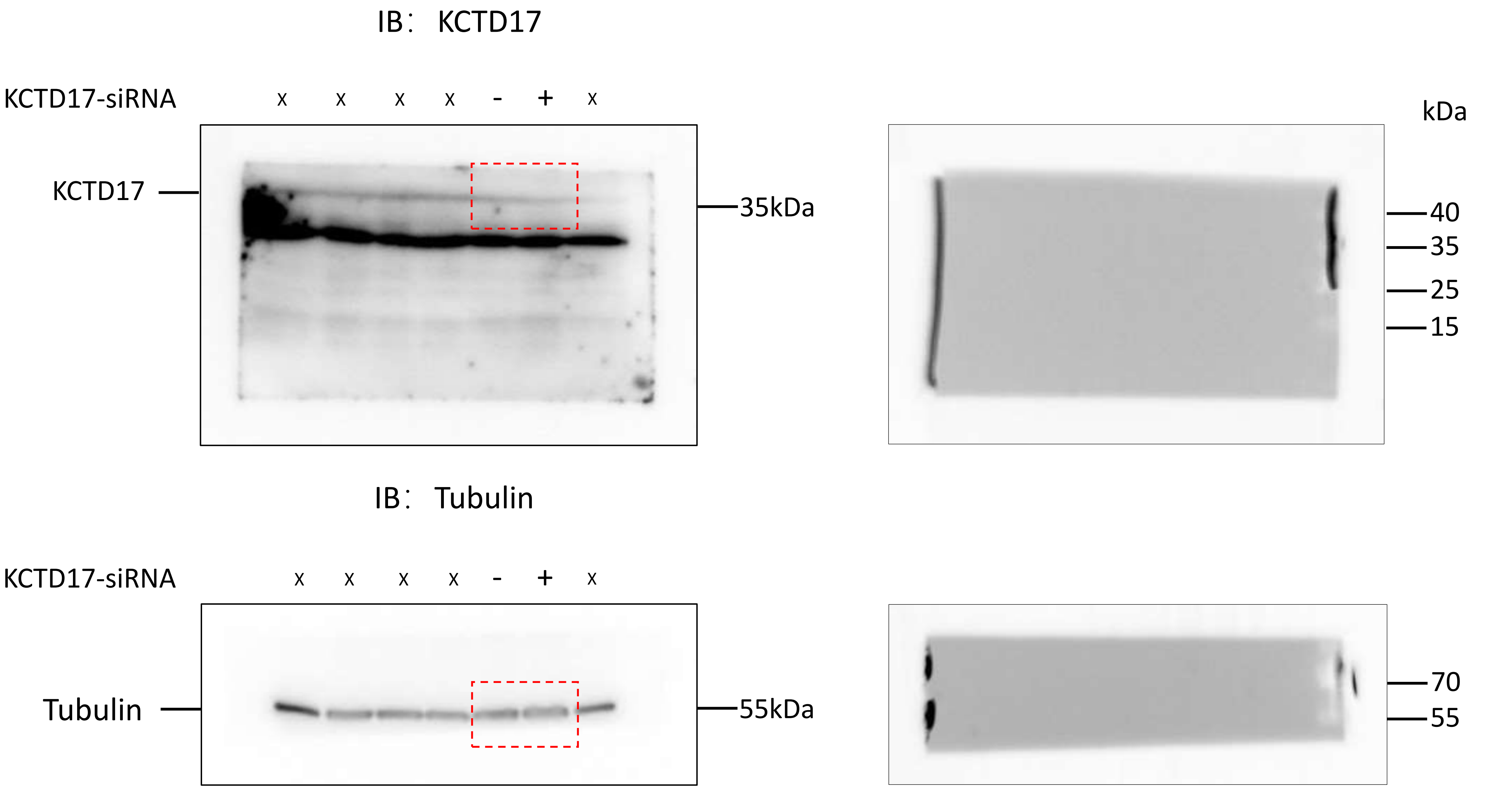
IB： KCTD17
KCTD17-siRNA x x x x - + x
KCTD17
35kDa
kDa
40
35
25
15
IB： Tubulin
KCTD17-siRNA x x x x - + x
Tubulin
55kDa
70
55

Figure S2G

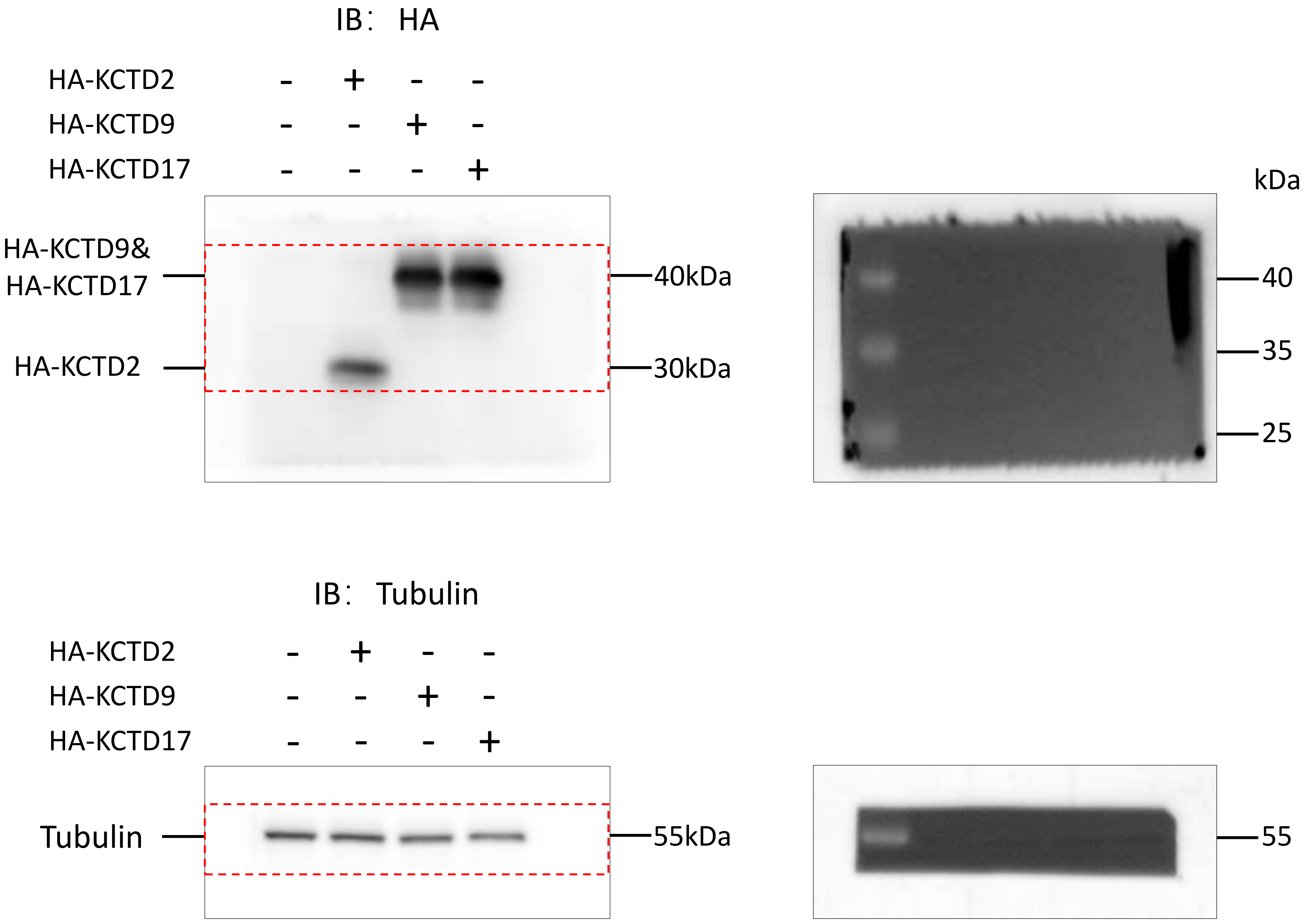

IB: HA
HA-KCTD2 - + - -
HA-KCTD9 - - + -
HA-KCTD17 - - - +
HA-KCTD9&
HA-KCTD17
HA-KCTD2
40kDa
30kDa
kDa
40
35
25
IB: Tubulin
HA-KCTD2 - + - -
HA-KCTD9 - - + -
HA-KCTD17 - - - +
Tubulin
55kDa
55

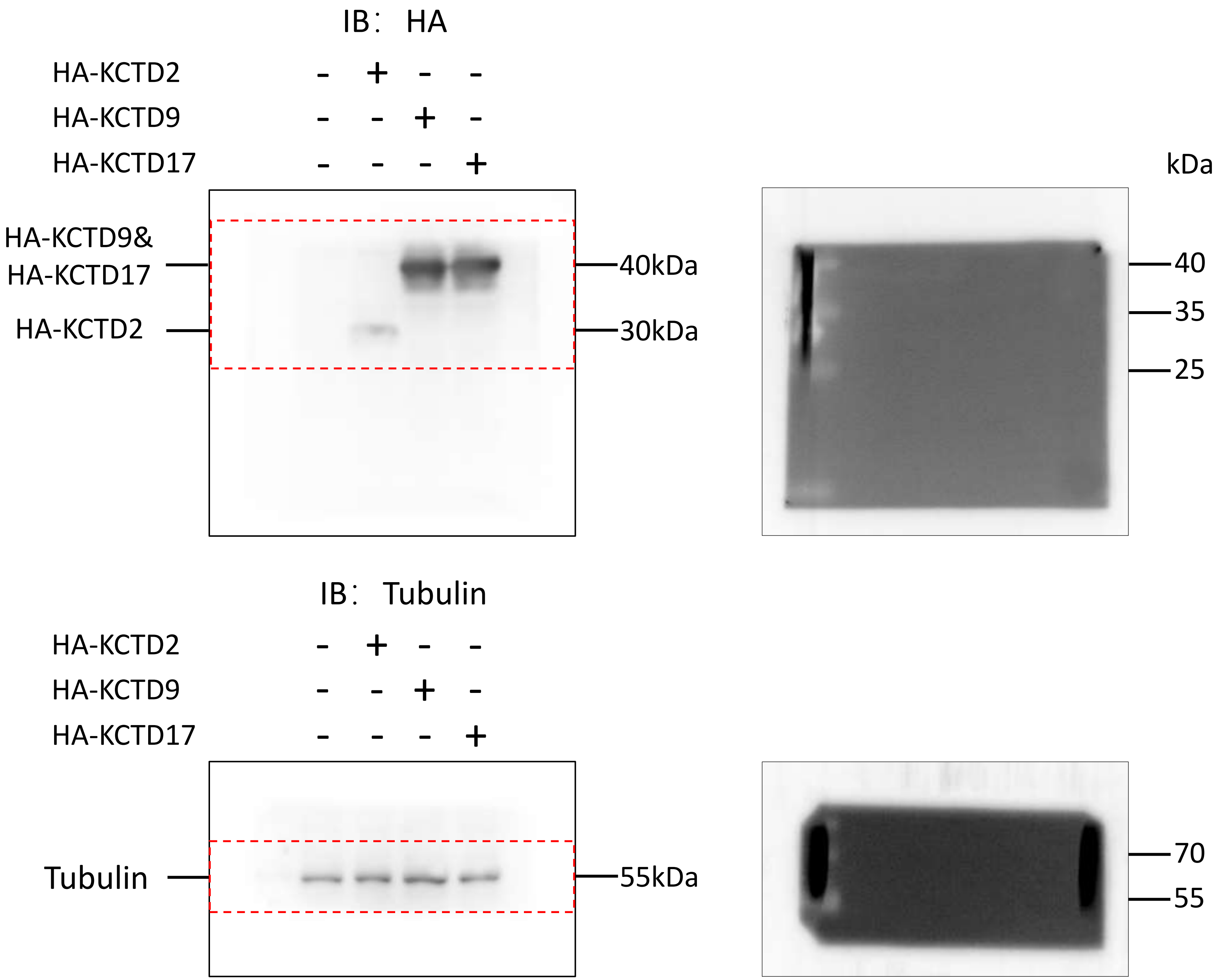
IB： HA
HA-KCTD2 - + - -
HA-KCTD9 - - + -
HA-KCTD17 - - - +
HA-KCTD9&
HA-KCTD17
HA-KCTD2
40kDa
30kDa
kDa
40
35
25
IB： Tubulin
HA-KCTD2 - + - -
HA-KCTD9 - - + -
HA-KCTD17 - - - +
Tubulin
55kDa
70
55

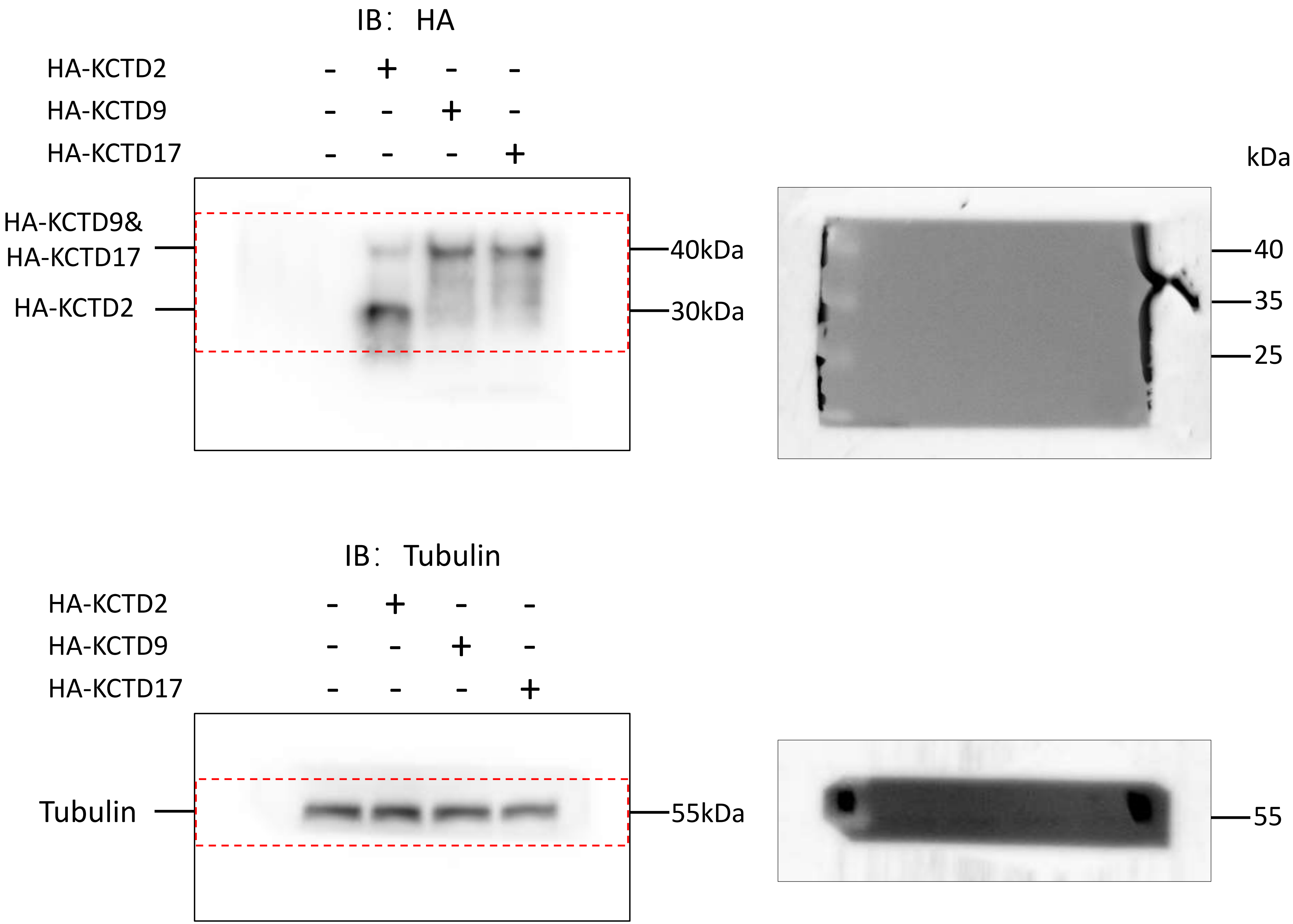
IB： HA
HA-KCTD2 - + - -
HA-KCTD9 - - + -
HA-KCTD17 - - - +
HA-KCTD9&
HA-KCTD17
HA-KCTD2
40kDa
30kDa
kDa
40
35
25
IB： Tubulin
HA-KCTD2 - + - -
HA-KCTD9 - - + -
HA-KCTD17 - - - +
Tubulin
55kDa
55

Figure S3C

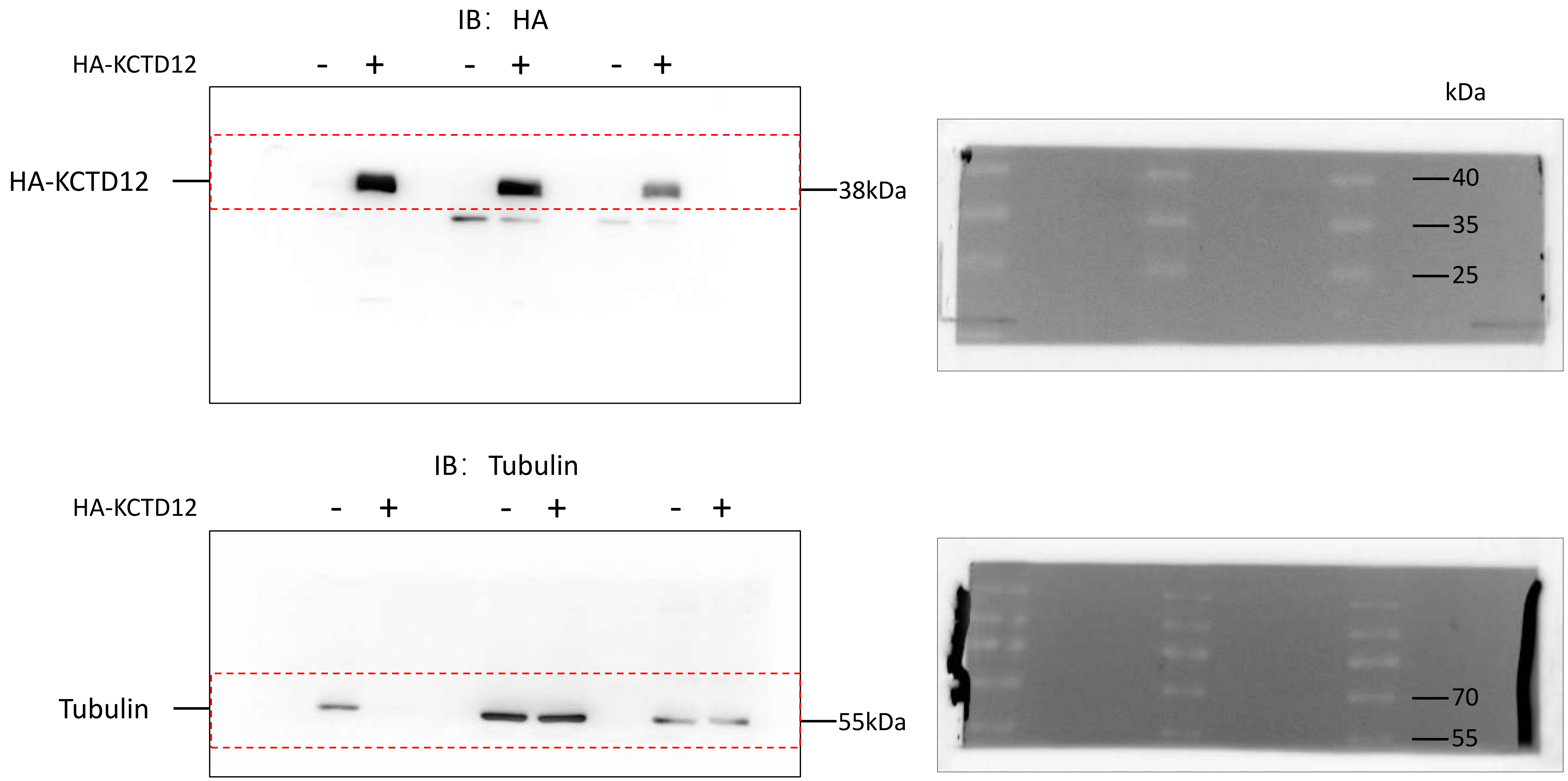
IB: HA
HA-KCTD12
- + - + - +
HA-KCTD12
38kDa
kDa
40
35
25
IB: Tubulin
HA-KCTD12
- + - + - +
Tubulin
55kDa
70
55

Figure S5

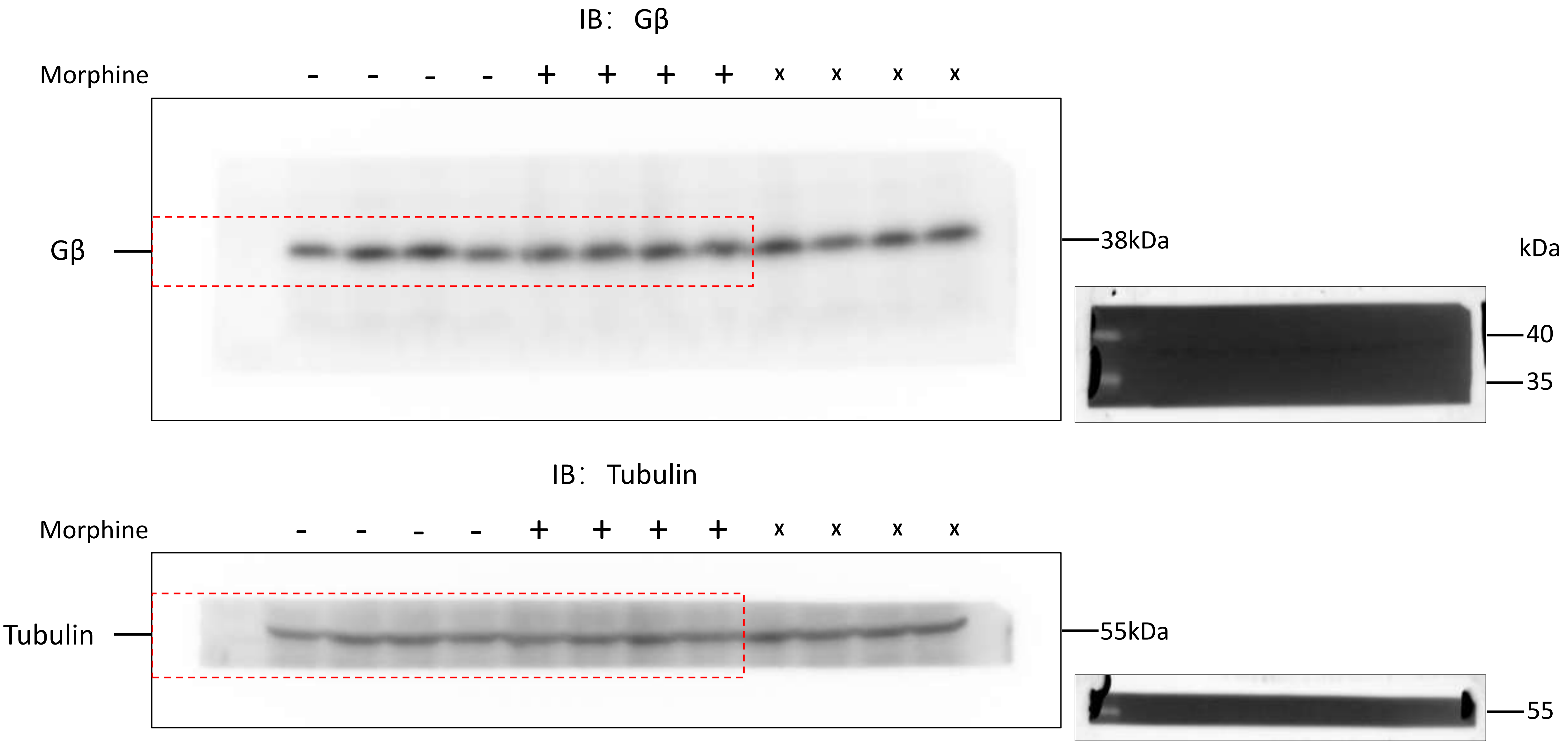
IB： Gβ
Morphine - - - - + + + + x x x x
Gβ
38kDa
kDa
40
35
IB： Tubulin
Morphine - - - - + + + + x x x x
Tubulin
55kDa
55
